# Global Dietary Database 2017: data availability and gaps on 54 major foods, beverages and nutrients among 5.6 million children and adults from 1220 surveys worldwide

**DOI:** 10.1136/bmjgh-2020-003585

**Published:** 2021-02-05

**Authors:** Victoria Miller, Gitanjali M Singh, Jennifer Onopa, Julia Reedy, Peilin Shi, Jianyi Zhang, Adeem Tahira, Masha L Shulkin Morris, Daniel P Marsden, Sarah Kranz, Sally Stoyell, Patrick Webb, Renata Micha, Dariush Mozaffarian

**Affiliations:** 1 Friedman School of Nutrition Science and Policy, Tufts University, Boston, Massachusetts, USA; 2 Penn State University, State College, Pennsylvania, USA; 3 University of Michigan Medical School, Ann Arbor, Michigan, USA; 4 The George Washington University School of Medicine and Health Sciences, Washington, District of Columbia, USA; 5 University of Minnesota, Minneapolis, Minnesota, USA

**Keywords:** epidemiology, nutrition, public health

## Abstract

**Background:**

We aimed to systematically identify, standardise and disseminate individual-level dietary intake surveys from up to 207 countries for 54 foods, beverages and nutrients, including subnational intakes by age, sex, education and urban/rural residence, from 1980 to 2015.

**Methods:**

Between 2008–2011 and 2014–2020, the Global Dietary Database (GDD) project systematically searched for surveys assessing individual-level intake worldwide. We prioritised nationally or subnationally representative surveys using 24-hour recalls, Food-Frequency Questionnaires or short standardised questionnaires. Data were retrieved from websites or corresponding members as individual-level food group microdata or aggregate stratum-level data. Standardisation included quality assessment; data cleaning; categorising of foods and nutrients and their units; aggregation by demographic strata and energy adjustment.

**Results:**

We standardised and incorporated 1220 surveys into the final GDD 2017 database, together represented 188 countries and 99.0% of the world’s population in 2015. 72.1% were nationally, 17.0% subnationally, and 10.9% community-level representative. 41.2% used Food-Frequency Questionnaires; 23.4%, 24-hour recalls; 15.8%, Demographic Health Survey questionnaires; 13.1%, biomarkers and 6.4%, household surveys. 73.9% of surveys included data on children; 52.2%, by urban and rural residence; and 30.2%, by education. Most surveys were in high-income countries, followed by sub-Saharan Africa and Asia. Most commonly ascertained foods were fruits (N=803 surveys), non-starchy vegetables (N=787) and sugar-sweetened beverages (N=440); and nutrients, sodium (N=343), energy (N=256), calcium (N=224) and fibre (N=200). Least available data were on iodine, vitamin A, plant protein, selenium, added sugar and animal protein.

**Conclusions:**

This systematic search, retrieval and standardised effort provides the most comprehensive empirical evidence on dietary intakes across and within countries worldwide.

**Key questions**

**What is already known?**

- Comparable and standardised global data on intakes of foods, beverages and nutrients relevant to maternal–child health and chronic diseases have not traditionally been available across nations nor key subnational subgroups.

**What are the new findings?**

- Through systematic searches and collaboration with investigators worldwide, we retrieved and standardised 1220 surveys of nationally or subnationally representative data on individual-level dietary intakes from 188 countries/territories around the world.
- Most nationally or subnationally representative surveys were identified in high-income countries, followed by sub-Saharan Africa, Asia, Former Soviet Union, Latin America/Caribbean. Middle East/North Africa and South Asia were more data sparse.
- Among foods, data on fruits, vegetables and sugar-sweetened beverages were most available; among nutrients, on sodium, energy, calcium and fibre. Data on iodine, vitamin A, plant protein, selenium, added sugar and animal protein were most sparse.
- Less than one-third of surveys had dietary intake data on infants (age 0 to <2 years), young children (2 to <6 years), school age children (6 to 10 years), older adults (70+ years) and pregnant/lactating women or by education level.

**What do the new findings imply?**

- These identified, collected and standardised data in the Global Dietary Database 2017 provide most comprehensive empirical evidence on dietary intakes across and within countries worldwide.

## Introduction

Diet is critical to both human^1 2^ and planetary health.^3 4^ Comprehensive and reliable evidence on individual dietary intakes in all nations of the world is essential for evaluating diet-related burdens for maternal–child health (MCH) and non-communicable diseases (NCDs), as well as for understanding population-level disparities, food costs and affordability, environmental sustainability, and progress toward key aims. For example, the recognition that global diets are relevant to 12 of 17 of the United Nations (UN) Sustainable Development Goals has led to ongoing planning for the first-ever UN Food Systems Summit, scheduled for 2021.^5^ Given large potential variation within nations, such data are also crucial to provide empirical evidence of dietary habits in key subgroups, such as by age, sex, socioeconomic status and urban or rural residence. Data on habitual dietary intakes in diverse world regions and subpopulations are also essential to inform the potential impact of acute shocks such as the COVID-19 pandemic.

Unfortunately, little data have been systematically identified, collated and standardised on dietary habits worldwide. Available instruments have assessed food commodities or household expenditures, which are not reflective of individual dietary intakes; for example, the UN Food and Agriculture Organization (FAO) Food Balance Sheets (FBS)^6^ of estimated national food availability, or the World Bank’s Living Standard Measurement Survey^7^ and other household expenditure instruments which assess household-level food purchasing (but not foods produced by the household, purchased outside the home or actual individual intakes). These available data sources also do not assess heterogeneity within nations, such as by demographic subgroups that may vary in both dietary intakes and disease risk. Numerous national or subnational health and nutrition surveys at the individual level have been conducted around the world, but these are seldom standardised or comparable across countries, time, dietary factors or demographic groups,^8^ and many are not publicly available.

To address these gaps, the Global Dietary Database (GDD) project was created to comprehensively identify, compile and standardise individual-level data on dietary factors relevant to health.^9^ The first iteration, GDD 2010, developed systematic methods to compile data from 527 dietary surveys (including urine biomarker surveys) from 116 countries/territories, representing 88.7% of the global adult population in 2010.^10^ The GDD 2010 represented a major advancement over previously available data, facilitating novel assessments of global dietary habits, trends and patterns,^11–15^ burdens of diet-related illness^16–19^ and diet-related sustainability concerns.^20 21^ In addition, the GDD 2010, together with its associated systematic characterisation of diet-disease aetiological effects and optimal intake levels,^22^ formed the foundation for the 2010 and 2013 Global Burden of Diseases Study risk estimates of diet-related health burdens.^16 23^ These findings confirmed for the first time, for example, that poor diet has overtaken tobacco smoking as the leading cause of preventable death in the world.^23^

Yet, key limitations remained. GDD 2010 focused on major dietary risks for NCDs, but excluded other dietary components, such as micronutrients, relevant for MCH. GDD 2010 also focused on adults (age ≥20 years), with little data on dietary intakes in children or youth. While GDD 2010 provided the first global data stratified by age and sex within nations, it did not include other subnational stratifiers likely to influence diets such as socioeconomic status or urban versus rural residence. GDD 2010 also identified data sparsity in certain regions of the world. To address these gaps, update available data, and advance the characterisation of dietary intakes worldwide, the GDD 2017 systematically identified, collected and standardised additional dietary surveys, including new information on many more foods, beverages and nutrients; an expanded age focus to include infants, children and adolescents; and further joint stratification by age, sex, education level, urban/rural residence and pregnancy/lactation status within nations. This analysis reports on data availability, and corresponding gaps, of global dietary data.

## Methods

### Prioritisation of dietary surveys

Our methods for GDD 2010 have been reported.^10 14 15 17 24^ Briefly, we conducted systematic searches of multiple electronic databases and extensive personal communications with experts and authorities worldwide to identify and obtain individual-level dietary surveys globally. We focused on quantitative data on dietary consumption of 21 foods, beverages and nutrients in 16 age-specific and sex-specific subgroups among adults (age ≥20 years) in up to 116 nations across 21 geographical regions between 1980 and 2010. For GDD 2017, we performed additional systematic electronic searches together with extensive communications with 644 data owners worldwide to identify further public and nonpublic data sources on individual-level dietary intakes. We prioritised nationally or subnationally representative surveys, with a special focus on previously identified data sparse low-income and middle-income countries.

We searched for surveys that collected quantitative dietary intake information on one or more of 54 foods, beverages, nutrients or dietary indices (table 1). These were selected and defined based on evidence for relationships with MCH or NCDs as well as clinical and policy interests in their intakes. We also searched for surveys on four additional dietary factors (animal protein excluding dairy protein, dairy protein, glycaemic index and glycaemic load), but identified too few available surveys to include these in the GDD 2017. We prioritised surveys with individual-level assessments using standardised 24-hour recalls, food-frequency questionnaires (FFQ) or short standardised questionnaires (eg, Demographic Health Survey (DHS) questionnaires). Household-level surveys were considered if individual-level surveys were not available in a country. For assessment of dietary sodium and iron, we also searched for and included biomarker surveys measuring 24-hour urinary sodium excretion or blood haemoglobin concentrations.

**Table 1:** Definitions and units of dietary variables and biomarkers included in the GDD 2017

Dietary factor	Unit	Preferred definition	Alternative definition
Fruits	g/day	Total fruit intake, including fresh, frozen, cooked, canned or dried fruit, excluding fruit juices and salted or pickled fruits.	Total fruit intake including fruit juices, nuts/seeds, vegetables, salted/pickled, preserved and processed fruits (jams).
Non-starchy vegetables	g/day	Total vegetable intake, including fresh, frozen, cooked, canned or dried vegetables. This definition excludes salted or pickled vegetables, vegetable juices, starchy vegetables (eg, potatoes, taro, cassava, manioc, yucca, corn, peas) and legumes (beans and lentils).	Total vegetable intake including vegetable juices, starchy vegetables, nuts/legumes, nuts/beans, beans/legumes, salted/pickled vegetables and salted/pickled beans/legumes.
Potatoes	g/day	Total intake of white potatoes, including cooked (eg, boiled, baked, mashed, fried), frozen, canned, dehydrated potatoes. This definition includes french fries, chips and crisps. This definition excludes sweet potatoes and yams.	Includes other starchy vegetables.
Other starchy vegetables	g/day	Total intake of non-potato starchy vegetables, including fresh, frozen, cooked, canned, or dehydrated starchy vegetables. Examples of starchy vegetables include green peas, corn (including corn flour/corn meal), yam, sweet potatoes, taro, plantain, cassava, manioc, tannier (yautia), jicama, and water chestnuts. This definition excludes white potatoes.	Includes starchy fruits or potatoes and starches refined from starchy vegetables. May include non-starchy vegetables such as carrots and/or fruits such as mangos, sweet potatoes and hard squashes.
Beans and legumes	g/day	Total intake of beans and legumes (beans, lentils), including fresh, frozen, cooked, canned, or dried beans/legumes. This definition excludes peanuts and peanut butter. This definition includes soybeans but excludes soy milk and soy protein.	Includes nuts/seeds, soy protein, soy products, peanuts and peas.
Nuts and seeds	g/day	Total intake of tree nuts (eg, walnuts, almonds, hazelnuts, pecans, cashews, pistachios), seeds (eg, sesame seeds, sunflower seeds, pumpkin seeds) and peanuts (including peanut butter).	Includes pulses, beans, legumes and foods primarily (>51%) from nuts or seeds.
Refined grains	g/day	Total intake of refined grains, defined as grains which have been milled to remove the bran and germ. Examples include white or polished rice, and products made with refined (white) flour, including white bread, pasta/noodles, cereals, crackers, and bakery products/desserts containing refined grains. This definition excludes corn products including corn flour and corn meal.	Includes corn products, soybeans, sweetened cakes and breads with grain as the main ingredient. May include whole grains.
Whole grains	g/day	Total intake of whole grains, defined as a food with ≥1.0 g of fibre per 10 g of carbohydrate, in which all components of the kernel (ie, bran, germ, and endosperm) are present in the same relative proportions as the intact grain. Examples include whole grain bread, brown rice, whole grain pasta, whole grain breakfast cereals, oats, rye, barley, millet, sorghum, and bulgur. This definition excludes corn products including corn flour, corn meal and popcorn.	Includes wholegrain breads, cereals, rice/pasta, bread and other products such as biscuits.
Total processed meats	g/day	Total intake of processed meat, defined as any meat (including poultry) that has been cured, smoked, dried or chemically preserved. Examples include bacon, salami, sausages, hot dogs and processed deli or luncheon meats. This definition excludes fish and eggs.	Includes sausages and unprocessed meats.
Unprocessed red meats	g/day	Total intake of unprocessed red meat, defined as beef, pork, lamb, mutton or game that has not been cured, smoked, dried or chemically preserved. This definition excludes poultry, fish and eggs.	Includes processed red meats, poultry, fish and organ meats.
Total seafoods	g/day	Total intake of fish and shellfish. Examples include salmon, tuna, trout, tilapia, shrimp, crab, oysters and cephalopods.	Includes salted fish, processed fish and other animal products.
Eggs	g/day	Total intake of eggs produced by poultry/birds, including chicken, goose, or duck eggs. This definition excludes fish eggs.	
Cheese	g/day	Total intake of cheese derived from the milk of livestock (eg, cows, buffalo, yak), including hard cheese (eg, cheddar, mozzarella, Swiss), soft cheese (eg, ricotta, cottage cheese, paneer) and processed cheese.	Includes yoghurt, milk products and cheese.
Yoghurt	g/day	Total intake of yoghurt and fermented milk, including reduced-fat and full-fat yoghurt.	Includes dairy curd, buttermilk, paneer, cheese and milk.
Sugar-sweetened beverages	g/day	Total sugar-sweetened beverage intake, defined as any beverage with added sugar having ≥50 kcal per eight ounces (236.5 g) serving, including commercial or homemade beverages, soft drinks, energy drinks, fruit drinks, punch, lemonade, and frescas. This definition excludes 100% fruit and vegetable juices and non-caloric artificially sweetened drinks.	Includes fruit and vegetable juices. May also include coffee, tea and milk.
Fruit juices	g/day	Total intake of 100% fruit juice, excluding sugar-sweetened fruit juice and vegetable juice.	Includes fruit juices, vegetable juices and sweetened juices.
Coffee	Cups/day (one cup=8 ounces)	Total coffee intake including caffeinated, decaffeinated, sweetened or unsweetened coffee.	Includes tea.
Tea	Cups/day (one cup=8 ounces)	Total green or black tea intake, including caffeinated, decaffeinated, sweetened or unsweetened tea. This definition excludes herbal tea.	Includes coffee.
Reduced fat milk	g/day	Total reduced-fat dairy milk intake, including non-fat, low-fat milk and skim milk. This definition excludes yoghurt, fermented milk and soy or plant-derived milk (eg, coconut milk, almond milk).	Includes sweetened reduced fat flavoured milk.
Whole fat milk	g/day	Total whole-fat dairy milk intake. This definition excludes yoghurt, fermented milk, and soy or other plant-derived milk (eg, coconut milk, almond milk).	Includes sweetened whole fat flavoured milk.
Total milk	g/day	Total intake of dairy milk including non-fat, low-fat, skim, and whole-fat milk. This definition excludes yoghurt, fermented milk and soy or other plant derived milk (eg, coconut milk, almond milk).	Includes yoghurt, dairy drinks, cheese and dairy products.
Total energy	Kcal/day	Total energy intake.	
Total carbohydrates	Per cent energy/day	Total carbohydrate intake.	
Total protein	g/day	Total protein intake from all sources.	
Animal protein	g/day	Total protein intake from animal sources.	
Plant protein	g/day	Total protein intake from plant sources.	
Saturated fat	Per cent energy/day	Total saturated fat intake from all sources (primarily meat and dairy products, and tropical oils).	
Monounsaturated fat	Per cent energy/day	Total monounsaturated fat intake from all sources.	
Total omega-6 fatty acids	Per cent energy/day	Total omega-6 fatty acid intake from all sources (primarily liquid vegetable oils, including soybean oil, corn oil and safflower oil), excluding dietary supplements.	Includes total polyunsaturated fat or linoleic acid.
Seafood omega-3 (n-3) fat	mg/day	Total dietary EPA+DHA (eicosapentaenoic acid +docosahexaenoic acid) intake, excluding dietary supplements.	Includes total dietary EPA+DPA+ DHA (eicosapentaenoic acid +docosahexaenoic acid+docosapentaenoic acid), long chain omega-3 only, excluding ALA (alpha-linolenic acid) and total seafood intake (fish and shellfish).
Plant omega-3 (n-3) fat	mg/day	Total dietary ALA (alpha-linolenic acid) intake, excluding dietary supplements.	Includes ALA (alpha-linolenic acid)+long chain omega-3 (EPA, DPA, DHA) (eicosapentaenoic acid, docosahexaenoic acid, docosapentaenoic acid)
Trans fatty acid	Per cent energy/day	Total trans fatty acid intake from all dietary sources (mainly partially hydrogenated vegetable oils, and ruminant products).	Includes calculated TFA (trans fatty acids) based on other kinds of measurements andpartially hydrogenated vegetable oil. Does not include TFA plasma measurements.
Dietary cholesterol	mg/day	Total dietary cholesterol from all sources.	
Dietary fibre	g/day	Total dietary fibre intake from all sources (fruits, vegetables, grains, legumes, pulses), defined as the carbohydrate polymers which are not hydrolyzed by the endogenous enzymes in the small intestine of human beings. Dietary fibre should optimally be quantified using the AOAC method of analysis.	
Added sugar	Per cent energy/day	Total intake of sugar added during the preparation or processing of foods and beverages. Examples include the sugar added in sugar-sweetened beverages, desserts, candy, breakfast cereals and sweetened milk. This definition excludes non-caloric sweeteners and sugar that naturally occur in foods, such as those in fruits, milk or milk products.	Includes all dietary sugar.
Calcium	mg/day	Total intake of calcium from all sources, excluding dietary supplements.	Includes intake from supplements in a population with relatively low supplement use.
Dietary sodium	mg/day	Total intake of sodium from all sources.	Includes urinary sodium.
Iodine	µg/day	Total intake of iodine from all sources, excluding dietary supplements.	Includes intake from supplements in a population with relatively low supplement use.
Iron	mg/day	Total intake of heme and non-heme iron from all sources, excluding dietary supplements.	Includes intake from supplements in a population with relatively low supplement use.
Magnesium	mg/day	Total intake of magnesium from all sources, excluding dietary supplements.	Includes intake from supplements in a population with relatively low supplement use.
Potassium	mg/day	Total intake of potassium from all sources, excluding dietary supplements.	
Selenium	µg/day	Total intake of selenium from all sources, excluding dietary supplements.	Includes intake from supplements in a population with relatively low supplement use.
Vitamin A with supplements	µg RAE/day (RAE=retinol activity equivalent)	Total intake of vitamin A (including retinol, retinal, retinoic acid, and retinyl esters) and provitamin A carotenoids from all sources, including dietary supplements.	
Vitamin A without supplements	µg RAE/day (RAE=retinol activity equivalent)	Total intake of vitamin A (including retinol, retinal, retinoic acid, and retinyl esters) and provitamin A carotenoids from all sources, excluding dietary supplements.	May include only retinol or carotenes.
Vitamin B_1_ (thiamine)	mg/day	Total intake of thiamin from all sources, excluding dietary supplements.	Includes intake from supplements in a population with relatively low supplement use.
Vitamin B_2_ (riboflavin)	mg/day	Total intake of vitamin B_2_ from all sources, excluding dietary supplements.	Includes intake from supplements in a population with relatively low supplement use.
Vitamin B_3_ (niacin)	mg/day	Total intake of niacin from all sources, excluding dietary supplements.	Includes intake from supplements in a population with relatively low supplement use.
Vitamin B_6_	mg/day	Total intake of vitamin B_6_ (including 2-methyl, 3-hydroxy, 5-hydroxymetrhyl pyridine derivatives that exhibit the nutritional activity of pyridoxine) from all sources, excluding dietary supplements.	Includes intake from supplements in a population with relatively low supplement use.
Vitamin B_9_ (folate)	µg/day DFE (DFE=dietary folate equivalent)	Total intake of folic acid from all sources, excluding dietary supplements.	Includes food fortification and supplements in a population with relatively low supplement use.
Vitamin B_12_	µg/day	Total dietary intake of cobalamins (including cyanocobalamin, hydroxocobalamin, aquocobalamin, sulfitocobalamin, etc) from all sources, excluding dietary supplements.	Includes intake from supplements in a population with relatively low supplement use.
Vitamin C	mg/day	Total intake of vitamin C from all sources, excluding dietary supplements.	Includes intake from supplements in a population with relatively low supplement use.
Vitamin D	µg/day	Total intake of vitamin D from dietary sources only, including vitamin D_2_, vitamin D_3_, and vitamin D provitamins and previtamins, excluding dietary supplements.	Includes intake from supplements in a population with relatively low supplement use.
Vitamin E	mg/day	Total intake of vitamin E tocopherols and tocotrienols from all sources, excluding dietary supplements.	Includes intake from supplements in a population with relatively low supplement use and alpha tocopherol.
Zinc	mg/day	Total intake of zinc from all sources, excluding dietary supplements.	Includes intake from supplements in a population with relatively low supplement use.

The foods capture nearly the entire diet, with the exceptions of poultry, dairy-based desserts, candy and sweeteners, and cakes, cookies and other baked goods, which may be collected in future iterations of the GDD.

AOAC, Association of Official Analytical Chemists; DFE, dietary folate equivalent; GDD, Global Dietary Database; RAE, retinol activity equivalent.

### Searches for published and publicly available dietary surveys

We systematically searched online global and regional databases including PubMed, Embase, and Web of Science, LILACS, African Index Medicus and the Southeast Asia Index Medicus. Search terms included: ‘nutrition’ OR ‘diet’ OR ‘food habits’ OR ‘nutrition surveys’ OR ‘diet surveys’ OR ‘food habits’[mesh] OR ‘diet’[mesh] OR ‘nutrition surveys’[mesh] OR ‘diet surveys’[mesh] AND (‘country of interest’). Additional search terms were applied to refine or expand each country search, as appropriate (online supplemental text S1, online supplemental table S3). Data searches encompassed 207 countries/territories, nested within seven world regions, with special focus on subregions previously identified as data-sparse (sub-Saharan Africa, Oceania, Southeast Asia, South Asia, Central Asia, Latin America/Caribbean, Middle East/North Africa).^10 14^ Because few identified publications reported dietary intake data according to comparable definitions or stratified by all relevant subgroups, we used published articles to identify data owner contacts. As done for GDD 2010,^10 24^ data owners were invited to join the GDD as a corresponding member (CM), which involved contributing their expertise and survey data through standardised electronic forms. GDD 2010 CMs were also invited to update previously submitted data with the new age group, dietary factor and other subgroup strata, as well as share any newly collected data. A detailed contact and communication algorithm was used to maximise responsiveness and participation (online supplemental text S2). Each CM registered their survey and its characteristics, completed a data sharing agreement, and uploaded the data as individual-level data (preferred) or stratum-level data based on standardised dietary factor and strata definitions.

10.1136/bmjgh-2020-003585.supp1 Supplementary data

Relevant publicly available surveys were identified using systematic database searches for health and nutrition surveys, as well as communication with our global CM network. For each potentially relevant public survey, data codebooks were screened for inclusion, and eligible surveys downloaded with prioritisation of data-sparse regions and nations as well as large (populous) nations.

### Survey screening and inclusion

Identified published articles were screened by title and abstract and, for all potentially relevant articles, screened as full text by a single reviewer. A random subset of articles from each database and geographic region were screened by a second reviewer to ensure consistency and accuracy. When published reports did not contain the necessary data format (most often), data owners were invited to become CMs and share their data. Datasets and survey documentation submitted by CMs and those from public surveys were reviewed again by a third reviewer to ensure survey inclusion criteria were met. We prioritised nationally or subnationally representative surveys whenever available. When no such surveys were identified for a nation, we allowed community-level surveys, and then household-level surveys, if these were felt to be representative of the community; that is, such surveys were excluded if focused on special populations (eg, people with specific disease conditions) or cohorts (eg, people of a certain profession or dietary pattern).

### Data retrieval and assessment

Standardised protocols were used to identify, extract and analyse data in a systematic and comparable manner. For CM-provided surveys, survey characteristics were retrieved using a standardised electronic form, including data on survey name, country, years performed, sampling methodology, response rate, national representativeness, level of data collection (individual or household level), dietary assessment method and validation, sample size, population demographics (age, sex, education, urban/rural residence, pregnancy/lactation status), and definitions and measurement units of dietary factors. Individual-level microdata were retrieved as SAS, STATA, SPSS v.25 or Microsoft Excel files. Aggregate stratum-level dietary intake data were collected using standardised electronic spreadsheets, including data on stratum sample size and means, SD, and 10th, 25th, 50th, 75th and 90th percentiles of intake for each dietary factor, jointly stratified by age, sex, education and urban/rural strata, as available. Data from publicly available surveys were retrieved using a similar standardised electronic spreadsheet as for CM surveys. Random double checks of data retrievals were performed to ensure correct extraction of publicly available surveys. When the same study collected information across multiple countries, data for each country were separated and counted as a separate survey for reporting purposes.

### Data standardisation

Standardisation included data quality assessment, standardised categorisation of foods, beverages and nutrients and their units, aggregation by subgroup strata, energy-adjustment and compilation into a relational database. Each dietary variable was characterised according to a standard definition and units (table 1). Surveys with varying definitions were classified using defined secondary definitions. When multiple days of dietary intakes were collected (eg, diet recalls or records), these were averaged for each individual. Semi-quantitative instruments (eg, FFQs based on a single specified portion size) and short standardised questionnaires (eg, DHS surveys) were converted to standard serving sizes for each frequency category. Household-level data were converted to individual-level intakes within each household using Adult Male Equivalents,^25^ which accounts for the household composition and differing energy intakes by age and sex of household members. Based on national estimated average requirements^26–28^ and observed population intakes,^29^ all intakes were adjusted to 700 kcal/day for ages 0 to <1 years, 1000 kcal/day for ages 1 to <2 years, 1300 kcal/day for ages 2–5 years, 1700 kcal/day for ages 6–10 years, 2000 kcal/day for ages 11–74 years and 1700 kcal/day for ages 75+ years (online supplemental text S3). Individual-level microdata were aggregated into subgroups jointly stratified by age (0–5, 6–11 and 12–23 months; and 2–4, 5–10, 11–14, 15–19, 20–24, 25–29, 30–34, 35–39, 40–44, 45–49, 50–54, 55–59, 60–64, 65–69, 70–74, 75–79, 80–84, 85–89, 90–94 and 95+years), sex, education (≤6 years of education, 6.01–12 years or ≥12.01 years; and for children, head of household’s educational attainment), urban/rural residence, and pregnancy/lactation status, as available. Urban versus rural residence were defined according to each survey’s established definition, due to absence of any single global definition of these factors as well as logistical challenges in aiming to revise each survey’s existing definitions. Education was selected as the most widely available and standardised metric of socioeconomic status, as compared with income or wealth indices which are not always reported similarly or accurately across countries.

### Quality control and data management

Data integrity and quality were assessed at each step during survey collection, processing, standardisation and analyses. Duplicate reviews were performed of recorded survey characteristics, demographic variables, dietary definition classifications and unit conversions. To assess for outliers and validity (errors) in reported intakes, plausibility thresholds were defined for each dietary factor, both at the individual level and stratum (eg, group mean) level, based on dietary reference intakes, tolerable upper limits, toxicity ranges and existing regional data on mean intakes in populations (online supplemental tables S9–S14). Any value identified as potentially implausible was reviewed for extraction errors, followed by direct correspondence with the CM or public survey data owners, to detect and correct potential errors. Data remaining implausible after such steps were excluded from final datasets. Results for each dietary factor were further graphed and visually inspected by country, age, sex, dietary assessment method, representativeness and time period, reviewing survey result plausibility and consistency within and across countries.

Data analyses were performed using SAS V.9.4 (SAS Institute), Stata V.14.0 (StataCorp) and RStudio V.1.1.453 (RStudio, Massachusetts, USA). Data files were organised using Microsoft Access 2010 relational database (Microsoft, Redmond, Washington, USA), linking survey characteristics, data owner institutional information, and survey processing and standardisation details. SQL queries were developed to deduplicate data, summarise survey characteristics and calculate survey quality scores.

### Modelling and imputation

In addition to identifying, collecting, standardising and disseminating survey-level data, the GDD 2017 uses advanced imputation modelling to account for differences in survey design, representativeness, dietary assessment methods and dietary factor definitions, as well uncertainty in dietary estimates and missingness, to estimate stratum-specific mean dietary intakes jointly stratified by age, sex, education and urban/rural residence (N=240 strata per country year), for each of 188 countries/territories per year between 1990 and 2018. Our modelling methods for GDD 2010 have been reported,^11 15 30 31^ and the updated modelling methods and findings for GDD 2017 will be the focus of a forthcoming paper.

### Patient and public involvement statement

There was not public involvement in the study; we used publicly available or privately held data for the analysis.

## Results

### Survey identification, retrieval and inclusion

**Figure 1:**
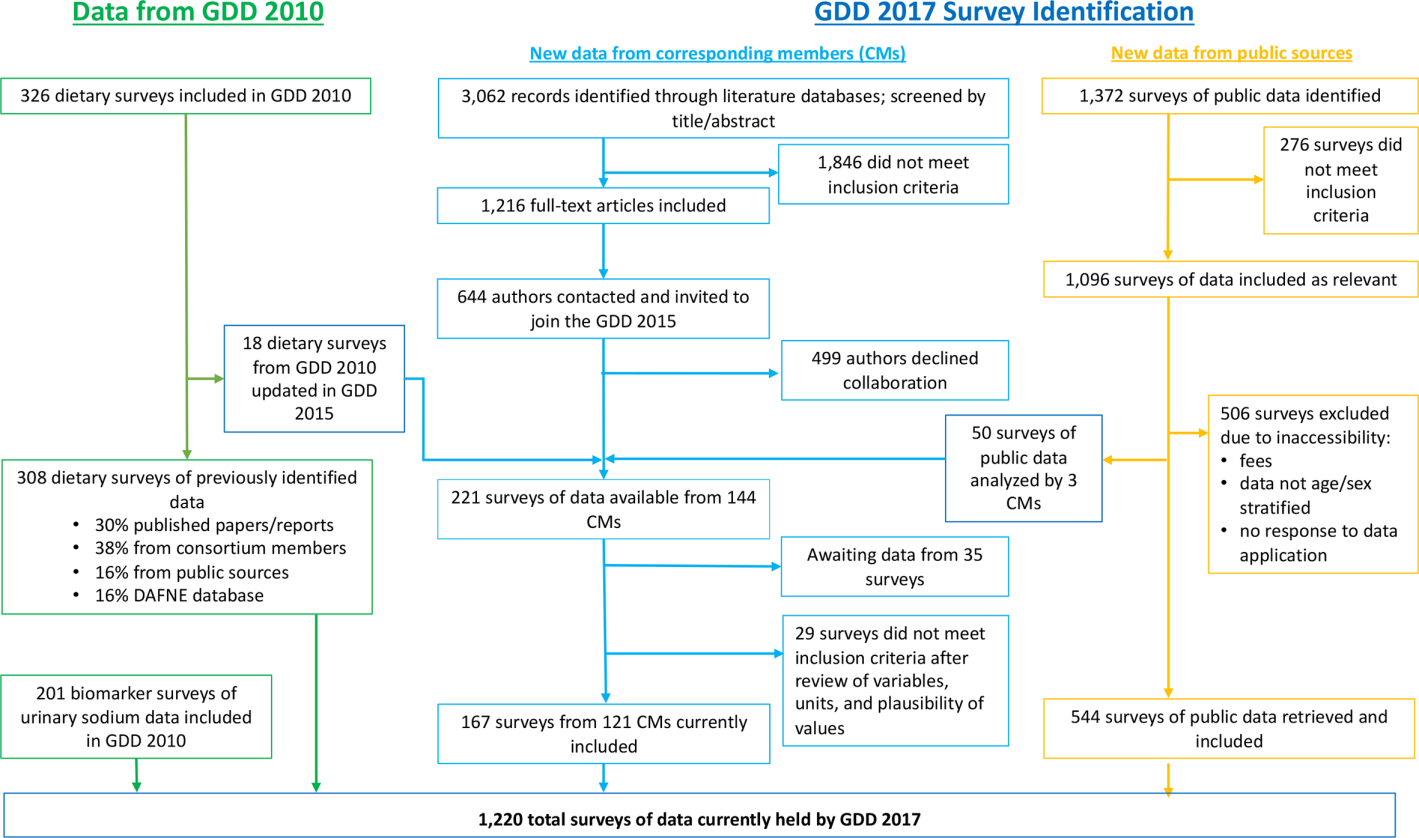
Flow chart depicting the Global Dietary Database (GDD) process for data identification and retrieval. GDD 2010 refers to the iteration conducted between 2008 and 2011, GDD 2017 new data from corresponding members (CMs) refers to surveys contributed by a data owner during the 2014–2020 iteration, GDD 2017 new data from public sources refers to surveys retrieved from publicly available databases during the 2014–2020 iteration.

Our new systematic searches identified 3062 potentially relevant published abstracts (figure 1). Of these, 221 eligible surveys were identified from 144 CMs, of which 167 surveys (76%) were retrieved from CMs, analysed, cleaned and standardised by the time of database lock on 31 March 2020. Among 1372 potentially relevant publicly available surveys, 544 eligible surveys were retrieved, analysed, cleaned and standardised. Including the prior surveys identified in GDD 2010, the final GDD 2017 dataset included 1220 surveys.

### Characteristics of dietary surveys

The 1220 surveys were from 188 countries/territories, together representing 99.0% of the global population in 2015 (table 2). About 7 in 10 (70.8%) were retrieved from public sources, and the remainder (29.2%) from CMs. 72.1% were nationally representative (representing 99.6% of the global population in 2015), 17.0% were subnationally representative (81.6% of the global population in 2015), and 10.9% were community level (70.2% of the global population in 2015) (more than one type of survey could be included for individual nations). The great majority of surveys (92.5%) were collected at the individual level. About one-third (36.1%) were collected before 2000, and the rest (63.9%) in 2000 or later. Most (58.6%) were retrieved by the GDD as individual-level microdata, and others as aggregated, standardised, stratum-level intakes.

**Table 2:** Characteristics of 1220 dietary surveys in the GDD 2017

Survey characteristics	Overall	Public surveys*	Private surveys*
Surveys included†, n	1220	864	356
No of countries represented, n	188	174	128
Per cent of the global population represented in 2015, %	99.0	98.1	87.4
Demographic characteristics reported by surveys‡			
Children/adolescents (ages 0–19 years), %	73.9	76.4	68.0
Age 0–0.49	26.3	35.1	5.1
Age 0.5–0.99	26.9	35.4	6.2
Age 1–1.9	29.3	34.3	17.0
Age 2–5.9	32.5	35.6	25.0
Age 6–10	32.0	33.6	28.1
Age 11–14	49.8	57.3	31.7
Age 15–19	61.2	64.6	53.1
Adults (ages 20+ years), %	64.5	57.2	82.3
Age 20–44	62.5	56.4	77.2
Age 45–69	61.7	56.3	75.0
Age 70+	32.8	25.6	50.3
Level of educational attainment, %	30.2	24.7	43.5
Urban versus rural residence, %			
Both urban and rural	52.2	62.3	27.8
Urban only	4.7	0.7	14.3
Rural only	1.4	0.0	4.8
Information not available	41.7	37.0	53.1
Pregnancy/lactation status of women, %	11.2	15.2	1.7
Year of data collection, %			
1980–1999	36.1	42.0	21.6
2000–2015	63.9	58.0	78.4
Representativeness			
National, No surveys (% of total) No countries (% of global population represented)	880 (72.1) 185 (99.6)	664 (76.9) 172 (99.6)	216 (60.7) 95 (84.7)
Subnational, No surveys (% of total) No countries (% of global population represented)	207 (17.0) 75 (81.6)	146 (16.9) 45 (67.8)	61 (17.1) 46 (46.2)
Community, No surveys (% of total) No countries (% of global population represented)	133 (10.9) 48 (70.2)	54 (6.3) 19 (35.1)	79 (22.2) 36 (48.2)
Response rate, %			
60%–100%	37.8	24.8	69.4
20%–59%	3.2	1.2	8.1
<20%	5.7	8.0	0.0
Information not available	53.4	66.1	22.5
Sampling methodology, %			
Probability sampling, with survey weights	38.4	39.5	36.0
Probability sampling, no survey weights	32.7	32.9	32.3
Non-probability sampling	4.2	0.7	12.6
Information not available	24.7	27.0	19.0
Median (5th, 95th percentile) no of GDD dietary food groups per survey§	6.0 (1.0, 30.0)	3.0 (1.0, 14.0)	11.0 (2.0, 47.0)
Dietary assessment method			
Single or multiple recalls/records			
No surveys, (% of total)	286 (23.4))	96 (11.1)	190 (53.4)
No countries, (% global population represented)	68 (78.0)	90 (40.4)	64 (84.7)
Food frequency questionnaire			
No surveys, (% of total)	503 (41.2)	346 (40.0)	157 (44.1)
No countries, (% of global population represented)	164 (94.3)	141 (89.5)	89 (57.2)
DHS questionnaire			
No surveys, (% of total)	193 (15.8)	190 (22.0)	3 (0.8)
No countries, (% of global population represented)	70 (53.9)	69 (53.4)	2 (1.7)
Biomarker (urine sodium, haemoglobin)			
No surveys, (% of total)	160 (13.1)	154 (17.8)	6 (1.7)
No countries, (% of global population represented)	60 (70.7)	59 (71.3)	5 (2.5)
Household survey			
No surveys, (% of total)	78 (6.4)	78 (9.0)	0 (0.0)
No countries, (% of global population represented)	27 (15.1)	27 (15.3)	0 (0.0)
Data type¶, %			
Individual-level microdata	58.6	65.6	41.6
Aggregated stratum-level distributions	41.4	34.4	58.4
Level of data collection, %			
Individual level	92.5	89.8	99.2
Household level	5.7	7.8	0.8
Information not available	1.7	2.4	0.0

*Public surveys are those retrieved from publicly available databases; private surveys are non-publicly available surveys that are contributed by a data owner (corresponding member);.

†Each survey count represents a country-specific survey year. When data collection for a single survey was performed over multiple years, the median survey year was used (or first year if 2 years).

‡Because data on children/adolescents (0–19 years), urban/rural residence, education, pregnancy/lactation and response rate were not collected in GDD 2010 (41.7% of total surveys), these percentages may underestimate available data in these surveys. Values are shown for surveys including data on that subgroup and may sum to greater than 100% because a survey can include multiple subgroups.

§Based on the food groups collected in GDD 2010 (up to 21, 41.7% of surveys) and GDD 2017 (up to 54, 58.3% of surveys), not including biomarker surveys.

¶Individual-level microdata represent individual-level data in possession of the GDD. Aggregated stratum-level distributions are based on individual-level data aggregated by data owners in standardised subgroups jointly stratified by age, sex, education and urban/rural residence, and pregnancy/lactation status, as available; and provided to the GDD including stratum-specific means, SD and percentiles of intake. Nearly all (94.2%) surveys collected in 2014–2020 (GDD 2017 round of data collection) were individual-level microdata.

DHS, Demographic Health Survey; GDD, Global Dietary Database.

Dietary instruments included FFQs (41.2% of surveys; representing 94.3% of the global population in 2015), 24-hour recalls (23.4%; 78.0%), DHS questionnaires (15.8%; 53.9%), biomarkers (13.1%; 70.7%) and household surveys (6.4%; 15.1%) (more than one type of survey could be included for individual nations) (table 2). Most surveys included data on children and adolescents (age 0–19 years; 73.9%); about two-thirds of surveys (64.5%) included data on adults (age 20+ years). More than half (52.2%) included data that specified urban or rural residence of individuals, including 4.7% urban only and 1.4% rural only. Data on education level or pregnancy/lactation status of participants was available in 30.2% and 11.2% of surveys, respectively.

Compared with CM-provided surveys, public surveys were more often nationally representative (76.9% vs 60.7%), conducted before 2000 (42.0% vs 21.6%) and available as individual-level microdata (65.6% vs 41.6%), and more likely to be household level (7.8% vs 0.8%) (table 2). Many more public vs CM surveys used DHS questionnaires (22.0% vs 0.8%) or biomarkers (17.8% vs 1.7%), and many fewer used 24-hour recalls (11.1% vs 53.4%). Excluding biomarker studies, the median (5th, 95th percentile) number of dietary factors per survey was 6.0 (1.0, 30.0), and was lower in public surveys (3.0; 1.0, 14.0) vs CM surveys (11.0; 2.0, 47.0).

### Data availability by world region

The largest number of surveys were from high-income countries (N=386), followed by sub-Saharan Africa (N=210), Asia (N=180), Former Soviet Union (N=158), Latin America/Caribbean (N=135), Middle East/North Africa (N=95) and South Asia (N=56) (table 3). In all regions except South Asia, most surveys were nationally representative. The great majority of surveys used either 24-hour recalls or FFQs in all world regions except sub-Saharan Africa, where nearly half of all available surveys were DHS questionnaires (which assess only children age 0–5 years and their mothers and women of reproductive age, 15–49 years).

**Table 3:** Characteristics of 1220 dietary surveys in the GDD 2017, by world region*

	World	Asia	Former Soviet Union	High-income countries	Latin America/ Caribbean	Middle East/North Africa	South Asia	Sub-Saharan Africa
Surveys included, n	1220	180	158	386	135	95	56	210
No of countries with surveys (% of world population in 2015)	188 (99.0)	30 (30.6)	28 (5.5)	25 (10.9)	34 (8.3)	20 (6.9)	7 (23.3)	44 (13.3)
Ages included†								
Children (0–19 years)	902 (73.9)	108 (60.0)	142 (89.9)	254 (65.8)	109 (80.7)	74 (77.9)	45 (80.4)	170 (81.0)
Adults (20+ years)	787 (64.5)	143 (79.4)	94 (59.5)	254 (65.8)	64 (47.4)	56 (58.9)	38 (67.9)	138 (65.7)
Representativeness								
National	880 (72.1)	108 (60.0)	149 (94.3)	263 (68.1)	100 (74.1)	70 (73.7)	28 (46.4)	162 (77.1)
Subnational	207 (17.0)	39 (21.7)	7 (4.4)	94 (24.4)	17 (12.6)	13 (13.7)	10 (17.9)	27 (12.9)
Community	133 (10.9)	33 (18.3)	2 (1.3)	29 (7.5)	18 (13.3)	12 (12.6)	18 (32.1)	21 (10.0)
Median (5th, 95th percentile) number of GDD dietary food groups per survey‡	6.0 (1.0, 30.0)	5.5 (1.0, 31.4)	6.0 (2.0, 17.0)	6.0 (1.0, 31.9)	5.0 (2.0, 36.9)	3.0 (1.0, 47.0)	10.0 (2.0, 26.7)	7.0 (2.0, 14.0)
Dietary assessment method								
Single or multiple diet recalls/records								
No surveys	286 (23.4)	69 (38.3)	25 (15.8)	125 (32.4)	18 (13.3)	18 (18.9)	16 (28.6)	15 (7.1)
No countries, % regional population represented	68 (78.0)	12 (95.9)	8 (19.1)	21 (98.7)	7 (59.9)	7 (42.5)	4 (89.9)	15 (39.9)
Food Frequency Questionnaire								
No surveys, %	503 (41.2)	71 (39.4)	86 (54.4)	132 (34.2)	65 (48.1)	61 (64.2)	24 (42.9)	64 (30.5)
No countries, % regional population represented	164 (94.3)	28 (98.9)	23 (87.0)	22 (96.2)	32 (97.6)	19 (99.7)	7 (100.0)	33 (70.2)
DHS questionnaire								
No surveys, %	193 (15.8)	14 (7.8)	10 (6.3)	0 (0.0)	40 (29.6)	14 (14.7)	14 (25.0)	101 (48.1)
No countries, % regional population represented	70 (53.9)	5 (21.0)	8 (30.3)	0 (0.0)	11 (79.7)	4 (41.4)	5 (98.7)	37 (95.8)
Biomarker								
No surveys, %	160 (13.1)	26 (14.4)	7 (4.4)	82 (21.2)	11 (8.1)	2 (2.1)	2 (3.6)	30 (14.3)
No countries, % regional population represented	60 (70.7)	7 (85.8)	6 (50.2)	18 (95.4)	9 (39.2)	2 (31.2)	1 (76.5)	17 (54.3)
Household budget survey								
No surveys, %	78 (6.4)	0 (0.0)	30 (19.0)	47 (12.2)	1 (0.7)	0 (0.0)	0 (0.0)	0 (0.0)
No countries, % regional population represented	27 (15.1)	0 (0.0)	9 (52.9)	17 (84.5)	1 (33.8)	0 (0.0)	0 (0.0)	0 (0.0)
Data type								
Individual-level microdata	715 (58.6)	74 (41.1)	99 (62.6)	146 (37.8)	114 (84.4)	73 (76.8)	45 (80.4)	164 (78.1)
Aggregated stratum-level distributions	505 (41.4)	106 (58.9)	59 (37.3)	240 (62.2)	21 (15.6)	22 (23.2)	11 (19.6)	46 (21.9)
Data source								
Contributed by data owner	356 (29.4)	90 (50.0)	38 (24.1)	85 (22.0)	42 (31.1)	41 (43.2)	27 (48.2)	33 (15.7)
Retrieved from public source	864 (70.6)	90 (50.0)	120 (75.9)	301 (78.0)	93 (68.9)	54 (56.8)	29 (51.8)	177 (84.3)

Data are survey counts (%).

*Countries in each world region are: Asia: Brunei Darussalam, Cambodia, China, Fiji, Hong Kong, Indonesia, Japan, Kiribati, Lao People’s Democratic Republic, Macau, Malaysia, Marshall Islands, Micronesia, Myanmar, North Korea, Papua New Guinea, Philippines, Republic of Korea, Samoa, Singapore, Solomon Islands, Taiwan, Thailand, Timor-Leste, Tonga, Vanuatu, Viet Nam. Former Soviet Union: Albania, Armenia, Azerbaijan, Belarus, Bosnia and Herzegovina, Bulgaria, Croatia, Czech Republic, Estonia, Georgia, Hungary, Kazakhstan, Kyrgyzstan, Latvia, Lithuania, Mongolia, Montenegro, Poland, Republic of Moldova, Romania, Russian Federation, Serbia, Slovakia, Slovenia, Tajikistan, The former Yugoslav Republic of Macedonia, Turkmenistan, Ukraine, Uzbekistan. High-income countries: Andorra, Australia, Austria, Belgium, Canada, Cyprus, Denmark, Finland, France, Germany, Greece, Iceland, Ireland, Italy, Luxembourg, Malta, Netherlands, New Zealand, Norway, Portugal, Saint Pierre and Miquelon, Spain, Sweden, Switzerland, UK, USA. Latin America/Caribbean: Antigua and Barbuda, Argentina, Bahamas, Barbados, Belize, Bolivia, Brazil, Chile, Colombia, Costa Rica, Cuba, Dominica, Dominican Republic, Ecuador, El Salvador, Grenada, Guatemala, Guyana, Haiti, Honduras, Jamaica, Mexico, Montserrat, Nicaragua, Panama, Paraguay, Peru, Saint Lucia, Saint Vincent and the Grenadines, Suriname, Trinidad and Tobago, Uruguay, Venezuela. Middle East/North Africa: Algeria, Bahrain, Egypt, Iran (Islamic Republic of), Iraq, Israel, Jordan, Kuwait, Lebanon, Libyan Arab Jamahiriya, Morocco, Occupied Palestinian Territory, Oman, Qatar, Saudi Arabia, Syrian Arab Republic, Tunisia, Turkey, United Arab Emirates, Yemen. South Asia: Afghanistan, Bangladesh, Bhutan, India, Maldives, Nepal, Pakistan, Réunion, Sri Lanka. Sub-Saharan Africa: Angola, Benin, Botswana, Burkina Faso, Burundi, Cameroon, Cape Verde, Central African Republic, Chad, Comoros, Congo, Cote d’Ivoire, Democratic Republic of Congo, Djibouti, Equatorial Guinea, Eritrea, Ethiopia, Gabon, Gambia, Ghana, Guinea, Guinea-Bissau, Kenya, Lesotho, Liberia, Madagascar, Malawi, Mali, Mauritania, Mauritius, Mozambique, Namibia, Niger, Nigeria, Rwanda, Sao Tome and Principe, Senegal, Seychelles, Sierra Leone, Somalia, South Africa, South Sudan, Sudan, Swaziland, Togo, Uganda, United Republic of Tanzania, Zambia, Zimbabwe.

†Because data on children were not collected in GDD 2010 (41.7% of total surveys), these percentages may underestimate available data in these surveys. Values are shown for surveys including data on that age group and may sum to greater than 100% because a survey can include both children and adults.

‡Based on the food groups collected in the GDD 2010 (up to 21, 41.7% of surveys)) and GDD 2017 (up to 54, 58.3% of surveys)), not including biomarker surveys.

DHS, Demographic Health Survey; GDD, Global Dietary Database.

Most surveys from high-income countries and Asia were available as aggregated stratum-level distributions; and in other world regions, as individual-level microdata. The highest number of dietary factors per survey was in South Asia ((median; 5th, 95th percentile) 10.0; 2.0, 26.7); and lowest, in the Middle East/Northern Africa (3.0; 1.0, 47.0).

### Data availability by nation

**Figure 2:**
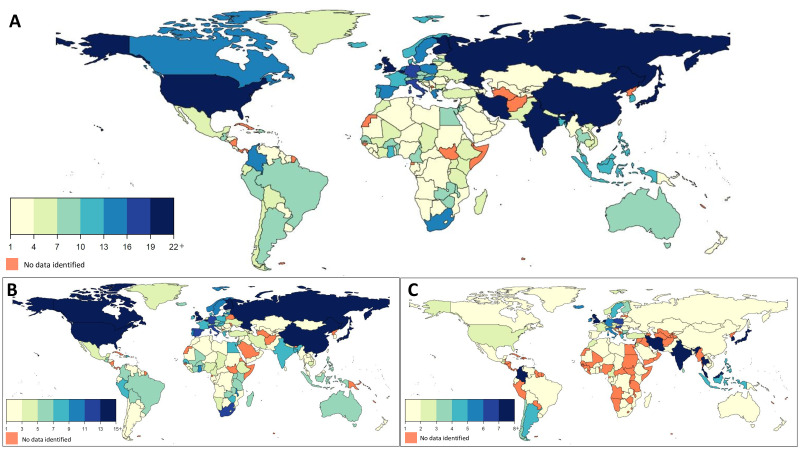
Geographical density of the number of dietary surveys in the GDD 2017 by country (A), including publicly available surveys (B) and non-public surveys submitted by data owners (corresponding members) (C). GDD, Global Dietary Database.

By country, the USA, Japan, Great Britain, Finland and China had the largest number of surveys (N>25 surveys each) (figure 2). Many countries in sub-Saharan Africa, Latin America/Caribbean, Middle East/North Africa, Former Soviet Union, and Asia had less than five surveys, with notable exceptions being China and Japan (mentioned above), Russia (N=22), Iran (N=20), Hungary (N=16), Poland (N=16) and Colombia (N=15) (figure 2). Eligible surveys were not identified for 19 of 207 world countries (Afghanistan, Andorra, Bermuda, Cuba, Democratic People’s Republic of Korea, Djibouti, Equatorial Guinea, French Polynesia, Guadeloupe, Guinea-Bissau, Hong Kong, Macau, Martinique, Netherlands Antilles, Nicaragua, Palau, Réunion, Somalia, Turkmenistan), together representing 1% of the world population in 2015.

### Data availability by dietary factor

**Figure 3:**
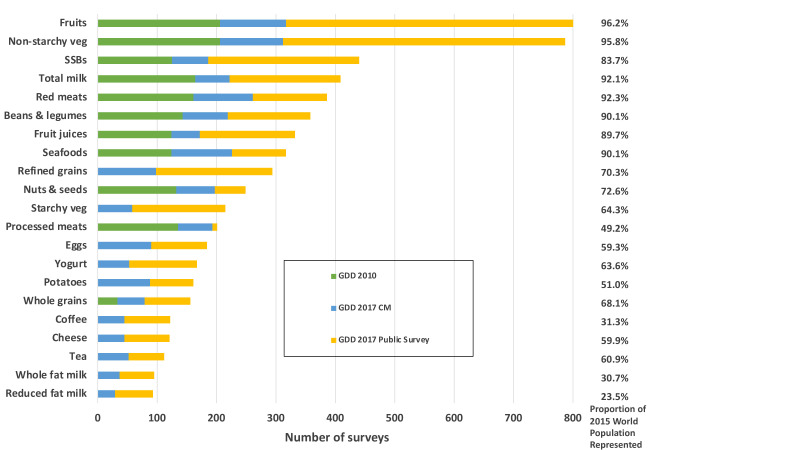
Number of surveys with dietary intake data by dietary factor and source in the GDD 2017. GDD 2010 refers to the iteration conducted between 2008 and 2011, GDD 2017 CM refers to surveys contributed by a data owner during the 2014–2020 iteration, GDD 2017 Public Survey refers to surveys retrieved from publicly available databases during the 2014–2020 iteration. CM, corresponding member; GDD, Global Dietary Database; SSB, sugar-sweetened beverage; veg, vegetables.

**Figure 4:**
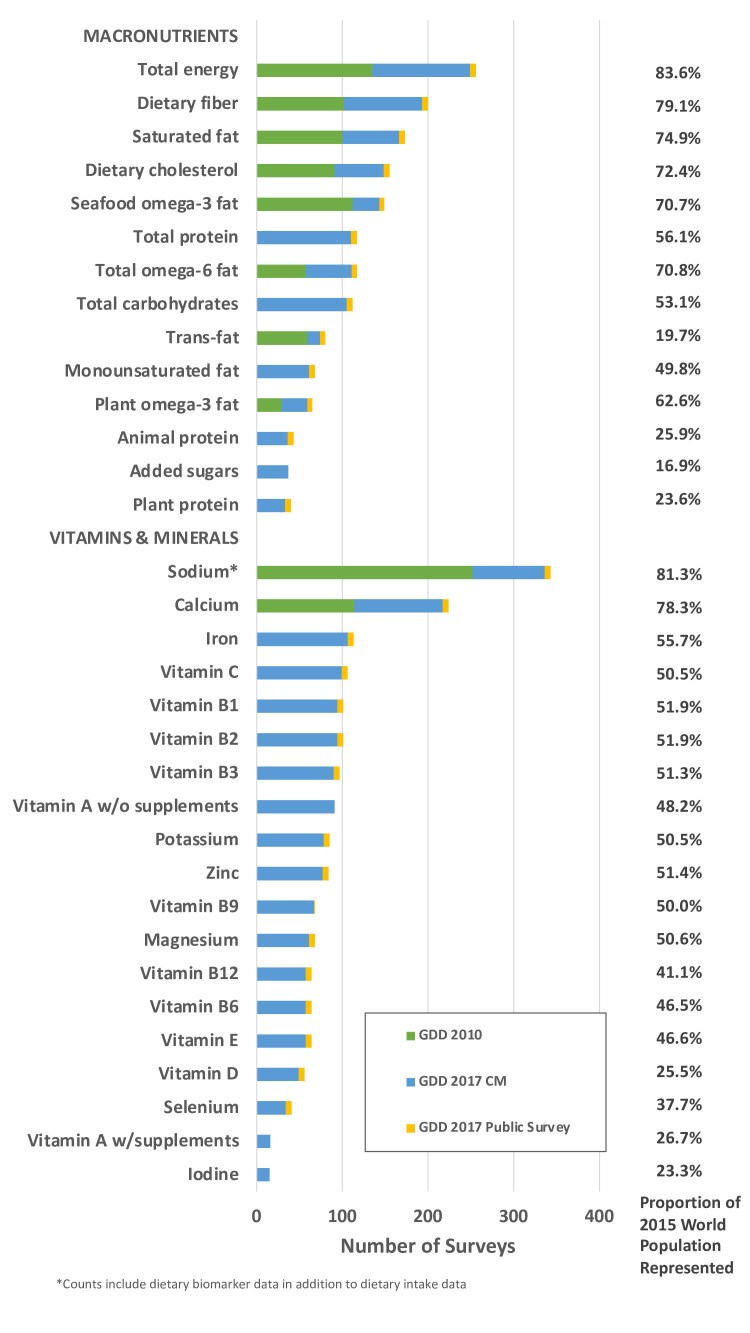
Number of surveys with dietary intake data on nutrients in the GDD 2017. GDD 2010 refers to the iteration conducted between 2008 and 2011, GDD 2017 CM refers to surveys contributed by a data owner during the 2014–2020 iteration, GDD 2017 Public Survey refers to surveys retrieved from publicly available databases during the 2014–2020 iteration. CM, corresponding member; GDD, Global Dietary Database; w/supplements, with supplements; w/o, without.

The most frequently evaluated factors were fruits (N=803 surveys, together representing 96.2% of the global population in 2015), non-starchy vegetables (N=787, 95.8%), sugar-sweetened beverages (N=440, 83.7%), total milk (N=413, 92.1%), unprocessed red meat (N=386, 92.3%), and beans/legumes (N=358, 90.1%) (figure 3). Fewer than 150 surveys were identified for coffee (N=122, 31.3%), cheese (N=121, 59.9%), tea (N=112, 60.9%), whole fat milk (N=95, 30.7%) and reduced fat milk (N=93, 23.5%), although these surveys still represented meaningful proportions of the global population. Among macronutrients, dietary fibre (N=200, 79.1%), saturated fat (N=173, 74.9%), and dietary cholesterol (N=155, 72.4%) had the largest number of surveys. Vitamins and minerals with the greatest number of surveys included sodium (N=343, 81.3%), calcium (N=224, 78.3%), iron (N=113, 55.7%) and vitamin C (N=106, 50.5%) (figure 4). Fewest surveys were identified for iodine, vitamin A, plant protein, selenium, added sugar and animal protein (N<45 surveys each). Notably, with the exception of sodium and calcium, ≥91% of all surveys including information on vitamins, or minerals were obtained from non-public (CM) sources.

## Discussion

In this systematic search for individual-level dietary intakes globally, we identified, retrieved and standardised 1220 surveys from 188 countries, together representing 99.0% of the world’s population in 2015. Most were nationally representative and collected at the individual-level using an FFQ or 24-hour recall. A broad range of ages were represented, from birth to late in life; more than half (52.2%) included information on urban or rural residence; and about one-third (30.2%) on educational attainment. Nearly one-third of surveys (29.2%) were not publicly available, retrieved directly from a global network of CMs; and these were more likely to be recent, use 24-hour recalls, and be the primary source of information globally on intakes of vitamins, minerals, and other micronutrients. In sum, this GDD 2017 dataset represents the most comprehensive collection of individual-level dietary intake data worldwide.

Despite extensive searches, we found fewer surveys from South Asia, as well as fewer surveys using higher-quality dietary instruments (eg, 24-hour recalls, FFQs) from sub-Saharan Africa. Surveys in the latter region were most often DHS questionnaires, which collect qualitative information (yes/no) on intakes in the preceding day of a limited number of foods and beverages for infants, children <5 years old, their mothers and women of reproductive age. Unfortunately, countries in these regions have also been more likely to suffer prolonged conflict and economic shocks impacting food security and nutrition,^32^ rendering even greater the challenges as well as importance of collecting robust nutrition surveillance data.

Less than one-third of surveys each provided data on infants or children younger than 10 years, older adults (age 70+ years), or by pregnancy/lactation status in women. Publicly available (especially DHS) surveys were more likely to report data on young children and pregnant/lactating women, while nonpublic surveys were more likely to report data on older adults. Because nutritional requirements are especially sensitive in these subgroups, our findings demonstrate the global need for more nationally representative high-quality surveys, using 24-hour recalls or FFQs, in these special populations. In addition, while South Asia comprises about a quarter of the world’s population and has the highest global prevalence of stunting and wasting,^33^ our results demonstrate the fewest number of dietary surveys with data on children (age 0–19 years) in this region. These novel findings highlight specific key gaps in dietary surveillance in this region.

Among different dietary factors, the availability of surveys varied substantially. Generally, more surveys collected data on intakes of foods, especially fruits and vegetables; and fewer surveys estimated nutrient intakes. Importantly, relatively few public surveys reported on vitamins and minerals relevant to MCH such as iron, vitamin A, zinc, iodine, folate and vitamin B_12_, among others. We found many public surveys used dietary instruments which estimate only a portion of the diet (eg, DHS questionnaires) and thus cannot estimate nutrient intakes; or included broader food assessments but without reliable and updated national food composition databases or food composition tables to estimate nutrients. This was identified to be particularly problematic in sub-Saharan Africa and Latin America/Caribbean, emphasising need for increased collection of individual level, nationally representative dietary data using 24-hour recalls or FFQs, together with creation or updating of food composition data, in these regions.

While 24-hour recalls are considered the best standard for assessing national and stratum-specific mean dietary intakes, their collection is more time and resource intensive than for FFQs, which also may better assess habitual intakes among each individual participant.^34 35^ Consistent with this, more global surveys used FFQs than 24-hour recalls. Both of these instruments are more valid than short dietary questionnaires, which collect far less detailed data on a handful of foods and beverages, creating more measurement error and far less coverage of the whole diet.^36^ However, short dietary questionnaires are easier and less expensive to administer, consistent with our findings that they are most common in low-resource nations. The GDD 2017 results highlight the unfortunate irony that micronutrient information is least available and valid where it matters most.

For specific factors, we identified and collected biomarker data but valid biomarkers are not available for most dietary factors.^37^ We did not use household-level data except where individual-level data were not available, given their significant limitations in assessing dietary intakes.^8^

Many prior efforts to estimate dietary intakes globally have used FAO FBS as primary data inputs.^12 38^ While FAO data represent powerful and useful annual estimates of national per capita availability of food commodities, they are not intended to capture and are poor representations of dietary intake.^39^ For example, they do not capture well, unreported food waste, local food production and especially subnational heterogeneity in dietary habits among different population subgroups.^6 40^ The Global Burden of Diseases Study is another study that estimates global diet. While the 2010 and 2013 cycles of the Global Burden of Diseases Study collaborated with the GDD 2010 for their global dietary estimates, the Global Burden of Diseases Study subsequently internalised their processes for estimating diets. Based on available publications, that study now uses FAO FBS estimates, national product sales data and household budget surveys as primary data inputs, adjusting using a single global regression against individual-level diet surveys from 67 countries.^2 41^ Because the relationship between national food availability and individual-level dietary intakes is known to vary significantly and jointly by age, sex, world region and other factors,^39^ such methods will not sufficiently capture the heterogeneity in national and subnational intakes across diverse countries. Compared with GDD 2017 which includes data on 54 dietary factors, the Global Burden of Diseases Study currently reports on 8 foods, 1 beverage and 6 nutrients. We and others have collaborated with Gallup, Inc., in their planning for potential standardised polling on dietary intakes.^42^ Such data, mostly likely based on short dietary questionnaires, will not capture the full diet nor estimate nutrients but will complement DHS data and provide useful new inputs to the GDD global dietary modelling efforts.

Overall, while gaps and heterogeneity in data sources are evident, the GDD 2017 represents, to our knowledge, the most comprehensive and updated data on global dietary intakes. To maximise its benefits as a public resource, the GDD 2017 is now available for free public download at http://www.globaldietarydatabase.org. Survey-level information and original data download weblinks are provided for all public surveys; and survey-level microdata or stratum-level aggregate data, as available, are provided for direct download for all non-public (CM) surveys granted consent for public sharing by the data owners (currently 81.9%). Importantly, the full modelled GDD 2017 data, which will leverage all surveys as primary data inputs together with survey- and country-level covariates to estimate the mean intakes of all 54 food and nutrients within each of 240 subnational subgroups in 188 nations by year between 1990 and 2018, will also be available for free public download when finalised (estimated Spring of 2021).^9^ The GDD is also collaborating with the FAO/WHO Global Individual Food consumption data Tool (FAO/WHO GIFT) project^43^ and European Food Safety Authority to jointly facilitate harmonisation of dietary datasets on a global scale, public dissemination of methods and dietary datasets, and global collaboration and capacity development with dietary data owners worldwide. We hope the individual survey microdata, standardised dietary datasets, and global modelled data will each serve as critical public resources for researchers, health agencies and governments to evaluate national and subnational dietary intakes and trends, diet-related health burdens and disparities, dietary costs and affordability, strains and options for sustainability, corresponding policy and intervention priorities, and strengths and gaps in dietary surveillance. For example, ongoing efforts to biofortify staple foods in low-income nations^44 45^ will require data on national and subnational (eg, by age, sex, education, rural/urban residence) intakes of those staple foods, as well as on existing national and subnational intakes of the targeted nutrients from other foods, to effectively plan and implement biofortification.

Our investigation has several strengths. We performed systematic global searches for dietary surveys and employed standardised methods for survey and dietary factor identification, retrieval, processing, checking, standardisation and analysis. We searched multiple online databases of published literature and publicly available data, including region-specific databases, with extensive additional contacts of data owners to identify dietary surveys. We focused searches on data sparse world regions to improve the characterisation of diet in these populations and identify remaining dietary surveillance needs. We collected individual-level microdata or aggregate stratified data by key demographic subgroups, providing critical information on dietary heterogeneity within nations. We collected data on 54 foods, beverages, and nutrients, providing the most complete available information on overall diets. To maximise consistency and comparability, we performed standardised data extraction and analysis including data quality assessments, standardised food definitions and units, energy-adjusting intakes to age appropriate levels, and assessment of outliers and plausibility.

Limitations should be considered. Identified surveys used varying designs and instruments. Thus, GDD 2017 followed a rigorous documentation process to detail each survey’s methods and standardisation process to better standardise the data. Due to the breadth and scope of data collected and standardised, we focused on food categories (eg, fruits) rather than individual foods (eg, apples). Yet, food categories have been most often assessed in relation to MCH and NCD outcomes, and individual-level microdata are available in GDD for future assessments of more granular dietary categories. Four identified food categories (poultry, dairy-based desserts, candy and sweeteners, cakes, cookies and other baked goods) were excluded from our original assessment design; we hope to capture these categories in future iterations of the GDD. Not all potentially relevant dietary surveys could be retrieved due to accessing certain publicly available surveys and logistical challenges in contacting and engaging data owners.

In summary, the GDD 2017 identified, collated and standardised 1220 dietary surveys across 188 countries/territories globally, providing a public resource of data on 54 dietary factors in children and adults over time, nationally and subnationally by age, sex, urban/rural residence, education and pregnancy/lactation status; as well as identifying specific gaps for accelerated surveillance.
