# Supplementary material for: Global Dietary Database 2017: data availability and gaps on 54 major foods, beverages and nutrients among 5.6 million children and adults from 1220 surveys worldwide
Source: BMJ Glob Health. 2021 Feb 5;6(2):e003585. doi: 10.1136/bmjgh-2020-003585 (PMC7871251; doi:10.1136/bmjgh-2020-003585)
Supplement: Supplementary data [file bmjgh-2020-003585supp001.pdf]

Supplemental Materials

Supplement to:
Miller V, Singh GM, Onopa J, Reedy J, Shi P, Zhang J, Tahira A, Shulkin Morris ML, Marsden DM, Kranz S, Stoyell S, Webb P, Micha R, Mozaffarian D, Global Dietary Database

Contents

Appendix S1: Scope of data collection
Text S1. Description of geographic, demographic, and dietary scope of data collection
Table S1. Countries, regions and super-regions included in GDD 2017
Table S2. Survey- and participant-level characteristics included in GDD 2017

Appendix S2: Data identification and retrieval
Text S2. Overview of data search, identification, and retrieval process.
Table S3. Systematic review search terms for Medline, Web of Science, LILACS, African Index Medicus

Appendix S3: Data assessment and analysis
Text S3. Overview of data assessment, categorization, standardization, and analysis
Table S4. Total daily energy values by age
Table S5. Common categories of intake and their servings per day conversions
Table S6. Common weight conversions
Table S7. Standard serving sizes for foods and beverages
Table S8. Reference Adult Male Equivalents
Table S9. Group mean plausibility cutoffs for foods and beverages
Table S10. Individual mean plausibility cutoffs for foods and beverages
Table S11. Group mean plausibility cutoffs for macronutrients
Table S12. Individual mean plausibility cutoffs for macronutrients
Table S13. Group mean plausibility cutoffs for micronutrients
Table S14. Individual mean plausibility cutoffs for micronutrients
Text S4. Global population represented
Table S15. Characteristics of 1,220 dietary surveys in the GDD 2017 by dietary assessment method
Table S16. Frequency (%) of dietary factors by dietary assessment method
Figure S1. Geographic density of the number of dietary surveys in the GDD 2017 by country, including surveys collected between 1980 to 1999 (panel A) and 2000 to 2015 (panel B)

Appendix S4: Dissemination
Table S17. Data sharing agreements for corresponding member and public surveys.
Table S18. Characteristics of surveys included in the Global Dietary Database.

Appendix S1. Scope of data collection

Text S1. Description of geographic, demographic, and dietary scope of data collection

Between November 2014 and May 2017, we searched for global dietary intake data on 54 dietary factors, stratified by demographic characteristics of interest. Dietary factors included in GDD 2010 (21) were those with established diet-disease relationships. Dietary factors added to GDD 2017 (33) were considered relevant to chronic disease, maternal and child health, populations not assessed in GDD 2010 (children, adolescents, pregnant/nursing women), or data-sparse geographical regions.

Countries and time period of data collection
We searched for surveys conducted between 1980 and 2015 which measured dietary intakes in 207 countries worldwide, which were grouped into 7 super-regions based on geographic proximity and epidemiologic characteristics (Table S1).

Demographic characteristics
We searched for surveys with individual-level data capturing both diet and demographic characteristics of interest, including age, sex, urban vs. rural area of residence, level of education, and pregnancy status for women (Table S2). Classifications of urban or rural residence relied on survey-specific definitions based on population density and primary sources of employment; some country- and region-level variation existed in these definitions. Educational attainment was aggregated in three levels (<=6 years of education, 6.01-12 years, or 12.01+ years) and used as a proxy of socioeconomic status. When available, the head of household's education level was used for children who had not completed education. Individual-level or survey-level geocode data was also collected when available.

Survey characteristics
The sampling methodology, representativeness, sample size, and dietary assessment methods of each survey were recorded as part of the data collection process.

Selected dietary factors of interest
Individual-level data was collected on intake of 54 key foods and nutrients relevant to deficiency conditions, chronic disease, and overall health and diet quality.

Miller V, *et al. BMJ Global Health* 2021; 6:e003585. doi: 10.1136/bmjgh-2020-003585

Table S1. Countries, regions, and super-regions included in GDD 2017

Region	Countries
**Asia super-region (N=34)**	
East Asia (N=5)	China, Democratic People's Republic of Korea, Hong Kong, Macau, Taiwan
Southeast Asia (N=9)	Cambodia, Indonesia, Lao People's Democratic Republic, Malaysia, Myanmar, Philippines, Thailand, Timor-Leste, Viet Nam
Asia-Pacific high income (N=4)	Brunei Darussalam, Japan, Republic of Korea, Singapore
Oceania (N=16)	American Samoa, Cook Islands, Fiji, French Polynesia, Kiribati, Marshall Islands, Micronesia, Nauru, Palau, Papua New Guinea, Samoa, Solomon Islands, Tokelau, Tonga, Tuvalu, Vanuatu
**Former Soviet Union super-region (N=29)**	
Central Asia (N=9)	Armenia, Azerbaijan, Georgia, Kazakhstan, Kyrgyzstan, Mongolia, Tajikistan, Turkmenistan, Uzbekistan
Central Europe (N=13)	Albania, Bosnia and Herzegovina, Bulgaria, Croatia, Czech Republic, Hungary, Montenegro, Poland, Romania, Serbia, Slovakia, Slovenia, The former Yugoslav Republic of Macedonia
Eastern Europe (N=7)	Belarus, Estonia, Latvia, Lithuania, Republic of Moldova, Russian Federation, Ukraine
**Latin America and Caribbean super-region (N=41)**	
Caribbean (N=24)	Anguilla, Antigua and Barbuda, Bahamas, Barbados, Belize, Bermuda, British Virgin Islands, Cayman Islands, Cuba, Dominica, Dominican Republic, Grenada, Guadeloupe, Guyana, Haiti, Jamaica, Martinique, Netherlands Antilles, Puerto Rico, Saint Kitts and Nevis, Saint Lucia, Saint Vincent and the Grenadines, Suriname, Trinidad and Tobago
Andean Latin America (N=3)	Bolivia, Ecuador, Peru
Central Latin America (N=9)	Colombia, Costa Rica, El Salvador, Guatemala, Honduras, Mexico, Nicaragua, Panama, Venezuela
Southern Latin America (N=3)	Argentina, Chile, Uruguay
Tropical Latin America (N=2)	Brazil, Paraguay
**Middle East and Northern Africa super-region (N=20)**	
Western Europe (N=1)	Israel
North Africa and Middle East (N=19)	Algeria, Bahrain, Egypt, Iran (Islamic Republic of), Iraq, Jordan, Kuwait, Lebanon, Libyan Arab Jamahiriya, Morocco, Occupied Palestinian Territory, Oman, Qatar, Saudi Arabia, Syrian Arab Republic, Tunisia, Turkey, United Arab Emirates, Yemen
**South Asia super-region (N=9)**	
South Asia (N=6)	Afghanistan, Bangladesh, Bhutan, India, Nepal, Pakistan
Southeast Asia (N=3)	Maldives, Réunion, Sri Lanka
**Sub-Saharan Africa super-region (N=48)**	

Miller V, *et al. BMJ Global Health* 2021; 6:e003585. doi: 10.1136/bmjgh-2020-003585

Central Sub-Saharan Africa (N=6)	Angola, Central African Republic, Congo, Democratic Republic of Congo, Equatorial Guinea, Gabon
Eastern Sub-Saharan Africa (N=17)	Burundi, Comoros, Djibouti, Eritrea, Ethiopia, Kenya, Madagascar, Malawi, Mauritius, Mozambique, Rwanda, Seychelles, Somalia, Sudan, Uganda, United Republic of Tanzania, Zambia
Southern Sub-Saharan Africa (N=6)	Botswana, Lesotho, Namibia, South Africa, Swaziland, Zimbabwe
Western Sub-Saharan Africa (N=19)	Benin, Burkina Faso, Cameroon, Cape Verde, Chad, Côte d'Ivoire, Gambia, Ghana, Guinea, Guinea-Bissau, Liberia, Mali, Mauritania, Niger, Nigeria, São Tomé and Príncipe, Senegal, Sierra Leone, Togo
**High-Income Countries super-region (N=26)**	
Australasia (N=2)	Australia, New Zealand
Western Europe (N=22)	Andorra, Austria, Belgium, Cyprus, Denmark, Finland, France, Germany, Greece, Greenland, Iceland, Ireland, Italy, Luxembourg, Malta, Netherlands, Norway, Portugal, Spain, Sweden, Switzerland, United Kingdom
North America high-income (N=2)	Canada, United States of America

We included countries: 1) classified as United Nations (UN) Member States, 2) included in the United Nations Food and Agriculture Food Balance Sheets database, or 3) included in the World Bank Gross Domestic Product database.
Surveys were not identified for: Afghanistan, Andorra, Bermuda, Cuba, Democratic People's Republic of Korea, Djibouti, Equatorial Guinea, French Polynesia, Guadeloupe, Guinea-Bissau, Hong Kong, Macau, Martinique, Netherlands Antilles, Nicaragua, Palau, Réunion, Somalia, Turkmenistan.

Table S2. Survey- and participant-level characteristics included in GDD 2017

Variable Name	Variable Definition	Variable Coding
**Observation ID**	Unique numeric identifier for each observation	A number with 10 digits or less.
**Start year**	Year in which data collection began	A four-digit number between 1980 and 2014
**End year**	Year in which data collection ended	A four-digit number between 1981 and 2015 (enter -9 if data collection has not yet ended)
**Age**	This is a continuous variable that captures the age in years of the individual	Age in years. For all ages ≥ 2 years, enter the year as a whole number. For children under the age of two years, enter the age as a decimal (1 month=0.08 years, 2 months=0.17, 3 months=0.25, 4 months=0.33, 5 months=0.42, 6 months=0.5, 7 months=0.58, 8 months=0.67, 9 months=0.75, 10 months=0.83, 11 months=0.92).
**Sex**	This is a dichotomous indicator variable that describes the gender of the individual.	1=male<br>2=female
**Residence**	This is a dichotomous indicator variable that describes whether the individual lives in urban or rural area.	1=urban<br>2=rural<br>-9=information not available
**Education**	This is an indicator variable that describes the individual's level of education.	1=primary: 6 or fewer years of formal education<br>2=secondary: 6.01-12 years of formal education<br>3=tertiary: 12.01 or more years of formal education<br>-9 = information not available<br>*Note: for children and adolescents, the level of education refers to that of the head of household.*
**Pregnancy/Lactation**	This is a variable that indicates whether an individual is pregnant or nursing mother	0= not pregnant or nursing<br>1 = pregnant<br>2 = nursing<br>-9= information not available
**Geocode**	Latitude and longitude for the area representing the individual's place of permanent residence.	Latitude: a continuous variable from 0 to (+/-) 90 degrees, to the hundredth place.<br>Longitude: a continuous variable from 0 to (+/-) 180 degrees, to the hundredth place.
**Sample weight**	The sampling weight (i.e. probability weight), calculated as the inverse of the probability of a participant being included in the sample in the survey sampling design.	A continuous positive non-zero numeric variable.

**Primary sampling units**	The primary sampling unit (PSU) of the survey, i.e. the sampling unit selected in the primary stage of a multi-stage sample.	An indicator variable with integer values where each integer represents a level of the PSU.
**Stratum**	The mutually exclusive strata used in multi-stage stratified sampling.	An indicator variable with integer values where each integer represents a level of stratification.

Appendix S2. Data identification and retrieval

Text S2. Overview of data search, identification, and retrieval process

Data Identification

Surveys measuring dietary intakes were identified through three approaches:

- Systematic searches of available literature to identify publications on relevant surveys. Corresponding authors were then invited to collaborate and share data with the GDD. Searching databases of publicly-available data. Relevant surveys were identified, and data were downloaded when available.
- Re-contacting 199 corresponding members of the GDD 2010. Existing contacts were invited to update previously-shared data with additional demographic stratum or dietary factors, or to submit new data.

Review of published literature

We conducted a systematic review of literature published on population-based nutritional surveys around the world. Detailed information on the search terms is provided below and in Table S3. In summary, studies were included if they were from a population-based sample, had information on at least one dietary factor of interest, and collected data via a valid dietary assessment method from a population of at least 100 individuals. No restrictions were placed on the publication language and translators were utilized when applicable. The United States was excluded from the search, as data were already available for multiple individual-level nationally representative health examination surveys(2).

Medline search: We primarily searched Medline (via PubMed) for articles published between 1980 and the time the search was conducted, 2014-2015. All human infants, children, and adults were included.

LILACS database search: We searched the Latin American and Caribbean Health Sciences database in September and October 2015 for studies in data-sparse Latin American and Caribbean countries not captured via Medline search.

African Index Medicus and Web of Science search: We searched the African Index Medicus and Web of Science databases in October 2015 for studies not captured via the Medline search.

Publicly available data search

Publicly available online surveys containing dietary intake data were identified through suggestions from colleagues, searches of other published literature (including via systematic reviews), and through online web searches.

Data Inclusion

Published studies were included if the:

- Survey sample was not limited to a subgroup whose diet would significantly differ from the general population, e.g. only diabetics, only overweight/obese individuals, only specific ethnic minorities or immigrants.
- Survey was nationally, sub-nationally or locally representative.
- Survey had a sample size greater than 100.
- Survey had information on at least one of the dietary factors of interest.
- Survey was based on a valid dietary assessment method including multiple or single short-term diet recalls, and Food Frequency Questionnaires.

Surveys identified were included if they met the inclusion criteria and had not previously been identified through the GDD 2010. For the systematic review of published literature, all studies identified through the database search were assessed for inclusion by title/abstract by one reviewer. All studies marked for inclusion during title/abstract review were then assessed by full text, and author contact information was extracted from included studies for future correspondence. Subsets of studies were reviewed by a second reviewer to ensure consistency and accuracy of study inclusion/exclusion.

Data Retrieval

Corresponding authors from included studies were invited by email to collaborate with the GDD 2017 by sharing their available dietary intake data. Authors were invited to visit the GDD 2017 web application, register an account, complete a data-sharing agreement, enter characteristics of the survey they wish to share, and then submit dietary intake data from that survey. Survey data extraction was considered complete when authors successfully shared data, responded that data were unavailable for sharing, or were non-responsive to multiple contact attempts. Data owners were invited to share individual-level microdata, either formatted to match the GDD data dictionary in raw format, or aggregate level data to match GDD strata. Data owners who were unresponsive to initial invitation or who only partially completed the survey submission were re-contacted up to 6 times, and if available, at alternate email addresses.

Table S3. Systematic review search terms for Medline, Web of Science, LILACS, African Index Medicus

**Medline, Web of Science primary search**	(“nutrition”[tiab] OR “nutritional status”[MeSH] OR “diet”[tiab] OR “diet”[MeSH] OR “food habits”[MeSH] OR “food habits”[tiab] OR “fruit”[MeSH] OR “fruit”[tiab] OR “fruits”[tiab] OR “fruit juice” [tiab] OR “fruit juices” [tiab] OR “nuts”[MeSH] OR “nuts”[tiab] OR “nut”[tiab] OR “seeds”[MeSH] OR “seeds”[tiab] OR “seed”[tiab] OR “peanut*”[tiab] OR “vegetables”[MeSH] OR “vegetables”[tiab] OR “vegetable”[tiab] OR “potato”[tiab] OR “potatoes”[tiab] OR “solanum tuberosum”[MeSH] OR “starchy vegetables”[tiab] OR “lentil”[tiab] OR “lentils”[tiab] OR “peas”[MeSH] OR “bean”[tiab] OR “beans”[tiab] OR “legume”[tiab] OR “legumes”[tiab] OR “whole grains”[tiab] OR “whole grain”[tiab] OR “refined grains”[tiab] OR “refined grain”[tiab] OR “meat”[Mesh] OR “meat”[tiab] OR “meat products”[MeSH] OR “processed meat”[tiab] OR “fishes”[MeSH] OR “fish products”[MeSH] OR “fish”[tiab] OR “seafood”[MeSH] OR “seafood”[tiab] OR “egg”[tiab] OR “eggs”[tiab] OR “egg proteins, dietary”[MeSH] OR “milk” [MeSH] OR “milk” [tiab] OR “cultured milk products”[MeSH] OR “cultured milk products”[tiab] OR “fermented milk”[tiab] OR “yogurt”[MeSH] OR “yogurt”[tiab] OR “yoghurt”[tiab] OR “cheese”[MeSH] OR “cheese”[tiab] OR “cheeses”[tiab] OR “sugar-sweetened beverages” [tiab] OR “sugar-sweetened beverage” [tiab] OR “sweetened beverages” [tiab] OR “sweetened beverage” [tiab] OR “beverages” [MeSH] OR “sugary beverage”[tiab] OR “sugary beverages”[tiab] OR “coffee”[MeSH] OR “coffee”[tiab] OR “tea”[MeSH] OR “tea”[tiab] OR “dietary sucrose” [MeSH] OR “added sugar”[tiab] OR “added sugars”[tiab] OR “energy intake” [MeSH] OR “energy intake” [tiab] OR “dietary carbohydrates”[MeSH] OR “dietary carbohydrates”[tiab] OR “dietary proteins”[MeSH] OR “vegetable proteins”[MeSH] OR "saturated fat"[tiab] OR "fatty acids, unsaturated"[MeSH] OR “dietary fats, unsaturated”[MeSH] OR "polyunsaturated"[tiab] OR “omega-6”[tiab] OR “alpha-linolenic”[tiab] OR "oleic"[tiab] OR "linoleic"[tiab] OR "omega-3"[tiab] OR "fatty acids, omega-3"[MeSH] OR "monounsaturated fat"[tiab] OR "fatty acids, monounsaturated"[MeSH] OR "trans fatty acids"[MeSH] OR "trans-fatty"[tiab] OR "trans fatty"[tiab] OR "trans fat"[tiab] OR “cholesterol, dietary” [MeSH] OR “dietary cholesterol” [tiab] OR “dietary fiber” [MeSH] OR “dietary fiber” [tiab] OR “dietary fibers” [tiab] OR “sodium, dietary” [MeSH] OR “dietary sodium” [tiab] OR “sodium chloride”[MeSH] OR “salt”[tiab] OR "potassium, dietary"[MeSH] OR “dietary potassium”[tiab] OR “calcium, dietary” [MeSH] OR “dietary calcium” [tiab] OR “iron, dietary”[MeSH] OR “dietary iron”[tiab] OR “zinc”[MeSH] OR “dietary zinc”[tiab] OR “magnesium”[MeSH] OR “dietary magnesium”[tiab] OR “selenium”[MeSH] OR “dietary selenium”[tiab] OR “iodine”[MeSH] OR “dietary iodine”[tiab] OR “vitamin A”[MeSH] OR “retinoids”[MeSH] OR “carotenoids”[MeSH] OR “dietary vitamin A”[tiab] OR “vitamin D”[MeSH] OR “dietary vitamin D” [tiab] OR “vitamin E”[MeSH] OR “tocopherols”[MeSH] OR “dietary vitamin E”[tiab] OR “tocopherol”[tiab] OR “tocopherols”[tiab] OR “ascorbic acid”[MeSH] OR “dietary vitamin C”[tiab] OR “ascorbic acid”[tiab] OR “vitamin B complex”[MeSH] OR “thiamine”[MeSH] OR “dietary vitamin B1”[tiab] OR “dietary thiamine”[tiab] OR “riboflavin”[MeSH] OR “niacin”[MeSH] OR “pantothenic acid”[MeSH] OR “pyridoxine”[MeSH] OR “biotin”[MeSH] OR “folic acid”[MeSH] OR “vitamin B 6”[MeSH] OR “dietary riboflavin”[tiab] OR “dietary niacin”[tiab] OR “dietary pyridoxine”[tiab] OR “dietary biotin”[tiab] OR “dietary folate”[tiab] OR “dietary folic acid”[tiab] OR “vitamin B 12”[MeSH] OR “dietary vitamin B 12”[tiab] OR “dietary cobalamin”[tiab] OR “glycemic index”[MeSH] OR “glycemic index”[tiab] OR “glycemic load”[tiab] NOT “polymorphism”[tiab]) AND (“national survey”[tiab] OR “national surveys”[tiab] OR “subnational surveys”[tiab] OR “population-based”[tiab] OR “nutrition assessment”[MeSH] OR “nutrition surveys”[MeSH] OR “diet records”[MeSH] OR “diet surveys”[MeSH]) AND (Humans[Mesh])AND ("1980/01/01"[PDat] : "3000/12/31"[PDat])AND ("country name" [MeSH])
**Medline, Web of Science secondary search**	(“nutrition”[tiab] OR “nutritional status”[MeSH] OR “diet”[tiab] OR “diet”[MeSH] OR “food habits”[MeSH] OR “food habits”[tiab] OR “fruit”[MeSH] OR “fruit”[tiab] OR “fruits”[tiab] OR “fruit juice” [tiab] OR “fruit juices” [tiab] OR “nuts”[MeSH] OR “nuts”[tiab] OR “nut”[tiab] OR “seeds”[MeSH] OR “seeds”[tiab] OR “seed”[tiab] OR “peanut*”[tiab] OR “vegetables”[MeSH] OR “vegetables”[tiab] OR “vegetable”[tiab] OR “potato”[tiab] OR “potatoes”[tiab] OR “solanum tuberosum”[MeSH] OR “starchy vegetables”[tiab] OR “lentil”[tiab] OR “lentils”[tiab] OR “peas”[MeSH] OR “bean”[tiab] OR “beans”[tiab] OR “legume”[tiab] OR “legumes”[tiab] OR “whole grains”[tiab] OR “whole grain”[tiab] OR “refined grains”[tiab] OR “refined grain”[tiab] OR

	"meat"[Mesh] OR "meat"[tiab] OR "meat products"[MeSH] OR "processed meat"[tiab] OR "fishes"[MeSH] OR "fish products"[MeSH] OR "fish"[tiab] OR "seafood"[MeSH] OR "seafood"[tiab] OR "egg"[tiab] OR "eggs"[tiab] OR "egg proteins, dietary"[MeSH] OR "milk" [MeSH] OR "milk" [tiab] OR "cultured milk products"[MeSH] OR "cultured milk products"[tiab] OR "fermented milk"[tiab] OR "yogurt"[MeSH] OR "yogurt"[tiab] OR "yoghurt"[tiab] OR "cheese"[MeSH] OR "cheese"[tiab] OR "cheeses"[tiab] OR "sugar-sweetened beverages" [tiab] OR "sugar-sweetened beverage" [tiab] OR "sweetened beverages" [tiab] OR "sweetened beverage" [tiab] OR "beverages" [MeSH] OR "sugary beverage"[tiab] OR "sugary beverages"[tiab] OR "coffee"[MeSH] OR "coffee"[tiab] OR "tea"[MeSH] OR "tea"[tiab] OR "dietary sucrose" [MeSH] OR "added sugar"[tiab] OR "added sugars"[tiab] OR "energy intake" [MeSH] OR "energy intake" [tiab] OR "dietary carbohydrates"[MeSH] OR "dietary carbohydrates"[tiab] OR "dietary proteins"[MeSH] OR "vegetable proteins"[MeSH] OR "saturated fat"[tiab] OR "fatty acids, unsaturated"[MeSH] OR "dietary fats, unsaturated"[MeSH] OR "polyunsaturated"[tiab] OR "omega-6"[tiab] OR "alpha-linolenic"[tiab] OR "oleic"[tiab] OR "linoleic"[tiab] OR "omega-3"[tiab] OR "fatty acids, omega-3"[MeSH] OR "monounsaturated fat"[tiab] OR "fatty acids, monounsaturated"[MeSH] OR "trans fatty acids"[MeSH] OR "trans-fatty"[tiab] OR "trans fatty"[tiab] OR "trans fat"[tiab] OR "cholesterol, dietary" [MeSH] OR "dietary cholesterol" [tiab] OR "dietary fiber" [MeSH] OR "dietary fiber" [tiab] OR "dietary fibers" [tiab] OR "sodium, dietary" [MeSH] OR "dietary sodium" [tiab] OR "sodium chloride"[MeSH] OR "salt"[tiab] OR "potassium, dietary"[MeSH] OR "dietary potassium"[tiab] OR "calcium, dietary" [MeSH] OR "dietary calcium" [tiab] OR "iron, dietary"[MeSH] OR "dietary iron"[tiab] OR "zinc"[MeSH] OR "dietary zinc"[tiab] OR "magnesium"[MeSH] OR "dietary magnesium"[tiab] OR "selenium"[MeSH] OR "dietary selenium"[tiab] OR "iodine"[MeSH] OR "dietary iodine"[tiab] OR "vitamin A"[MeSH] OR "retinoids"[MeSH] OR "carotenoids"[MeSH] OR "dietary vitamin A"[tiab] OR "vitamin D"[MeSH] OR "dietary vitamin D" [tiab] OR "vitamin E"[MeSH] OR "tocopherols"[MeSH] OR "dietary vitamin E"[tiab] OR "tocopherol"[tiab] OR "tocopherols"[tiab] OR "ascorbic acid"[MeSH] OR "dietary vitamin C"[tiab] OR "ascorbic acid"[tiab] OR "vitamin B complex"[MeSH] OR "thiamine"[MeSH] OR "dietary vitamin B1"[tiab] OR "dietary thiamine"[tiab] OR "riboflavin"[MeSH] OR "niacin"[MeSH] OR "pantothenic acid"[MeSH] OR "pyridoxine"[MeSH] OR "biotin"[MeSH] OR "folic acid"[MeSH] OR "vitamin B 6"[MeSH] OR "dietary riboflavin"[tiab] OR "dietary niacin"[tiab] OR "dietary pyridoxine"[tiab] OR "dietary biotin"[tiab] OR "dietary folate"[tiab] OR "dietary folic acid"[tiab] OR "vitamin B 12"[MeSH] OR "dietary vitamin B 12"[tiab] OR "dietary cobalamin"[tiab] OR "glycemic index"[MeSH] OR "glycemic index"[tiab] OR "glycemic load"[tiab] NOT "polymorphism"[tiab]) AND ("national survey"[tiab] OR "national surveys"[tiab] OR "subnational surveys"[tiab] OR "population-based"[tiab] OR "nutrition assessment"[MeSH] OR "nutrition surveys"[MeSH] OR "diet records"[MeSH] OR "diet surveys"[MeSH] OR "diet recall"[tiab] OR "food frequency questionnaire"[tiab] OR "FFQ"[tiab] OR "diet record"[tiab] OR "diet records"[tiab] OR "household surveys"[tiab] OR "household survey"[tiab]) AND (Humans[Mesh])AND ("1980/01/01"[PDat] : "3000/12/31"[PDat])AND ("country name" [MeSH])
**LILACS search**	(nutrition OR "nutritional status" OR diet OR "food habits" OR fruit OR fruits OR "fruit juice" OR "fruit juices" OR nuts OR nut OR seeds OR seed OR peanut OR vegetables OR vegetable OR potato OR potatoes OR "solanum tuberous" OR "starchy vegetables" OR lentil OR lentils OR peas OR bean OR beans OR legume OR legumes OR "whole grains" OR "whole grain" OR "refined grains" OR "refined grain" OR meat OR "meat products" OR "processed meat" OR "processed meats" OR fish OR fishes OR "fish products" OR seafood OR egg OR eggs OR "egg protein" OR milk OR "cultured milk products" OR "fermented milk" OR yogurt OR yoghurt OR cheese OR cheeses OR "sugar-sweetened beverages" OR "sugar-sweetened beverage" OR "sweetened beverage" OR "sweetened beverages" OR beverages OR beverage OR "sugary beverages" OR "sugary beverage" OR "coffee" OR tea OR "dietary sucrose" OR "added sugar" OR "added sugars" OR "energy intake" OR "dietary carbohydrates" OR "dietary carbohydrate" OR "dietary protein" OR "dietary proteins" OR "vegetable protein" OR "saturated fat" or "unsaturated fatty acids" OR "unsaturated dietary fats" OR polyunsaturated OR "omega-6" OR "alpha-linolenic" OR oleic OR linoleic OR "omega-3" OR "omega-3 fatty acids" OR "monounsaturated fat" OR "monounsaturated fatty acids" OR "trans fatty acids" OR "trans-fatty" OR "trans fatty" OR "trans fat" OR "dietary cholesterol" OR "dietary fiber" OR "dietary fibers" OR "dietary sodium" OR

	"sodium chloride" OR salt OR "dietary potassium" OR "dietary calcium" OR "dietary iron" OR zinc OR "dietary zinc" OR magnesium OR "dietary magnesium" OR selenium OR "dietary selenium" OR iodine OR "dietary iodine" OR "vitamin A" OR retinoids OR carotenoids OR "dietary vitamin A" OR "vitamin D" OR "dietary vitamin D" OR "vitamin E" OR tocopherols OR "dietary vitamin E" OR tocopherol OR "ascorbic acid" OR "dietary vitamin C" OR "vitamin B complex" OR thiamine OR "dietary vitamin B1" OR "dietary thiamine" OR riboflavin OR niacin OR "pantothenic acid" OR pyridoxine OR biotin OR "folic acid" OR "vitamin B 6" OR "dietary riboflavin" OR "dietary niacin" OR "dietary pyridoxine" OR "dietary biotin" OR "dietary folate" OR "dietary folic acid" OR "vitamin B 12" OR "dietary vitamin B 12" OR "dietary cobalamin" OR "glycemic index" OR "glycemic load") AND ("national survey" OR "national surveys" OR "population-based" OR "nutrition assessment" OR "nutrition surveys" OR "diet record" OR "diet surveys") AND (“country name”)
**African Index Medicus search**	(national survey OR national surveys OR subnational surveys OR population-based OR nutrition assessment OR nutrition surveys OR diet records OR diet record OR diet surveys OR diet recall OR food frequency questionnaire OR FFQ OR diet records OR household surveys OR household survey [Descriptor])

Miller V, *et al. BMJ Global Health* 2021; 6:e003585. doi: 10.1136/bmjgh-2020-003585

Appendix S3. Data assessment and analysis

Text S3. Description of data assessment, standardization, analysis, and storage

Data Storage

Data received through the GDD web application or GDD email account were stored on the Tufts GDD Box. Survey-specific folders were generated to securely store original data files, survey information files, original publications, and any communication from corresponding members.

Data Assessment

Data received from corresponding members or from publicly available surveys were checked to confirm survey-level characteristics; dietary intake variables were categorized into GDD dietary factors; necessary unit and format conversions were noted. Data-owners or survey directors were contacted extensively to resolve questions about data quality, categorization, or assessment methods to ensure accuracy and completeness of data prior to analysis.

Preliminary Data Checks

Biostatisticians generated survey description files for each survey including survey characteristics, variable lists, and summary statistics for categorical and continuous variables. Research assistants used these description files to assess inclusion of survey level information and demographic variables. Discrepancies between author-reported characteristics and those ultimately included in the dataset were noted for further discussion with the data-owner.

Categorization of variables into GDD dietary factors

Research assistants matched reported dietary data to GDD dietary factors. This involved categorizing foods, nutrients, mixed dishes, and regional items into the matched GDD dietary factor, noting cases where variables represented less than the optimal GDD definition. Unit conversions were included as necessary to transform variables into the optimal GDD units.

Categorization of variables into GDD dietary factors – Food Frequency Questionnaires

To transform food and beverage data reported from Food Frequency Questionnaires into optimal GDD units, most often grams per day, all categorical variables were standardized into single daily serving units (see Table S5). When ranges of frequencies were provided, the mean of each range was utilized to represent each frequency category. Variables reported in times per week were divided by 7 to calculate the average daily servings. Variables reported in times per month were divided by 30.42 (the average number of days in a month) to calculate the average daily servings. In cases where the upper range was open-ended (e.g., "5 or more times per week"), the ranges of the other frequency categories were used to calculate an upper limit. Servings per day were then converted into grams per day by multiplying the number of servings by the author-reported serving sizes or by the GDD standard serving sizes(3)(Table S6).

Communication with data owners and creation of data key

Any questions regarding the data, including those about region-specific diets (e.g., disaggregation of mixed dishes, classification of regional items), survey-level characteristics, and serving sizes for foods and beverages, were communicated to the data owner. After all questions regarding the data were answered, research assistants generated a data key outlining all available variables of interest, including demographic and dietary variables. Categorizations of dietary variables were turned into STATA code for clear identification and research assistants flagged the quality assurance checks.

Quality Control Part I: Ensuring accuracy of dietary variables

After being completed by a primary reviewer, a second research assistant reviewed the preliminary checks and data key to ensure that variables were classified, conversions were applied, and coding was written properly before forwarding on to statisticians for aggregation. Any questions about the data were discussed amongst the reviewers or the entire GDD core team and, when appropriate, the corresponding member was contacted to clarify any discrepancies.

Converting household data to individual data

Household-level data were transformed into individual-level data using the Adult Male Equivalent (AME) method (Table S8). The AME method estimates individual-level intakes by assigning each household member a reference AME based on their age and sex(4). Household members' reference AMEs are summed to find total household AME. Each individual's reference AME is then divided by the total household AME to find individual-level AME. This individual-level AME represents the proportion each individual contributes to the overall household AME. This individual level proportion is multiplied by the household consumption of each food item to estimate individual-level intake.

Data aggregation

Using preliminary checking documents provided by research assistants, biostatisticians converted individual-level data into aggregated outputs for each dietary factor stratified by the available demographic variables. Stata version 12 was used to convert all demographic and dietary variables from raw data files to a single data file containing only relevant variables. Missing observations were excluded from the dataset and all variables were recoded to match the GDD demographic and dietary variable coding scheme. Data were then aggregated into demographic strata by age, sex, residence, education, and pregnancy/lactating status. In addition to the single, final data file, supporting files were generated including a summary report, minimum/maximum values for each dietary factor, and group level means, standard deviations, and percentiles of intake. All output files were stored in each survey's specific folder on the Tufts GDD Box, accessible to all members of the research team.

Energy adjustment

We extracted both raw and energy-adjusted data when available. If energy adjustment of individual-level data had not been completed by the data owner, biostatisticians completed energy adjustment at the aggregation stage to age-specific levels (Table S4) using the residual method (5). This approach was considered the "gold standard." We adjusted for total energy intake to mitigate the effects of measurement error in data collection, account for differences in energy requirements related to body size, metabolic efficiency and physical activity, and facilitate comparisons between surveys, age groups, and sexes.

Table S4. Total daily energy values by age

Age (years)	Daily energy intake (kcal)
0-0.9	700
1-1.9	1,000
2-5	1,300
6-10	1,700
11-74	2,000
75+	1,700

Child and older adult-specific daily energy values were selected using dietary recommendations and mean population ranges from the USA (6,7), United Kingdom (8) and India (9).

Energy adjustment corrections

We initially asked that all data be shared both unadjusted and energy-adjusted to 2,000 kcal, regardless of age category, but retrospectively changed this decision to reflect the age-specific levels in Table S4. When possible, energy adjustment using the residual method was repeated to reflect these changes. In some cases, this approach was not possible, and thus alternative approaches for energy adjustment correction were taken.

Energy adjustment correction of aggregate ("stratum-level") data

In some cases, data were provided or accessed at the stratum level (i.e. age group, sex, education level, etc.). In these cases, energy adjustment correction depended on whether 2,000 kcal/day-adjusted values had previously been provided by the data owner. If energy-adjusted data had been provided, a simple ratio of the age-specific level to 2,000 kcal was applied post-hoc to convert the value to the correct energy level. If stratum-level data were only provided in an unadjusted format but with corresponding total energy intake, intake was adjusted to the age-specific energy level using the energy density method, in which a simple ratio of reported calorie intake to age-specific level was applied to the unadjusted value. If stratum-level data were provided in age groups which traversed more than one level of age-specific energy intake, a weighted mean daily energy intake was calculated. This weighted mean daily energy level was then used to adjust intake using the ratio readjustment method. If only unadjusted intake was available, the energy density method was used.

Energy adjustment of data without adjusted values or total energy intake

In limited cases, individual-level data were not initially energy-adjusted or provided with mean caloric intake data, precluding the use of the gold standard and ratio readjustment methods. In these instances, daily per capita energy availability data from FAO Food Balance

Sheets (FBS) were used to inform stratum-level caloric intake, following methods described by Del Gobbo et al., (6). In short, country-year-specific FBS energy data were adjusted using coefficients derived from a multivariate linear regression of GDD input data, FBS data, and both regional and survey-level covariates. Adjusted FBS energy was then corrected to the prescribed energy level by applying a factor of the energy level's proportion of 2,000 kcal. Unadjusted food and nutrient intake values were then adjusted with this corrected energy intake via the energy density method.

Quality Control Part II: Survey Plausibility and Quality Assessment

Group level means were assessed for plausibility by flagging any values that fell beyond a biologically plausible range (Tables S9, S11, S13). If means were flagged as implausible, they were assessed for correctness within the research team and discussed with the data owner to determine inclusion status. Additionally, even if within the predetermined plausibility range, if a stratum mean appeared vastly different from other, similar strata from the same survey, the original data was examined for extreme outliers that could account for this difference.

Quality checks were performed by a different research assistant to ensure all data checking procedures had been completed accurately.

Data Finalization

After data has been finalized for inclusion, it is stored within the Access database, which houses information on all surveys, corresponding authors, and survey checking statuses. Aggregated data is collated by dietary factor and prepared for input into the GDD prediction model.

Protocol for Converting FFQ Frequency Data into GDD Servings

1. Step 1- Standardize the categorical frequency variables to a single daily serving unit (Table S5 for examples)
   a. If a range of frequencies is given, take the mean ("Avg") of the range
   b. If the frequency is presented in times/week, divide by 7 (for days in a week)
   c. If the frequency is presented in times/month, divide by 30.42 (average days in a month)
      i. ***Note:*** If the category is presented as days/week instead of times/week, assume one serving per day and treat as times/week
      ii. Example A) 5-7 days/week= (6 days/week)/(7days/week)=0.857 servings/day
      iii. Example B) 1-3 times/month=(2 times/month)/(30.42 days/month)=0.066 servings/day
   d. If the upper range is open ended, use the range of the other frequency categories in the survey to create an upper limit and then take the average of that range.
      i. Example: "5 or more times per week" where next lowest level is 2-4 times per week. Assume a range of 5-7 times per week, take the average (6 times per week)/(7days/week)=0.857 servings/day
2. Step 2- Convert servings to grams **[Exception: Tea and coffee should be converted to cups]**
   a. If available, survey-specific serving sizes were used for conversions.
   b. If survey-specific serving sizes are not available, ask the data owner for usual, country-specific serving sizes.
   c. If data owner does not provide country-specific serving sizes, utilize country-specific serving sizes identified from national agencies (e.g. USDA).
   d. If no country-specific serving sizes are identified, use the GDD standard serving size conversions (Table S7).

Table S5. Common Categories of Intake and Their Servings per Day Conversions

Categorical Variable	Calculation	Daily Serving
Never	0	0
Occasional-Few times/year*	Should capture the range of values between never and the next highest choice based on the data set	*Depends on next level categorization
Less than once a month (1-11 times per year)	1+11=12/2=6 Avg servings/year<br>6/12 months=0.5 servings/month<br>0.5/30.42 days	0.0164
1-3 times/month	1+3=4/2=2 Avg servings/month<br>2/30.42 days	0.066
1/week	1 servings/7days	0.143
2-4 days/week	2+4=6/2=3 Avg servings/week<br>3servings/7days	0.429
5-6 days/week	5+6=11/2= 5.5 Avg servings/week<br>5.5/7days	0.786
5-7 days/week	5+7=12/2=6 Avg servings/week<br>6/7days	0.857
1/day		1
2-3/day	2+3=5/2= Avg 2.5 servings	2.5
4-5/day	4+5=9/2= Avg 4.5 servings	4.5

Table S6. Common Weight Conversions

**Provided weight**	**Grams**
1 Kilogram	1000
1 Ounce *Cannot use for fluid ounces	28
1 Pound	454

Table S7. Standard serving sizes for foods and beverages

Dietary factor		Reference serving sizes	"Usual" average serving sizes (g/serving)		
**Variable Code**	**Variable name**	**2003-06 US NHANES (median)**	**Adults and children older than 2 years**	**12-24 months**	**6 to 11 months**
v01	Fruits°	110 g per serving	100	75	49
v02	Non-starchy vegetables°	40 g per serving	100	50	44
v03	Potatoes*	-	186	61	34
v04	Other starchy vegetables*	-	160	47	42
v05	Beans/legumes°	86.5 g per serving	100	32	24
v06	Nuts/seeds	29.75 g per serving	28.35	32	24
v07<br>v08	Grains (refined and whole)°	48.975 g per serving	50	39	12
v09	Processed meats	53.705 g per serving	50	41	31
v10	Red meats	85 g per serving	100	32	24
v11	Seafood*	85.78 g per serving	85	30	23
v12	Eggs*	-	55	59	44
v13	Cheese*	-	42	22	20
v14	Yogurt*	-	245	104	88
v15	Sugar-sweetened beverages*	368 g per serving	248	130	84
v16	Fruit juices*	209.25 g per serving	248	130	84
v17	Coffee*	-	248	129	82
v18	Tea*	-	237	129	82
v19<br>v20<br>v57	Whole fat & reduced fat milk*	198.25 g per serving	245	161	155

*Calculated using average of item-specific serving sizes from the USDA Nutrient Database.
°Calculated using average if both item-specific serving sizes from the USDA Nutrient Database and intake from NHANES 2003-2006 (7).

Adult Male Equivalent Methodology

Steps:

Step 1: Identify each household and all members within that household.
Step 2: Identify the age and sex of all members of a household.
Step 3: Using age and sex, assign a reference AME to each household member as listed in Appendix A.
Step 4: Sum the AMEs from each household member to find the total household AMEs.
Step 5: Divide each household member's reference AME by the total household AME to determine individual AME.
Step 6: Multiply the individual AME by the total household consumption estimate individual intake.

Table S8. Reference Adult Male Equivalents

	Adult Male Equivalent			
**AGE GROUP (y)**	**MALE**	**FEMALE (non-pregnant/ non-lactating)**	**FEMALE (pregnant)**	**FEMALE (lactating)**
0-1	0.213	0.197		
1-2	0.311	0.279		
2-3	0.369	0.344		
3-4	0.410	0.377		
4-5	0.443	0.410		
5-6	0.484	0.434		
6-7	0.516	0.467		
7-8	0.557	0.508		
8-9	0.598	0.557		
9-10	0.648	0.607		
10-11	0.705	0.656		
11-12	0.770	0.705		
12-13	0.836	0.746	0.839	0.932
13-14	0.910	0.779	0.872	0.965
14-15	0.984	0.803	0.897	0.989
15-16	1.041	0.820	0.913	1.006
16-17	1.090	0.820	0.913	1.006
17-18	1.115	0.820	0.913	1.006
18-30	1.000	0.779	0.872	0.965
30-60	0.984	0.779	0.872	0.965
60-150	0.811	0.697		

Miller V, *et al. BMJ Global Health* 2021; 6:e003585. doi: 10.1136/bmjgh-2020-003585

Table S9. Group mean plausibility cutoffs for foods and beverages

Dietary Factor[1, 2]	Age group	Serving Size[3,4]	Serving Size Units	Group mean minimum serving/day	Group mean minimum g/day	Group mean max serving/day	Group mean max g/day
Fruits	All	100	grams	≤0	≤0	4	400
Non-starchy vegetables	All	100	grams	≤0	≤0	4	400
Potatoes	All	150	grams	≤0	≤0	3	450
Other starchy vegetables	All	150	grams	≤0	≤0	3	450
Beans/legumes	All	100	grams	≤0	≤0	4	400
Nuts/seeds	All	28	grams	≤0	≤0	3	84
Refined grains	All	50	grams	≤0	≤0	6	300
Whole grains	All	50	grams	≤0	≤0	3	150
Processed meats	All	50	grams	≤0	≤0	2	100
Unprocessed red meats	All	100	grams	≤0	≤0	2	200
Seafood	All	100	grams	≤0	≤0	2	200
Eggs	All	55	grams	≤0	≤0	2	110
Cheese	All	42	grams	≤0	≤0	2	84
Yogurt	All	245	grams	≤0	≤0	2	490
Sugar-sweetened beverages	Children	248	grams	≤0	≤0	6	1488
	Adults	248	grams	≤0	≤0	4	992
Fruit Juices	All	248	grams	≤0	≤0	3	744
Coffee	Children	1	cups	≤0	≤0	2	2
	Adults	1	cups	≤0	≤0	3	3
Tea	Children	1	cups	≤0	≤0	3	3
	Adults	1	cups	≤0	≤0	6	6
Reduced fat milk	All	245	grams	≤0	≤0	2	490
Whole fat milk	All	245	grams	≤0	≤0	2	490
Total Milk	All	245	grams	≤0	≤0	2	490

1. The optimal unit for all dietary factors, except coffee and tea, is energy-adjusted grams/day. For coffee and tea the optimal unit is energy adjusted cups/day
2. Unless indicated, all plausibility values apply to all age groups. The children category includes ages 14 years and below.
3. Serving sizes were calculated by searching the USDA Nutrient Database for serving sizes of the most common types of each dietary factor. These values were then averaged together to estimate an overall serving size.
4. Recommended intakes are based off the US 2015-2020 Dietary Guidelines and were extracted from http://www.choosemyplate.gov/MyPlate (11). These values are appropriate for individuals with moderate caloric intake (2000 kcal) and less than 30 minutes per day of moderate physical activity.

Table S10. Individual mean plausibility cutoffs for foods and beverages

Dietary Factor[1, 2]	Age group	Serving Size[3,4]	Serving Size Units	Individual mean minimum serving/day	Individual mean minimum g/day	Individual mean max serving/day	Individual mean max g/day
Fruits	All	100	grams	≤0	≤0	15	1500
Non-starchy vegetables	All	100	grams	≤0	≤0	10	1000
Potatoes	All	150	grams	≤0	≤0	10	1500
Other starchy vegetables	All	150	grams	≤0	≤0	10	1500
Beans/legumes	All	100	grams	≤0	≤0	10	1000
Nuts/seeds	All	28	grams	≤0	≤0	10	280
Refined grains	All	50	grams	≤0	≤0	15	750
Whole grains	All	50	grams	≤0	≤0	10	500
Processed meats	All	50	grams	≤0	≤0	10	500
Unprocessed red meats	All	100	grams	≤0	≤0	10	1000
Seafood	All	100	grams	≤0	≤0	10	1000
Eggs	All	55	grams	≤0	≤0	10	550
Cheese	All	42	grams	≤0	≤0	10	420
Yogurt	All	245	grams	≤0	≤0	10	2450
Sugar-sweetened beverages	Children	248	grams	≤0	≤0	15	3720
	Adults	248	grams	≤0	≤0	15	3720
Fruit Juices	All	248	grams	≤0	≤0	10	2480
Coffee	Children	1	cups	≤0	≤0	15	15
	Adults	1	cups	≤0	≤0	15	15
Tea	Children	1	cups	≤0	≤0	15	15
	Adults	1	cups	≤0	≤0	15	15
Reduced fat milk	All	245	grams	≤0	≤0	10	2450
Whole fat milk	All	245	grams	≤0	≤0	10	2450
Total Milk	All	245	grams	≤0	≤0	10	2450

1. The optimal unit for all dietary factors, except coffee and tea, is energy-adjusted grams/day. For coffee and tea the optimal unit is energy adjusted cups/day
2. Unless indicated, all plausibility values apply to all age groups. The children category includes ages 14 years and below.
3. Serving sizes were calculated by searching the USDA Nutrient Database for serving sizes of the most common types of each dietary factor. These values were then averaged together to estimate an overall serving size.
4. Recommended intakes are based off the US 2015-2020 Dietary Guidelines and were extracted from http://www.choosemyplate.gov/MyPlate (11). These values are appropriate for individuals with moderate caloric intake (2000 kcal) and less than 30 minutes per day of moderate physical activity.

Miller V, *et al. BMJ Global Health* 2021; 6:e003585. doi: 10.1136/bmjgh-2020-003585

Table S11. Group mean plausibility cutoffs for macronutrients

Dietary Factor[1]	GDD unit	Group Adult Mean min.	Group Adult Mean max.	Group Child Mean min.[2]	Group Child Mean max.
Energy	kcal/day	≤1000	≥4000	≤400	≥3000
Carbohydrate	% kcal	≤25	≥80	≤25	≥80
Total Protein	% kcal	≤5	≥30	≤5	≥30
Total Protein cont.[3]	g/day (based off 2000 kcal diet)	≤25	≥150	≤25	≥150
Animal Protein	% kcal	0	≥30	0	≥30
Animal Protein cont.[3]	g/day (based off 2000 kcal diet)	≤0	≥150	≤0	≥150
Plant Protein	% kcal	0	≥20	0	≥20
Plant Protein, cont[3]	g/day (based off 2000 kcal diet)	≤10	≥150	≤10	≥150
Saturated Fat	% kcal	0	≥40	0	≥35
Monounsaturated fat	% kcal	≤5	≥30	≤3	≥30
Omega 6 Fat	% kcal	≤1	≥20	≤1	≥20
Seafood Omega 3 Fat	mg/day	≤0	≥4000	≤0	≥400
Plant Omega 3	mg/day	≤200	≥6000	≤300	≥4000
Trans fat	% kcal	≤0	≥15	≤0	≥8
Dietary Cholesterol	mg/day	≤50	≥1000	<50	≥800
Dietary Fiber	g/day	≤5	≥60	≤5	≥40
Added Sugar	% kcal	≤5	>45	≤5	>45

1. Maximum values were defined using observed population intakes and dietary recommendations (11-34).
2. Child category includes ages 14 and below.
3. Protein gram estimates are based on 2000 kcal diet and the knowledge that 1 gram of protein contain 4 kcal.

Table S12. Individual mean plausibility cutoffs for macronutrients

Dietary Factor[1]	GDD unit	Individual Mean min.	Individual Adult Mean max.	Individual Child Mean max.[2]
Energy	kcal/day	≤500	≥8000	≥4000
Carbohydrate	% kcal	≤15	≥80	≥80
Total Protein	% kcal	≤3	≥40	≥40
Total Protein cont.[3]	g/day (based off 2000 kcal diet)	≤15	≥200	≥200
Animal Protein	% kcal	<0	≥40	≥40
Animal Protein cont.[3]	g/day (based off 2000 kcal diet)	≤0	≥200	≥200
Plant Protein	% kcal	0	≥30	≥30
Plant Protein, cont[3]	g/day (based off 2000 kcal diet)	≤0	≥150	≥150
Saturated Fat	% kcal	≤3	≥50	≥45
Monounsaturated fat	% kcal	≤5	≥40	≥35
Omega 6 Fat	% kcal	≤1	≥50	≥40
Seafood Omega 3 Fat	mg/day	≤0	≥2500	≥1000
Plant Omega 3	mg/day	≤0	≥6000	≥4000
Trans fat	% kcal	≤0	≥20	≥15
Dietary Cholesterol	mg/day	≤25	≥3000	≥1500
Dietary Fiber	g/day	≤0	≥150	≥80
Added Sugar	% kcal	≤0%	>60	>60

1. Maximum values were defined using observed population intakes and dietary recommendations (11-34).
2. Child category includes ages 14 and below.
3. Protein gram estimates are based on 2000kcal diet and the knowledge that 1 gram of protein contain 4 kcal.

Table S13. Group mean plausibility cutoffs for micronutrients

Dietary Factor[1]	GDD unit	Group Adult Mean min.	Group Adult Mean max.	Group Child Mean min.[2]	Group Child Mean max.
Calcium	mg/day	≤300	≥3000	≤100	≥1500
Sodium	mg/day	≤500	≥9000	≤500	≥3000
Iodine	ug/day	≤30	≥800	≤20	≥150
Iron	mg/day	≤5	≥40	≤2	≥30
Magnesium	mg/day	≤100	≥700	≤25	≥400
Potassium	mg/day	≤1000	≥8000	≤500	≥6000
Selenium	ug/day	≤15	≥300	≤8	≥80
Vit. A with supplement	ug RAE/day	≤400	≥5000	≤200	≥1500
Vit. A without supplement	ug RAE/day	≤200	≥3000	≤50	≥600
Vitamin B1	mg/day	≤0.5	≥4	≤0.25	≥2
Vitamin B2	mg/day	≤0.5	≥5	≤0.25	≥2
Vitamin B3	mg/day	≤5	≥60	≤2	≥30
Vitamin B6	mg/day	≤0.5	≥5	≤0.25	≥2
Vitamin B9 (Folate)	ug/day	≤100	≥1000	≤50	≥500
Vitamin B12	ug/day	≤1	≥15	≤0.5	≥10
Vitamin C	mg/day	≤25	≥400	≤10	≥125
Vitamin D	ug/day	≤1	≥50	≤1	≥25
Vitamin E	mg/day	≤3	≥30	≤1	≥15
Zinc	mg/day	≤3	≥50	≤1	≥25

1. Maximum values were defined using observed population intakes and dietary recommendations (11-34).
2. Child category includes ages 14 and below.

Table S14. Individual mean plausibility cutoffs for micronutrients

Dietary Factor[1]	GDD unit	Individual Adult Mean min.	Individual Adult Mean max.	Individual Child Mean max.[2]
Calcium	mg/day	≤15	≥4000	≥3500
Sodium	mg/day	≤500	≥12000	≥5000
Iodine	ug/day	≤15	≥1100	≥200
Iron	mg/day	≤2	≥150	≥75
Magnesium	mg/day	≤10	≥1000	≥800
Potassium	mg/day	≤250	≥15000	≥10000
Selenium	ug/day	≤5	≥900	≥200
Vit. A with supplement	ug RAE/day	≤200	≥15000	≥3000
Vit. A without supplement	ug RAE/day	≤25	≥10000	≥1000
Vitamin B1	mg/day	≤0.2	≥15	≥5
Vitamin B2	mg/day	≤0.2	≥15	≥5
Vitamin B3	mg/day	≤1	≥120	≥80
Vitamin B6	mg/day	≤0.2	≥20	≥6
Vitamin B9 (Folate)	ug/day	≤25	≥2000	≥1000
Vitamin B12	ug/day	≤0.5	≥200	≥50
Vitamin C	mg/day	≤5	≥2500	≥600
Vitamin D	ug/day	≤0.5	≥200	≥100
Vitamin E	mg/day	≤0.25	≥250	≥50
Zinc	mg/day	≤0.5	≥100	≥50

1. Maximum values were defined using observed population intakes and dietary recommendations (11-34).
2. Child category includes ages 14 and below.

Text S4. Global Population Represented

We estimated the percent of the global population represented by our surveys using the United Nations Population Database global and national population estimates. The 188 countries for which dietary surveys were available (1,220 surveys collected between 1980 and 2015) represented 99.0% of the world's population in 2015. Surveys collected between 1980 and 1999 represented 87.7% of the world's population in 1999, while surveys collected between 2000 and 2015 represented 98.5% of the world's population in 2015.

Table S15. Characteristics of 1,220 dietary surveys in the GDD 2017 by dietary assessment method.

Survey characteristics	Overall	Dietary Recall	FFQ	DHS Questionnaire	Biomarker	Household survey
Surveys included[1], n	1,220	286	503	193	160	78
Number of countries represented, n	188	68	164	70	60	27
Percent of the global population represented in 2015, %	99.0	75.2	93.4	53.4	70.0	15.0
Demographic characteristics reported by surveys[2]						
Children/adolescents (ages 0 - 19 years), %	73.9	67.1	84.9	99.5	8.8	98.8
Age 0 - 0.49	26.3	14.0	1.4	97.9	8.8	96.2
Age 0.5 - 0.99	26.9	15.0	1.6	97.9	6.3	96.2
Age 1 - 1.9	29.3	24.1	4.0	92.2	8.8	97.4
Age 2 - 5.9	32.5	31.8	5.2	98.4	8.8	97.4
Age 6 - 10	32.0	34.6	20.9	56.0	1.3	97.4
Age 11 - 14	49.8	37.1	62.4	57.0	1.3	97.4
Age 15 - 19	61.2	50.7	82.1	57.0	1.3	98.7
Adults (ages 20+ years), %	64.5	83.9	41.9	57.0	92.5	100.0
Age 20 - 44	62.5	80.4	40.2	57.0	88.8	100.0
Age 45 - 69	61.7	77.3	40.0	57.0	89.4	100.0
Age 70+	32.8	47.9	28.2	4.1	21.9	100.0
Level of educational attainment, %	30.2	31.5	25.2	72.0	7.5	0.0
Urban vs. rural residence, %						
Both urban and rural	52.2	20.6	74.0	100.0	8.1	0.0
Urban only	4.7	9.1	5.8	0.0	1.3	0.0
Rural only	1.4	3.1	1.6	0.0	0.0	0.0
Information not available	41.7	67.1	18.7	0.0	90.6	100.0
Pregnancy/lactation status of women, %	11.2	0.3	1.0	65.3	3.1	0.0
Year of data collection, %						
1980 to 1999	36.1	41.6	20.1	27.8	71.3	66.7
2000 to 2015	63.9	58.4	79.9	72.0	28.8	33.3
Representativeness						
National, No. surveys (% of total)	880 (72.1)	182 (63.6)	401 (79.7)	193 (100.0)	26 (16.3)	78 (100.0)
Subnational, No. surveys (% of total)	207 (17.0)	44 (15.4)	66 (13.1)	0 (0.0)	97 (60.6)	0 (0.0)
Community, No. surveys (% of total)	133 (10.9)	60 (21.0)	36 (7.2)	0 (0.0)	37 (23.1)	0 (0.0)
Response rate, %						
60 to 100%	37.8	48.6	22.9	80.8	8.8	47.4
20 to 59%	3.2	7.7	1.4	0.0	0.0	12.8
< 20 %	5.7	0.0	13.5	0.0	0.0	1.3
Information not available	53.4	43.7	62.2	19.2	91.3	38.5
Sampling methodology, %						
Probability sampling, with survey weights	38.4	32.5	31.2	84.5	7.5	56.4
Probability sampling, no survey weights	32.7	26.6	54.7	15.0	1.3	21.8
Non-probability sampling	4.2	6.3	5.2	0.05	0.6	7.7
Information not available	24.7	34.6	8.9		90.6	14.1
Median (5th, 95th percentile) no. of GDD dietary food groups per survey[3]	6.0 (1.0, 30.0)	9.0 (1.0, 46.5)	3.0 (2.0, 29.9)	9.0 (2.0, 13.0)	1.0 (0.0, 1.0)	13.0 (1.0, 15.0)
Data type[4], %						
Individual-level microdata	58.6	31.1	80.5	100.0	90.6	16.7
Aggregated stratum-level distributions	41.4	68.9	19.5	0.0	9.4	83.3
Level of data collection, %						
Individual-level	92.5	95.1	99.6	99.5	100.0	5.1
Household-level	5.7	0.7	0.2	0.0	0.0	85.9
Information not available	1.7	4.2	0.2	0.5	0.0	9.0
Data source, %						

Contributed by data owner	356 (29.4)	190 (66.4)	157 (31.2)	3 (1.6)	6 (3.8)	0 (0.0)
Retrieved from public source	864 (70.6)	96 (33.6)	346 (68.8)	190 (98.4)	154 (96.3)	78 (100.0)

[1] Each survey count represents a country-specific survey-year. When data collection for a single survey was performed over multiple years, the median survey year was used (or first year if two years).

[2] Because data on children/adolescents (0-19 years), urban/rural residence, education, pregnancy/lactation, and response rate were not collected in GDD 2010 (41.7% of total surveys), these percentages may underestimate available data in these surveys. Values are shown for surveys including data on that subgroup and may sum to greater than 100% because a survey can include multiple subgroups.

[3] Based on the food groups collected in GDD 2010 (up to 21, 41.7% of surveys) and GDD 2017 (up to 54, 58.3% of surveys), not including biomarker surveys.

[4] Individual-level microdata represent individual level data in possession of the GDD. Aggregated stratum-level distributions are based on individual-level data aggregated by data owners in standardized subgroups jointly stratified by age, sex, education, and urban/rural residence, and pregnancy/lactation status, as available; and provided to the GDD including stratum-specific means, standard deviations, and percentiles of intake. Nearly all (94.2%) surveys collected in 2014-2020 (GDD 2017 round of data collection) were individual-level microdata.

Table S16. Frequency (%) of dietary factors by dietary assessment method.

No. GDD dietary factors	Single or multiple dietary recall (n=286)	FFQ (n=503)
54	0 (0.0)	0 (0.0)
≥50	5 (1.7)	7 (1.4)
≥45	18 (6.3)	12 (2.4)
≥40	21 (7.3)	14 (2.8)
≥35	27 (9.4)	17 (3.4)
≥30	28 (9.8)	26 (5.2)
≥25	37 (12.9)	38 (7.6)
≥20	64 (22.4)	47 (9.3)
≥15	104 (36.4)	61 (12.1)
≥10	135 (47.2)	82 (16.3)
≥5	192 (67.1)	164 (32.6)
≥4	196 (68.5)	186 (37.0)
≥3	205 (71.7)	353 (70.2)
≥2	210 (73.4)	492 (97.8)

All dietary factors may not have been available for 24-hr recalls or FFQs for several reasons, including: 1) the absence of reliable and updated national food composition databases or food composition tables to estimate nutrients; 2) the food, beverage, or nutrient may not be regularly consumed by the survey population; 3) the survey was identified and standardized for GDD 2010 when we were only collecting data on 21 of the 54 dietary factors; 4) the data was extracted directly from published papers and the data owners did not consent to provide more detailed individual-level microdata or aggregate stratum-level data; and 5) the data owner chose to provide a subset of available dietary data.

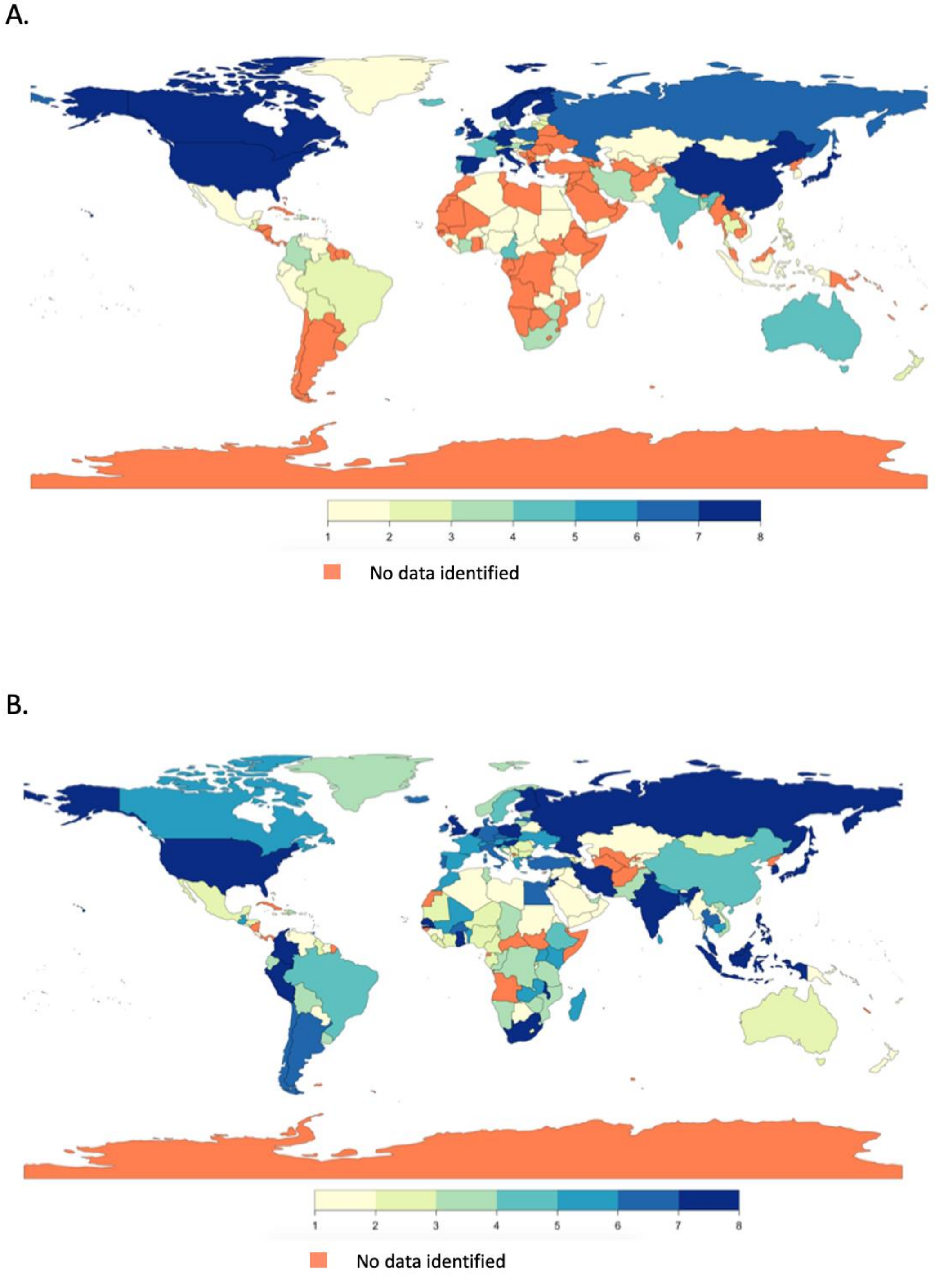

Figure S1. Geographic density of the number of dietary surveys in the GDD 2017 by country, including surveys collected between 1980 to 1999 (panel A) and 2000 to 2015 (panel B).

Table S17. Data sharing agreements for public and corresponding member (CM) surveys.

	Signed Data Sharing Agreement	No Data Sharing Agreement	Total
CM survey	135	221	356
Public survey	864	0	864

Data from surveys with signed data sharing agreements will be available for download from www.globaldietarydatabase.org.
When available, original dietary survey data are provided as both unadjusted and energy-adjusted intakes.

Miller V, *et al. BMJ Global Health* 2021; 6:e003585. doi: 10.1136/bmjgh-2020-003585

Table S18. Characteristics of surveys included in the Global Dietary Database.

Data Collection Round	Survey Name	Year Survey Began	Year Survey Ended	Country	*Residence*	*Sample Size*	*Youngest Age*	*Oldest Age*	*Male Subjects*	*Female Subjects*	*Diet Assessment Method*	*Representativeness*
GDD 2017	DHS V	2008	2009	Albania	*Both urban and rural*	*16638*	*0*	*98*	*Yes*	*Yes*	*DHS questionnair e*	*Nationally representative*
GDD 2017	Global Student Based Student Health Survey 2011	2011	2011	Algeria	*Both urban and rural*	*4451*	*11*	*16*	*Yes*	*Yes*	*FFQ*	*Nationally representative*
GDD 2010	Multi-centre study of the Mediterranean Group for the Study of Diabetes (MGSD)	1995		Algeria	*Information not reported*	*100*	*35*	*60*	*Yes*	*Yes*	*FFQ*	*Sub-nationally representative*
GDD 2010	Multi-centre study of the Mediterranean Group for the Study of Diabetes (MGSD)	1995		Algeria	*Information not reported*	*250*	*24*	*74*	*Yes*	*Yes*	*FFQ*	*Sub-nationally representative*
GDD 2010	Mesure des facteurs de risque des maladies non transmissibles dans deux zones pilotes (approche STEPWISE) Alg�rie 2003-Rapport	2003	2003	Algeria	*Information not reported*	*1000*	*25*	*64*	*Yes*	*Yes*	*FFQ*	*Nationally representative*
GDD 2010	American Samoa 1990 24 hr recall diet estimates	1990	1990	American Samoa	*Information not reported*	*250*	*25*	*55*	*Yes*	*Yes*	*Single 24-hour recall*	*Nationally representative*
GDD 2010	American Samoa NCD Risk Factors STEPS Report	2004	2004	American Samoa	*Information not reported*	*1000*	*25*	*64*	*Yes*	*Yes*	*FFQ*	*Nationally representative*
GDD 2017	Demographic and Health Survey Angola 2011	2011	2011	Angola	*Both urban and rural*	*2888*	*0*	*4*	*Yes*	*Yes*	*Biomarker*	*Nationally representative*
GDD 2017	Global Student Based Student Health Survey 2009	2009	2009	Anguilla	*Both urban and rural*	*937*	*11*	*16*	*Yes*	*Yes*	*FFQ*	*Nationally representative*
GDD 2017	Global Student Based Student Health Survey 2009	2009	2009	Antigua And Barbuda	*Both urban and rural*	*1220*	*11*	*16*	*Yes*	*Yes*	*FFQ*	*Nationally representative*
GDD 2017	Primer estudio sobre el estado nutricional y los hábitos alimentarios de la población adulta de Rosario	2012	2013	Argentina	*Only urban*	*1200*	*15*	*75*	*Yes*	*Yes*	*Single 24-hour recall*	*Locally representative*

GDD 2017	Encuesta Alimentaria y Nutricional de la Ciudad de Buenos Aires	2011	2011	Argentina	*Only urban*	*5365*	*1*	*100*	*Yes*	*Yes*	*Multiple 24-hour recall*	*Sub-nationally representative*
GDD 2017	Nutritional status of school-aged children of Buenos Aires	2005	2005	Argentina	*Only urban*	*1588*	*9*	*11*	*Yes*	*Yes*	*FFQ*	*Locally representative*
GDD 2017	Acute and long term consequences of RSV infection in children	2011	2019	Argentina	*Only urban*	*1316*	*11*	*55*	*No*	*Yes*	*FFQ*	*Sub-nationally representative*
GDD 2017	Global Student Based Student Health Survey 2007	2007	2007	Argentina	*Both urban and rural*	*1926*	*11*	*16*	*Yes*	*Yes*	*FFQ*	*Nationally representative*
GDD 2017	Global Student Based Student Health Survey 2012	2012	2012	Argentina	*Both urban and rural*	*27748*	*11*	*16*	*Yes*	*Yes*	*FFQ*	*Nationally representative*
GDD 2010	Encuesta Nacional de Nutrici�n y Salud - National Nutrition and Health Survey (ENNyS)	2004	2005	Argentina	*Information not reported*	*1000*	*20*	*49*	*No*	*Yes*	*Single 24-hour recall*	*Nationally representative*
GDD 2010	INTERSALT, Buenos Aires	1985	1987	Argentina	*Information not reported*	*25*	*20*	*59*	*Yes*	*Yes*	*Biomarker*	*Locally representative*
GDD 2017	Health Behavior in School-Aged Children 2010	2010	2010	Armenia	*Both urban and rural*	*2749*	*11*	*15*	*Yes*	*Yes*	*FFQ*	*Nationally representative*
GDD 2017	Demographic and Health Survey Armenia 2005	2005	2005	Armenia	*Both urban and rural*	*7501*	*0*	*49*	*Yes*	*Yes*	*DHS questionnaire*	*Nationally representative*
GDD 2010	Living Conditions Lifestyles and Health in the Communities of Independent States (CIS)	2001	2002	Armenia	*Information not reported*	*1000*	*18*	*99*	*Yes*	*Yes*	*FFQ*	*Nationally representative*
GDD 2010	National Nutrition Survey	1995	1995	Australia	*Information not reported*	*1000*	*0*	*100*	*Yes*	*Yes*	*Single 24-hour recall*	*Nationally representative*
GDD 2010	National Nutrition Survey	1995	1996	Australia	*Information not reported*	*1000*	*0*	*100*	*Yes*	*Yes*	*Single 24-hour recall*	*Nationally representative*
GDD 2010	Beard et al.	1995	1995	Australia	*Information not reported*	*87*	*20*	*70*	*Yes*	*Yes*	*Biomarker*	*Sub-nationally representative*
GDD 2010	Beard et al.	1989	1989	Australia	*Information not reported*	*22*	*24*	*69*	*Yes*	*Yes*	*Biomarker*	*Sub-nationally representative*
GDD 2010	Notowidjojo et al.	1992	1992	Australia	*Information not reported*	*64*	*20*	*64*	*Yes*	*Yes*	*Biomarker*	*Sub-nationally representative*
GDD 2010	Land et al.	2011	2011	Australia	*Information not reported*	*15*	*20*	*88*	*Yes*	*Yes*	*Biomarker*	*Sub-nationally representative*

GDD 2010	Charlton et al.	2007	2007	Australia	*Information not reported*	*78*	*20*	*56*	*No*	*Yes*	*Biomarker*	*Locally representative*
GDD 2010	Margerison	2003	2003	Australia	*Information not reported*	*79*	*30*	*74*	*Yes*	*Yes*	*Biomarker*	*Sub-nationally representative*
GDD 2017	Austrian Study of Nutritional Status	2010	2012	Austria	*Both urban and rural*	*889*	*6*	*81*	*Yes*	*Yes*	*FFQ*	*Nationally representative*
GDD 2017	Health Behavior in School-Aged Children 1990	1989	1990	Austria	*Both urban and rural*	*2930*	*10*	*16*	*Yes*	*Yes*	*FFQ*	*Nationally representative*
GDD 2017	Health Behavior in School-Aged Children 1994	1993	1994	Austria	*Both urban and rural*	*5217*	*10*	*16*	*Yes*	*Yes*	*FFQ*	*Nationally representative*
GDD 2017	Health Behavior in School-Aged Children 1998	1998	1998	Austria	*Both urban and rural*	*4286*	*10*	*16*	*Yes*	*Yes*	*FFQ*	*Nationally representative*
GDD 2017	Health Behavior in School-Aged Children 2002	2001	2002	Austria	*Both urban and rural*	*4366*	*10*	*16*	*Yes*	*Yes*	*FFQ*	*Nationally representative*
GDD 2017	Health Behavior in School-Aged Children 2006	2006	2006	Austria	*Both urban and rural*	*4771*	*11*	*15*	*Yes*	*Yes*	*FFQ*	*Nationally representative*
GDD 2017	Health Behavior in School-Aged Children 2010	2010	2010	Austria	*Both urban and rural*	*4983*	*11*	*15*	*Yes*	*Yes*	*FFQ*	*Nationally representative*
GDD 2010	Austrian Community Health and Nutrition Survey	1999	1999	Austria	*Information not reported*	*1000*	*0*	*100*	*Yes*	*Yes*	*Household availability/ budget survey*	*Nationally representative*
GDD 2010	Austrian Study on Nutritional Status	2005	2006	Austria	*Information not reported*	*1000*	*19*	*64*	*Yes*	*Yes*	*Single 24-hour recall*	*Nationally representative*
GDD 2010	Austrian Study on Nutritional Status 2007	2007	2007	Austria	*Information not reported*	*1000*	*7*	*100*	*Yes*	*Yes*	*Single 24-hour recall*	*Nationally representative*
GDD 2017	Demographic and Health Survey Azerbaijan 2006	2006	2006	Azerbaijan	*Both urban and rural*	*10307*	*0*	*49*	*Yes*	*Yes*	*DHS questionnaire*	*Nationally representative*
GDD 2017	Global Student Based Student Health Survey 2013	2013	2013	Bahamas	*Both urban and rural*	*1349*	*7*	*16*	*Yes*	*Yes*	*FFQ*	*Nationally representative*
GDD 2010	Health Status, Lifestyle and Nutrition Intake of Home Resident Elderly in Bahrain	1992	1992	Bahrain	*Information not reported*	*100*	*65*	*90*	*Yes*	*Yes*	*Single 24-hour recall*	*Nationally representative*

GDD 2010	Assessment of Iron Status and Dietary Intake of Pregnant Women in Bahrain	1995	1995	Bahrain	*Information not reported*	*500*	*20*	*45*	*No*	*Yes*	*Single 24-hour recall*	*Nationally representative*
GDD 2010	National Nutrition Survey	2000	2000	Bahrain	*Information not reported*	*1000*	*19*	*70*	*Yes*	*Yes*	*Single 24-hour recall*	*Nationally representative*
GDD 2017	The Health Effects of As Longitudinal Study (HEALS)	2000	2002	Bangladesh	*Only rural*	*19596*	*17*	*75*	*Yes*	*Yes*	*FFQ*	*Locally representative*
GDD 2017	HarvestPlus Baseline Dietary Survey	2007	2008	Bangladesh	*Only rural*	*1839*	*2*	*70*	*Yes*	*Yes*	*Multiple 24-hour recall*	*Locally representative*
GDD 2017	Demographic and Health Survey Bangladesh IV 1999-2000	1999	2000	Bangladesh	*Both urban and rural*	*4917*	*0*	*4*	*Yes*	*Yes*	*DHS questionnaire*	*Nationally representative*
GDD 2017	Demographic and Health Survey Bangladesh IV 2004	2004	2004	Bangladesh	*Both urban and rural*	*6365*	*0*	*4*	*Yes*	*Yes*	*DHS questionnaire*	*Nationally representative*
GDD 2017	Demographic and Health Survey Bangladesh V 2007	2007	2007	Bangladesh	*Both urban and rural*	*4201*	*0*	*4*	*Yes*	*Yes*	*DHS questionnaire*	*Nationally representative*
GDD 2017	Demographic and Health Survey Bangladesh VI 2011	2011	2011	Bangladesh	*Both urban and rural*	*11065*	*0*	*49*	*Yes*	*Yes*	*DHS questionnaire*	*Nationally representative*
GDD 2017	Demographic and Health Survey Bangladesh VII 2014	2014	2014	Bangladesh	*Both urban and rural*	*3623*	*0*	*4*	*Yes*	*Yes*	*DHS questionnaire*	*Nationally representative*
GDD 2017	Bangladesh World Health Survey 2003	2003	2003	Bangladesh	*Both urban and rural*	*5512*	*18*	*110*	*Yes*	*Yes*	*FFQ*	*Nationally representative*
GDD 2017	Demographic and Health Survey Bangladesh 1993	1993	1994	Bangladesh	*Both urban and rural*	*3014*	*0*	*2*	*Yes*	*Yes*	*DHS questionnaire*	*Nationally representative*
GDD 2017	Demographic and Health Survey Bangladesh 1996	1996	1997	Bangladesh	*Both urban and rural*	*4313*	*0*	*4*	*Yes*	*Yes*	*DHS questionnaire*	*Nationally representative*
GDD 2010	Dietary Diversity as a Measure of the Micronutrient Adequacy of Women's Diets: Results from Rural Bangladesh Site	1996	1996	Bangladesh	*Information not reported*	*250*	*15*	*49*	*No*	*Yes*	*Single 24-hour recall*	*Sub-nationally representative*
GDD 2017	Global Student Based Student Health Survey 2011	2011	2011	Barbados	*Both urban and rural*	*1618*	*11*	*16*	*Yes*	*Yes*	*FFQ*	*Nationally representative*

Miller V, *et al. BMJ Global Health* 2021; 6:e003585. doi: 10.1136/bmjgh-2020-003585

GDD 2010	Identifying new genetic and obesity-related factors contributing to prostate and breast cancer risk in persons of African descent	2004	2007	Barbados	*Information not reported*	*250*	*31*	*88*	*Yes*	*Yes*	*FFQ*	*Nationally representative*
GDD 2010	Barbados STEPS Survey 2007	2007	2007	Barbados	*Information not reported*	*1000*	*25*	*100*	*Yes*	*Yes*	*FFQ*	*Nationally representative*
GDD 2010	Cooper et al.	1991	1994	Barbados	*Information not reported*	*813*	*25*	*74*	*Yes*	*Yes*	*Biomarker*	*Sub-nationally representative*
GDD 2010	Living Conditions Lifestyles and Health	2001	2002	Belarus	*Information not reported*	*1000*	*18*	*99*	*Yes*	*Yes*	*FFQ*	*Nationally representative*
GDD 2017	Flanders preschool dietary survey	2002	2003	Belgium	*Both urban and rural*	*1983*	*3*	*7*	*Yes*	*Yes*	*Multiple 24-hour recall*	*Sub-nationally representative*
GDD 2017	Health Behavior in School-Aged Children 1986	1985	1986	Belgium	*Both urban and rural*	*3575*	*11*	*16*	*Yes*	*Yes*	*FFQ*	*Nationally representative*
GDD 2017	Health Behavior in School-Aged Children 1990	1989	1990	Belgium	*Both urban and rural*	*3007*	*11*	*16*	*Yes*	*Yes*	*FFQ*	*Nationally representative*
GDD 2017	Health Behavior in School-Aged Children 1994	1993	1994	Belgium	*Both urban and rural*	*5196*	*11*	*16*	*Yes*	*Yes*	*FFQ*	*Sub-nationally representative*
GDD 2017	Health Behavior in School-Aged Children 1994	1993	1994	Belgium	*Both urban and rural*	*4506*	*11*	*16*	*Yes*	*Yes*	*FFQ*	*Sub-nationally representative*
GDD 2017	Health Behavior in School-Aged Children 1998	1998	1998	Belgium	*Both urban and rural*	*2505*	*11*	*16*	*Yes*	*Yes*	*FFQ*	*Sub-nationally representative*
GDD 2017	Health Behavior in School-Aged Children 1998	1998	1998	Belgium	*Both urban and rural*	*4824*	*11*	*16*	*Yes*	*Yes*	*FFQ*	*Sub-nationally representative*
GDD 2017	Health Behavior in School-Aged Children 2002	2001	2002	Belgium	*Both urban and rural*	*10612*	*11*	*16*	*Yes*	*Yes*	*FFQ*	*Nationally representative*
GDD 2017	Health Behavior in School-Aged Children 2006	2006	2006	Belgium	*Both urban and rural*	*8757*	*11*	*15*	*Yes*	*Yes*	*FFQ*	*Nationally representative*
GDD 2017	Health Behavior in School-Aged Children 2010	2010	2010	Belgium	*Both urban and rural*	*8159*	*11*	*15*	*Yes*	*Yes*	*FFQ*	*Nationally representative*
GDD 2010	Belgian Community Health and Nutrition Survey	1987	1988	Belgium	*Information not reported*	*1000*	*0*	*100*	*Yes*	*Yes*	*Household availability/*	*Nationally representative*

											budget survey	
GDD 2010	Belgian Community Health and Nutrition Survey	1996	1997	Belgium	*Information not reported*	*1000*	*0*	*100*	*Yes*	*Yes*	*Household availability/ budget survey*	*Nationally representative*
GDD 2010	Belgian Community Health and Nutrition Survey	1999	1999	Belgium	*Information not reported*	*1000*	*0*	*100*	*Yes*	*Yes*	*Household availability/ budget survey*	*Nationally representative*
GDD 2010	Belgian National Food Consumption Survey	2004	2004	Belgium	*Information not reported*	*1000*	*15*	*100*	*Yes*	*Yes*	*Multiple 24-hour recall*	*Nationally representative*
GDD 2010	Belgian National Food Consumption Survey	2004	2004	Belgium	*Information not reported*	*1000*	*15*	*100*	*Yes*	*Yes*	*Multiple 24-hour recall*	*Nationally representative*
GDD 2010	TRANSFAIR study	1991	1992	Belgium	*Information not reported*	*250*	*18*	*65*	*Yes*	*Yes*	*Single 24-hour recall*	*Nationally representative*
GDD 2010	Staessen et al.	1980	1981	Belgium	*Information not reported*	*273*	*20*	*88*	*Yes*	*Yes*	*Biomarker*	*Sub-nationally representative*
GDD 2010	INTERSALT, Charleroi	1985	1987	Belgium	*Information not reported*	*11*	*20*	*59*	*Yes*	*Yes*	*Biomarker*	*Sub-nationally representative*
GDD 2010	INTERSALT, Ghent	1985	1987	Belgium	*Information not reported*	*25*	*20*	*59*	*Yes*	*Yes*	*Biomarker*	*Sub-nationally representative*
GDD 2010	Vandevijvere et al.	2007	2008	Belgium	*Information not reported*	*60*	*25*	*65*	*Yes*	*Yes*	*Biomarker*	*Sub-nationally representative*
GDD 2010	Vandevijvere et al.	2009	2009	Belgium	*Information not reported*	*66*	*25*	*65*	*Yes*	*Yes*	*Biomarker*	*Sub-nationally representative*
GDD 2010	Zhang et al.	1997	1997	Belgium	*Information not reported*	*2124*	*25*	*74*	*Yes*	*Yes*	*Biomarker*	*Nationally representative*
GDD 2010	Zhang et al.	1997	1997	Belgium	*Information not reported*	*2124*	*25*	*74*	*Yes*	*Yes*	*Single 24-hour recall*	*Sub-nationally representative*
GDD 2017	Global Student Based Student Health Survey 2011	2011	2011	Belize	*Both urban and rural*	*2084*	*11*	*16*	*Yes*	*Yes*	*FFQ*	*Nationally representative*
GDD 2010	Simmons et al.	1980	1980	Belize	*Information not reported*	*392*	*20*	*94*	*Yes*	*No*	*Biomarker*	*Locally representative*
GDD 2017	Global Student Based Student Health Survey 2009	2009	2009	Benin	*Both urban and rural*	*2664*	*11*	*16*	*Yes*	*Yes*	*FFQ*	*Nationally representative*
GDD 2017	Demographic and Health Survey Benin 1996	1996	1996	Benin	*Both urban and rural*	*2497*	*0*	*2*	*Yes*	*Yes*	*DHS questionnair e*	*Nationally representative*

Miller V, *et al. BMJ Global Health* 2021; 6:e003585. doi: 10.1136/bmjgh-2020-003585

GDD 2017	Demographic and Health Survey Benin 2001	2001	2001	Benin	*Both urban and rural*	*7447*	*0*	*49*	*Yes*	*Yes*	*DHS questionnaire*	*Nationally representative*
GDD 2017	Demographic and Health Survey Benin 2006	2006	2006	Benin	*Both urban and rural*	*18327*	*0*	*49*	*Yes*	*Yes*	*DHS questionnaire*	*Nationally representative*
GDD 2017	Demographic and Health Survey Benin 2011-2012	2011	2012	Benin	*Both urban and rural*	*17646*	*0*	*49*	*Yes*	*Yes*	*DHS questionnaire*	*Nationally representative*
GDD 2010	STEP Benin	2007	2007	Benin	*Information not reported*	*1000*	*25*	*64*	*Yes*	*Yes*	*FFQ*	*Sub-nationally representative*
GDD 2010	RAPPORT FINAL DE L�ENQUETE STEPS AU BENIN	2008	2008	Benin	*Information not reported*	*1000*	*25*	*64*	*Yes*	*Yes*	*FFQ*	*Nationally representative*
GDD 2010	Melse-Boonstra et al. (2 of 4)	1996	1996	Benin	*Information not reported*	*13*	*22*	*55*	*No*	*Yes*	*Biomarker*	*Locally representative*
GDD 2010	Report on 2007 STEPS Survey for Risk Factors and Prevalence of Noncommunicable Diseases in Thimphu	2007	2007	Bhutan	*Information not reported*	*1000*	*25*	*74*	*Yes*	*Yes*	*FFQ*	*Sub-nationally representative*
GDD 2017	Cardiovascular and metabolic syndrome risk assessment of Bolivian school children and adolescents: Relationships to obesity, diabetes, income, food intake and physical activity	2005	2007	Bolivia	*Both urban and rural*	*3445*	*12*	*18*	*Yes*	*Yes*	*FFQ*	*Nationally representative*
GDD 2017	Global Student Based Student Health Survey 2012	2012	2012	Bolivia	*Both urban and rural*	*3468*	*11*	*16*	*Yes*	*Yes*	*FFQ*	*Nationally representative*
GDD 2017	Demographic and Health Survey Bolivia 2003	2003	2003	Bolivia	*Both urban and rural*	*15619*	*0*	*49*	*Yes*	*Yes*	*DHS questionnaire*	*Nationally representative*
GDD 2017	Demographic and Health Survey Bolivia 2008	2008	2008	Bolivia	*Both urban and rural*	*15569*	*0*	*49*	*Yes*	*Yes*	*DHS questionnaire*	*Nationally representative*
GDD 2017	Demographic and Health Survey Bolivia 1989	1989	1989	Bolivia	*Both urban and rural*	*1596*	*0*	*4*	*Yes*	*Yes*	*DHS questionnaire*	*Nationally representative*
GDD 2017	Demographic and Health Survey Bolivia 1994	1994	1994	Bolivia	*Both urban and rural*	*2856*	*0*	*2*	*Yes*	*Yes*	*DHS questionnaire*	*Nationally representative*

GDD 2017	Demographic and Health Survey Bolivia 1998	1998	1998	Bolivia	*Both urban and rural*	*8709*	*0*	*49*	*Yes*	*Yes*	*DHS questionnaire*	*Nationally representative*
GDD 2017	World Health Survey 2003	2003	2003	Bosnia And Herzegovina	*Both urban and rural*	*951*	*18*	*84*	*Yes*	*Yes*	*FFQ*	*Nationally representative*
GDD 2010	Non- communicable Disease Risk Factor Survey Federation of Bosnia and Herzegovina	2002	2002	Bosnia And Herzegovina	*Information not reported*	*1000*	*25*	*64*	*Yes*	*Yes*	*FFQ*	*Sub-nationally representative*
GDD 2017	Global Student Based Student Health Survey 2005	2005	2005	Botswana	*Both urban and rural*	*2157*	*11*	*16*	*Yes*	*Yes*	*FFQ*	*Nationally representative*
GDD 2010	Republic of Botswana Chronic Disease Risk Factor Surveillance Report 2007 (STEPS)	2007	2007	Botswana	*Information not reported*	*1000*	*25*	*64*	*Yes*	*Yes*	*FFQ*	*Nationally representative*
GDD 2017	Health Survey of São Paulo	2008	2009	Brazil	*Only urban*	*1662*	*12*	*97*	*Yes*	*Yes*	*Multiple 24-hour recall*	*Sub-nationally representative*
GDD 2017	Demographic and Health Survey Brasil 1996	1996	1996	Brazil	*Both urban and rural*	*1706*	*11*	*16*	*Yes*	*Yes*	*DHS questionnaire*	*Nationally representative*
GDD 2017	Demographic and Health Survey Brasil 1991	1991	1991	Brazil	*Both urban and rural*	*457*	*0*	*4*	*Yes*	*Yes*	*DHS questionnaire*	*Nationally representative*
GDD 2017	World Health Survey 2003	2003	2003	Brazil	*Both urban and rural*	*2608*	*18*	*95*	*Yes*	*Yes*	*FFQ*	*Nationally representative*
GDD 2017	Demographic and Health Survey Brazil 1986	1986	1986	Brazil	*Both urban and rural*	*536*	*0*	*4*	*Yes*	*Yes*	*DHS questionnaire*	*Nationally representative*
GDD 2010	Household Health Survey (ISA-SP)	2003	2003	Brazil	*Information not reported*	*1000*	*20*	*100*	*Yes*	*Yes*	*Single 24-hour recall*	*Sub-nationally representative*
GDD 2010	Consumer Expenditure Survey	2008	2009	Brazil	*Information not reported*	*1484*	*10*	*100*	*Yes*	*Yes*	*Multiple 24-hour recall*	*Nationally representative*
GDD 2010	Sarno	2002	2003	Brazil	*Information not reported*	*48000*	*20*	*79*	*Yes*	*Yes*	*Household availability/ budget survey*	*Nationally representative*
GDD 2017	Global Student Based Student Health Survey 2014	2014	2014	Brunei Darussalam	*Both urban and rural*	*2586*	*7*	*16*	*Yes*	*Yes*	*FFQ*	*Nationally representative*
GDD 2010	National Nutritional Status Survey 1997	1996	1997	Brunei Darussalam	*Information not reported*	*1000*	*0*	*100*	*Yes*	*Yes*	*Single 24-hour recall*	*Nationally representative*

GDD 2017	Health Behavior in School-Aged Children 2006	2006	2006	Bulgaria	*Both urban and rural*	*4798*	*11*	*15*	*Yes*	*Yes*	*FFQ*	*Nationally representative*
GDD 2010	Household Budgets in Republic of Bulgaria	1998	1998	Bulgaria	*Information not reported*	*1000*	*20*	*85*	*Yes*	*Yes*	*Multiple 24-hour recall*	*Nationally representative*
GDD 2010	National Nutrition Survey	2004	2004	Bulgaria	*Information not reported*	*500*	*16*	*64*	*Yes*	*Yes*	*Single 24-hour recall*	*Nationally representative*
GDD 2010	National Nutrition Survey	2004	2004	Bulgaria	*Information not reported*	*500*	*20*	*100*	*Yes*	*Yes*	*Single 24-hour recall*	*Nationally representative*
GDD 2010	Powles et al.	1999	2000	Bulgaria	*Information not reported*	*30*	*45*	*74*	*Yes*	*Yes*	*Biomarker*	*Sub-nationally representative*
GDD 2017	Nutrition transition and double burden of malnutrition among adults in Ouagadougou, Burkina Faso (West Africa)	2010	2010	Burkina Faso	*Only urban*	*310*	*25*	*60*	*Yes*	*Yes*	*Multiple 24-hour recall*	*Locally representative*
GDD 2017	Food Consumption and Iron Status Survey in Two Provinces of Rural Burkina Faso	2010	2010	Burkina Faso	*Only rural*	*2628*	*3*	*55*	*Yes*	*Yes*	*Multiple 24-hour recall*	*Sub-nationally representative*
GDD 2017	World Health Survey 2003	2003	2003	Burkina Faso	*Both urban and rural*	*4464*	*18*	*112*	*Yes*	*Yes*	*FFQ*	*Nationally representative*
GDD 2017	Demographic and Health Survey Burkina Faso 1993	1993	1994	Burkina Faso	*Both urban and rural*	*2357*	*0*	*4*	*Yes*	*Yes*	*DHS questionnaire*	*Nationally representative*
GDD 2017	Demographic and Health Survey Burkina Faso 2003	2003	2003	Burkina Faso	*Both urban and rural*	*13502*	*0*	*49*	*Yes*	*Yes*	*DHS questionnaire*	*Nationally representative*
GDD 2017	Demographic and Health Survey Burkina Faso 2010	2010	2011	Burkina Faso	*Both urban and rural*	*26818*	*0*	*59*	*Yes*	*Yes*	*DHS questionnaire*	*Nationally representative*
GDD 2017	Demographic and Health Survey Burkina Faso 1999	1998	1999	Burkina Faso	*Both urban and rural*	*2473*	*0*	*4*	*Yes*	*Yes*	*DHS questionnaire*	*Nationally representative*
GDD 2017	Demographic and Health Survey Burkina Faso 2014	2014	2014	Burkina Faso	*Both urban and rural*	*6155*	*1*	*5*	*Yes*	*Yes*	*Biomarker*	*Nationally representative*

GDD 2010	Dietary Diversity as a Measure of the Micronutrient Adequacy of Women's Diets: Results from Ouagadougou, Burkina Faso Site	2006	2006	Burkina Faso	*Information not reported*	*100*	*17*	*49*	*No*	*Yes*	*Multiple 24-hour recall*	*Sub-nationally representative*
GDD 2017	Deomgraphic and Health Suvey Burundi 2012	2012	2013	Burundi	*Both urban and rural*	*3754*	*1*	*5*	*Yes*	*Yes*	*Biomarker*	*Nationally representative*
GDD 2017	Demographic and Health Survey Burundi 2010	2010	2011	Burundi	*Both urban and rural*	*14636*	*0*	*59*	*Yes*	*Yes*	*DHS questionnaire*	*Nationally representative*
GDD 2017	Demographic and Health Survey Burundi 1987	1987	1987	Burundi	*Both urban and rural*	*1539*	*0*	*4*	*Yes*	*Yes*	*DHS questionnaire*	*Nationally representative*
GDD 2017	Global Student Based Student Health Survey 2013	2013	2013	Cambodia	*Both urban and rural*	*3792*	*7*	*16*	*Yes*	*Yes*	*FFQ*	*Nationally representative*
GDD 2017	Demographic and Health Survey Cambodia 2000	2000	2000	Cambodia	*Both urban and rural*	*11392*	*0*	*49*	*Yes*	*Yes*	*DHS questionnaire*	*Nationally representative*
GDD 2017	Demographic and Health Survey Cambodia 2005	2005	2006	Cambodia	*Both urban and rural*	*14054*	*0*	*49*	*Yes*	*Yes*	*DHS questionnaire*	*Nationally representative*
GDD 2017	Demographic and Health Survey Cambodia 2010	2010	2011	Cambodia	*Both urban and rural*	*15194*	*0*	*49*	*Yes*	*Yes*	*DHS questionnaire*	*Nationally representative*
GDD 2017	Demographic and Health Survey Cambodia 2014	2014	2014	Cambodia	*Both urban and rural*	*17098*	*0*	*49*	*Yes*	*Yes*	*DHS questionnaire*	*Nationally representative*
GDD 2010	STEP Cambodia			Cambodia	*Information not reported*	*1000*	*25*	*64*	*Yes*	*Yes*	*FFQ*	*Sub-nationally representative*
GDD 2017	Nutrition in African Population-Urban	1993	1996	Cameroon	*Only urban*	*991*	*24*	*74*	*Yes*	*Yes*	*FFQ*	*Sub-nationally representative*
GDD 2017	Nutrition in African Population-Rural	1993	1996	Cameroon	*Only rural*	*658*	*24*	*74*	*Yes*	*Yes*	*FFQ*	*Sub-nationally representative*
GDD 2017	Demographic and Health Survey Cameroon 2004	2004	2004	Cameroon	*Both urban and rural*	*16581*	*0*	*59*	*Yes*	*Yes*	*DHS questionnaire*	*Nationally representative*
GDD 2017	Demographic and Health Survey Cameroon 2011	2011	2011	Cameroon	*Both urban and rural*	*17442*	*0*	*49*	*Yes*	*Yes*	*DHS questionnaire*	*Nationally representative*

GDD 2017	Demographic and Health Survey Cameroon 1991	1991	1991	Cameroon	*Both urban and rural*	*970*	*0*	*4*	*Yes*	*Yes*	*DHS questionnaire*	*Nationally representative*
GDD 2017	Demographic and Health Survey Cameroon 1998	1998	1998	Cameroon	*Both urban and rural*	*1826*	*0*	*2*	*Yes*	*Yes*	*DHS questionnaire*	*Nationally representative*
GDD 2010	Cameroon Burden of Diabetes Project Baseline Survey Report Summary	2003	2003	Cameroon	*Information not reported*	*1000*	*15*	*100*	*Yes*	*Yes*	*FFQ*	*Sub-nationally representative*
GDD 2010	Cooper et al.	1991	1994	Cameroon	*Information not reported*	*2828*	*25*	*74*	*Yes*	*Yes*	*Biomarker*	*Sub-nationally representative*
GDD 2017	Health Behavior in School-Aged Children 1994	1993	1994	Canada	*Both urban and rural*	*6758*	*11*	*17*	*Yes*	*Yes*	*FFQ*	*Nationally representative*
GDD 2017	Health Behavior in School-Aged Children 1998	1998	1998	Canada	*Both urban and rural*	*6567*	*11*	*16*	*Yes*	*Yes*	*FFQ*	*Nationally representative*
GDD 2017	Health Behavior in School-Aged Children 2002	2001	2002	Canada	*Both urban and rural*	*4361*	*11*	*16*	*Yes*	*Yes*	*FFQ*	*Nationally representative*
GDD 2017	Health Behavior in School-Aged Children 2006	2006	2006	Canada	*Both urban and rural*	*5772*	*11*	*15*	*Yes*	*Yes*	*FFQ*	*Nationally representative*
GDD 2017	Health Behavior in School-Aged Children 2010	2010	2010	Canada	*Both urban and rural*	*15632*	*11*	*15*	*Yes*	*Yes*	*FFQ*	*Nationally representative*
GDD 2017	Health Behavior in School-Aged Children 1990	1989	1990	Canada	*Both urban and rural*	*5551*	*11*	*17*	*Yes*	*Yes*	*FFQ*	*Nationally representative*
GDD 2010	Canadian Community Health and Nutrition Survey	2004	2004	Canada	*Information not reported*	*1000*	*0*	*101*	*Yes*	*Yes*	*Multiple 24-hour recall*	*Nationally representative*
GDD 2010	World-wide consumption of trans fatty acids	1981	1981	Canada	*Information not reported*		*0*	*100*	*Yes*	*Yes*	*Household availability/ budget survey*	*Nationally representative*
GDD 2010	World-wide consumption of trans fatty acids	1981	1981	Canada	*Information not reported*		*0*	*100*	*Yes*	*Yes*	*Single 24-hour recall*	*Nationally representative*
GDD 2010	World-wide consumption of trans fatty acids	1995	1995	Canada	*Information not reported*		*0*	*100*	*Yes*	*Yes*	*Single 24-hour recall*	*Nationally representative*

GDD 2010	World-wide consumption of trans fatty acids	1995	1995	Canada	*Information not reported*		*0*	*100*	*Yes*	*Yes*	*Single 24-hour recall*	*Nationally representative*
GDD 2010	World-wide consumption of trans fatty acids	1999	1999	Canada	*Information not reported*		*0*	*100*	*Yes*	*Yes*	*Single 24-hour recall*	*Nationally representative*
GDD 2010	Shi et al.	2004	2004	Canada	*Information not reported*	*1402*	*30*	*79*	*Yes*	*Yes*	*Single 24-hour recall*	*Nationally representative*
GDD 2010	Garriguet	2004	2004	Canada	*Information not reported*	*681*	*20*	*79*	*Yes*	*Yes*	*Single 24-hour recall*	*Nationally representative*
GDD 2010	INTERSALT, Labrador	1985	1987	Canada	*Information not reported*	*23*	*20*	*59*	*Yes*	*Yes*	*Biomarker*	*Locally representative*
GDD 2010	INTERSALT, St. Johns	1985	1987	Canada	*Information not reported*	*25*	*20*	*59*	*Yes*	*Yes*	*Biomarker*	*Sub-nationally representative*
GDD 2010	Cape Verde STEPS Survey 2007	2007	2007	Cape Verde	*Information not reported*	*1000*	*25*	*64*	*Yes*	*Yes*	*FFQ*	*Nationally representative*
GDD 2017	Global Student Based Student Health Survey 2007	2007	2007	Cayman Islands	*Only urban*	*1288*	*11*	*16*	*Yes*	*Yes*	*FFQ*	*Nationally representative*
GDD 2017	Demographic and Health Survey Central African Republic 1994	1994	1995	Central African Republic	*Both urban and rural*	*2490*	*0*	*2*	*Yes*	*Yes*	*DHS questionnaire*	*Nationally representative*
GDD 2017	World Health Survey 2003	2003	2003	Chad	*Both urban and rural*	*3516*	*17*	*106*	*Yes*	*Yes*	*FFQ*	*Nationally representative*
GDD 2017	Demographic and Health Survey Chad 2004	2004	2004	Chad	*Both urban and rural*	*3178*	*0*	*4*	*Yes*	*Yes*	*DHS questionnaire*	*Nationally representative*
GDD 2017	Demographic and Health Survey Chad 1996	1996	1997	Chad	*Both urban and rural*	*6145*	*0*	*4*	*Yes*	*Yes*	*DHS questionnaire*	*Nationally representative*
GDD 2017	Demographic and Health Survey Chad 2014	2014	2015	Chad	*Both urban and rural*	*16846*	*0*	*4*	*Yes*	*Yes*	*DHS questionnaire*	*Nationally representative*
GDD 2010	STEP Chad	2008	2008	Chad	*Information not reported*	*1000*	*25*	*64*	*Yes*	*Yes*	*FFQ*	*Sub-nationally representative*
GDD 2017	Estado nutricional, consumo de alimentos y actividad física en escolares mujeres de diferente nivel socioeconómico de Santiago de Chile	2003	2004	Chile	*Only urban*	*202*	*8*	*14*	*No*	*Yes*	*FFQ*	*Sub-nationally representative*

GDD 2017	Estado nutricional, consumo de alimentos y actividad física en escolares mujeres de diferente nivel socioeconómico de Santiago de Chile	2003	2004	Chile	*Only urban*	*356*	*8*	*13*	*No*	*Yes*	*FFQ*	*Sub-nationally representative*
GDD 2017	Encuesta de Nutrientes en universitarios chilenos	2012	2012	Chile	*Only urban*	*326*	*18*	*30*	*Yes*	*Yes*	*FFQ*	*Locally representative*
GDD 2017	Food and Elderly	2014	2014	Chile	*Only urban*	*1450*	*60*	*100*	*Yes*	*Yes*	*FFQ*	*Sub-nationally representative*
GDD 2017	Global Student Based Student Health Survey 2004	2004	2004	Chile	*Both urban and rural*	*8083*	*11*	*16*	*Yes*	*Yes*	*FFQ*	*Nationally representative*
GDD 2017	Global Student Based Student Health Survey 2013	2013	2013	Chile	*Both urban and rural*	*2030*	*7*	*16*	*Yes*	*Yes*	*FFQ*	*Nationally representative*
GDD 2017	Global Student Based Student Health Survey 2005	2005	2005	Chile	*Both urban and rural*	*7199*	*11*	*16*	*Yes*	*Yes*	*FFQ*	*Nationally representative*
GDD 2017	Global Student Based Student Health Survey 2003	2003	2003	China	*Only urban*	*8846*	*11*	*16*	*Yes*	*Yes*	*FFQ*	*Nationally representative*
GDD 2017	Study of Global Ageing and Adult Health	2007	2013	China	*Both urban and rural*	*14168*	*18*	*99*	*Yes*	*Yes*	*FFQ*	*Nationally representative*
GDD 2017	World Health Survey 2003	2003	2003	China	*Both urban and rural*	*3722*	*18*	*98*	*Yes*	*Yes*	*FFQ*	*Nationally representative*
GDD 2010	2002 China National Nutrition and Health Survey	2002	2002	China	*Information not reported*	*1346*	*1*	*101*	*Yes*	*Yes*	*Multiple 24-hour recall*	*Nationally representative*
GDD 2010	Zhao, L.	2002	2002	China	*Information not reported*	*222104*	*20*	*20*	*Yes*	*No*	*Multiple 24-hour recall*	*Nationally representative*
GDD 2010	Zhou BF et al. (1 of 9)	1982	1984	China	*Information not reported*	*342*	*35*	*59*	*Yes*	*No*	*Multiple 24-hour recall*	*Locally representative*
GDD 2010	Zhao GS et al.(1 of 3)	1982	1984	China	*Information not reported*	*92*	*40*	*59*	*Yes*	*No*	*Biomarker*	*Locally representative*
GDD 2010	INTERSALT, Beijing	1985	1987	China	*Information not reported*	*25*	*20*	*59*	*Yes*	*Yes*	*Biomarker*	*Sub-nationally representative*
GDD 2010	INTERSALT, Tianjin	1985	1987	China	*Information not reported*	*25*	*20*	*59*	*Yes*	*Yes*	*Biomarker*	*Sub-nationally representative*
GDD 2010	INTERSALT, Nanning	1985	1987	China	*Information not reported*	*25*	*20*	*59*	*Yes*	*Yes*	*Biomarker*	*Sub-nationally representative*
GDD 2010	Nan et al.	1992	1992	China	*Information not reported*	*1133*	*20*	*64*	*Yes*	*Yes*	*Multiple 24-hour recall*	*Sub-nationally representative*

GDD 2010	Liu et al.	1985	1999	China	*Information not reported*	*403*	*48*	*56*	*Yes*	*Yes*	*Biomarker*	*Nationally representative*
GDD 2010	INTERMAP, Beijing	1997	1997	China	*Information not reported*	*67*	*40*	*59*	*Yes*	*Yes*	*Biomarker*	*Sub-nationally representative*
GDD 2010	INTERMAP, Guangxi	1997	1998	China	*Information not reported*	*70*	*40*	*59*	*Yes*	*Yes*	*Biomarker*	*Sub-nationally representative*
GDD 2010	INTERMAP, Shanxi	1997	1998	China	*Information not reported*	*73*	*40*	*59*	*Yes*	*Yes*	*Biomarker*	*Sub-nationally representative*
GDD 2010	Zhou BF et al. (2 of 9)	1982	1984	China	*Information not reported*	*124*	*35*	*59*	*Yes*	*No*	*Multiple 24-hour recall*	*Locally representative*
GDD 2010	Zhou BF et al. (3 of 9)	1982	1984	China	*Information not reported*	*51*	*35*	*59*	*Yes*	*No*	*Multiple 24-hour recall*	*Locally representative*
GDD 2010	Zhou BF et al. (4 of 9)	1982	1984	China	*Information not reported*	*119*	*35*	*59*	*Yes*	*No*	*Multiple 24-hour recall*	*Locally representative*
GDD 2010	Zhou BF et al. (5 of 9)	1982	1984	China	*Information not reported*	*181*	*35*	*59*	*Yes*	*No*	*Multiple 24-hour recall*	*Locally representative*
GDD 2010	Zhou BF et al. (6 of 9)	1982	1984	China	*Information not reported*	*215*	*35*	*59*	*Yes*	*No*	*Multiple 24-hour recall*	*Locally representative*
GDD 2010	Zhou BF et al. (7 of 9)	1982	1984	China	*Information not reported*	*85*	*35*	*59*	*Yes*	*No*	*Multiple 24-hour recall*	*Locally representative*
GDD 2010	Zhou BF et al. (8 of 9)	1982	1984	China	*Information not reported*	*212*	*35*	*59*	*Yes*	*No*	*Multiple 24-hour recall*	*Locally representative*
GDD 2010	Zhou BF et al. (9 of 9)	1982	1984	China	*Information not reported*	*195*	*35*	*59*	*Yes*	*No*	*Multiple 24-hour recall*	*Locally representative*
GDD 2010	Zhao GS et al. (2 of 3)	1982	1984	China	*Information not reported*	*82*	*40*	*59*	*Yes*	*No*	*Biomarker*	*Locally representative*
GDD 2010	Zhao GS et al.(3 of 3)	1982	1984	China	*Information not reported*	*83*	*40*	*59*	*Yes*	*No*	*Biomarker*	*Locally representative*
GDD 2010	INTERMAP, Beijing	1997	1997	China	*Information not reported*	*67*	*40*	*59*	*Yes*	*Yes*	*Single 24-hour recall*	*Sub-nationally representative*
GDD 2010	INTERMAP, Guangxi	1997	1998	China	*Information not reported*	*70*	*40*	*59*	*Yes*	*Yes*	*Single 24-hour recall*	*Sub-nationally representative*
GDD 2010	INTERMAP, Shanxi	1997	1997	China	*Information not reported*	*73*	*40*	*59*	*Yes*	*Yes*	*Single 24-hour recall*	*Sub-nationally representative*
GDD 2017	Situación de salud y perfil metabólico en población menor de 18 años residente en Santander, Colombia (The SALUS Study)	2013	2014	Colombia	*Both urban and rural*	*3697*	*0*	*17*	*Yes*	*Yes*	*FFQ*	*Sub-nationally representative*
GDD 2017	CASE CONTROL STUDY TO IDENTIFY RISK FACTORS FOR PREECLAMPSIA	2006	2009	Colombia	*Both urban and rural*	*383*	*12*	*45*	*Yes*	*Yes*	*FFQ*	*Nationally representative*

GDD 2017	Associated factors to dietary intake of fruits and vegetables in Bucaramanga, Colombia	2006	2007	Colombia	*Only urban*	*317*	*18*	*90*	*Yes*	*Yes*	*Single 24-hour recall*	*Locally representative*
GDD 2017	Effects of environmental exposure to lead and cadmium in the occurrence of low birth weight (LBW) and intrauterine growth restriction in a cohort of pregnant women Cali-Colombia 2009-2011	2010	2012	Colombia	*Only urban*	*380*	*18*	*35*	*No*	*Yes*	*Single 24-hour recall*	*Locally representative*
GDD 2017	Urban Schooled Children -IFRECNTEC Study	1997	2000	Colombia	*Only urban*	*2807*	*6*	*19*	*Yes*	*Yes*	*Single 24-hour recall*	*Locally representative*
GDD 2017	Iron and cardiovascular risk project	2009	2011	Colombia	*Only urban*	*245*	*25*	*68*	*Yes*	*Yes*	*Biomarker*	*Locally representative*
GDD 2017	Improving the nutritional status of preschool children assessing individual response to different types of interventions in nutrient intake	2005	2007	Colombia	*Only urban*	*520*	*1*	*6*	*Yes*	*Yes*	*Biomarker*	*Locally representative*
GDD 2017	Global Student Based Student Health Survey 2007	2007	2007	Colombia	*Only urban*	*9823*	*11*	*16*	*Yes*	*Yes*	*FFQ*	*Nationally representative*
GDD 2017	Demographic and Health Survey Colombia 1995	1995	1995	Colombia	*Both urban and rural*	*3671*	*0*	*4*	*Yes*	*Yes*	*DHS questionnair e*	*Nationally representative*
GDD 2017	Demographic and Health Survey Colombia 2000	2000	2000	Colombia	*Both urban and rural*	*3380*	*0*	*4*	*Yes*	*Yes*	*DHS questionnair e*	*Nationally representative*
GDD 2017	Demographic and Health Survey Colombia 2005	2004	2005	Colombia	*Both urban and rural*	*9309*	*0*	*4*	*Yes*	*Yes*	*DHS questionnair e*	*Nationally representative*
GDD 2017	Demographic and Health Survey Colombia 2010	2009	2009	Colombia	*Both urban and rural*	*11298*	*0*	*4*	*Yes*	*Yes*	*DHS questionnair e*	*Nationally representative*
GDD 2017	Demographic and Health Survey Colombia 1990	1990	1990	Colombia	*Both urban and rural*	*1175*	*0*	*4*	*Yes*	*Yes*	*DHS questionnair e*	*Nationally representative*

GDD 2010	Database from National survey of nutritional situation in Colombia	2005	2005	Colombia	*Information not reported*	*1294*	*20*	*64*	*Yes*	*Yes*	*Multiple 24-hour recall*	*Nationally representative*
GDD 2010	INTERSALT, Tuquerres	1985	1987	Colombia	*Information not reported*	*24*	*20*	*59*	*Yes*	*Yes*	*Biomarker*	*Sub-nationally representative*
GDD 2017	World Health Survey 2003	2003	2003	Comoros	*Both urban and rural*	*1632*	*18*	*90*	*Yes*	*Yes*	*FFQ*	*Nationally representative*
GDD 2017	Demographic and Health Survey Comoros 2012	2012	2012	Comoros	*Both urban and rural*	*2061*	*0*	*4*	*Yes*	*Yes*	*DHS questionnair e*	*Nationally representative*
GDD 2017	Demographic and Health Survey Comoros 1996	1996	1996	Comoros	*Both urban and rural*	*1039*	*0*	*2*	*Yes*	*Yes*	*DHS questionnair e*	*Nationally representative*
GDD 2017	World Health Survey 2003	2003	2003	Congo	*Both urban and rural*	*1698*	*16*	*85*	*Yes*	*Yes*	*FFQ*	*Nationally representative*
GDD 2017	Demographic and Health Survey Congo 2011	2011	2012	Congo	*Both urban and rural*	*12848*	*0*	*49*	*Yes*	*Yes*	*DHS questionnair e*	*Nationally representative*
GDD 2017	Demographic and Health Survey Congo 2005	2005	2005	Congo	*Both urban and rural*	*3307*	*0*	*4*	*Yes*	*Yes*	*DHS questionnair e*	*Nationally representative*
GDD 2010	STEP Congo	2004	2004	Congo	*Information not reported*	*1000*	*25*	*64*	*Yes*	*Yes*	*FFQ*	*Sub-nationally representative*
GDD 2017	Contribution of wild edible plants to women's diets	2009	2009	Congo, Democratic Republic	*Both urban and rural*	*960*	*15*	*72*	*No*	*Yes*	*Multiple 24-hour recall*	*Locally representative*
GDD 2017	Demographic and Health Survey Democratic Republic of Congo 2007	2007	2007	Congo, Democratic Republic	*Both urban and rural*	*11112*	*0*	*49*	*Yes*	*Yes*	*DHS questionnair e*	*Nationally representative*
GDD 2017	Demographic and Health Survey Democratic Republic of Congo 2013	2013	2014	Congo, Democratic Republic	*Both urban and rural*	*32550*	*0*	*59*	*Yes*	*Yes*	*DHS questionnair e*	*Nationally representative*
GDD 2010	Enqu�te sur les facteurs de risque des maladies non transmissibles � Kinshasa, capitale de la RD Congo, Selon l'approche STEPS de l'OMS	2005	2005	Congo, Democratic Republic	*Information not reported*	*1000*	*15*	*100*	*Yes*	*Yes*	*FFQ*	*Sub-nationally representative*
GDD 2017	Global Student Based Student Health Survey 2011	2011	2011	Cook Islands	*Both urban and rural*	*1269*	*11*	*16*	*Yes*	*Yes*	*FFQ*	*Nationally representative*
GDD 2010	STEP Cook Islands	2003	2003	Cook Islands	*Information not reported*	*1000*	*25*	*64*	*Yes*	*Yes*	*FFQ*	*Nationally representative*

GDD 2017	Global Student Based Student Health Survey 2009	2009	2009	Costa Rica	*Both urban and rural*	*2657*	*11*	*16*	*Yes*	*Yes*	*FFQ*	*Nationally representative*
GDD 2017	World Health Survey 2003	2003	2003	Cote D'Ivoire	*Both urban and rural*	*2515*	*18*	*95*	*Yes*	*Yes*	*FFQ*	*Nationally representative*
GDD 2017	Demographic and Health Survey Cote d'Ivoire 1994	1994	1994	Cote D'Ivoire	*Both urban and rural*	*2529*	*0*	*2*	*Yes*	*Yes*	*DHS questionnair e*	*Nationally representative*
GDD 2017	Demographic and Health Survey Cote d'Ivoire 2011	2011	2012	Cote D'Ivoire	*Both urban and rural*	*14808*	*0*	*59*	*Yes*	*Yes*	*DHS questionnair e*	*Nationally representative*
GDD 2017	Demographic and Health Surve Cote d'Ivoire 1998	1998	1999	Cote D'Ivoire	*Both urban and rural*	*1243*	*0*	*4*	*Yes*	*Yes*	*DHS questionnair e*	*Nationally representative*
GDD 2010	Enqu�te sur les facteurs de risque des maladies non transmissibles, C�te d'Ivoire, R�gions sanitaires des Lagunes - 2005	2005	2005	Cote D'Ivoire	*Information not reported*	*1000*	*15*	*100*	*Yes*	*Yes*	*FFQ*	*Sub-nationally representative*
GDD 2010	Hess et al. (1 of 2)	1997	1997	Cote D'Ivoire	*Information not reported*	*52*	*20*	*79*	*Yes*	*Yes*	*Biomarker*	*Sub-nationally representative*
GDD 2010	Hess et al. (2 of 2)	1997	1997	Cote D'Ivoire	*Information not reported*	*51*	*20*	*79*	*Yes*	*Yes*	*Biomarker*	*Sub-nationally representative*
GDD 2017	World Health Survey 2003	2003	2003	Croatia	*Both urban and rural*	*944*	*18*	*93*	*Yes*	*Yes*	*FFQ*	*Nationally representative*
GDD 2017	Health Behavior in School-Aged Children 2002	2001	2002	Croatia	*Both urban and rural*	*4366*	*11*	*16*	*Yes*	*Yes*	*FFQ*	*Nationally representative*
GDD 2017	Health Behavior in School-Aged Children 2006	2006	2006	Croatia	*Both urban and rural*	*4948*	*11*	*15*	*Yes*	*Yes*	*FFQ*	*Nationally representative*
GDD 2017	Health Behavior in School-Aged Children 2010	2010	2010	Croatia	*Both urban and rural*	*6225*	*11*	*15*	*Yes*	*Yes*	*FFQ*	*Nationally representative*
GDD 2010	Croatian Community Health and Nutrition Survey	1999	1999	Croatia	*Information not reported*	*1000*	*0*	*100*	*Yes*	*Yes*	*Household availability/ budget survey*	*Nationally representative*
GDD 2010	Croatian Community Health and Nutrition Survey	2004	2004	Croatia	*Information not reported*	*1000*	*0*	*100*	*Yes*	*Yes*	*Household availability/ budget survey*	*Nationally representative*

GDD 2010	Cypriot Community Health and Nutrition Survey	1996	1997	Cyprus	*Information not reported*	*1000*	*0*	*100*	*Yes*	*Yes*	*Household availability/ budget survey*	*Nationally representative*
GDD 2010	Cypriot Community Health and Nutrition Survey	2003	2003	Cyprus	*Information not reported*	*1000*	*0*	*100*	*Yes*	*Yes*	*Household availability/ budget survey*	*Nationally representative*
GDD 2017	World Health Survey 2003	2003	2003	Czech Republic	*Both urban and rural*	*875*	*18*	*93*	*Yes*	*Yes*	*FFQ*	*Nationally representative*
GDD 2017	Health Behavior in School-Aged Children 1994	1993	1994	Czech Republic	*Both urban and rural*	*3585*	*11*	*17*	*Yes*	*Yes*	*FFQ*	*Nationally representative*
GDD 2017	Health Behavior in School-Aged Children 1998	1998	1998	Czech Republic	*Both urban and rural*	*3647*	*11*	*16*	*Yes*	*Yes*	*FFQ*	*Nationally representative*
GDD 2017	Health Behavior in School-Aged Children 2002	2001	2002	Czech Republic	*Both urban and rural*	*5012*	*11*	*16*	*Yes*	*Yes*	*FFQ*	*Nationally representative*
GDD 2017	Health Behavior in School-Aged Children 2006	2006	2006	Czech Republic	*Both urban and rural*	*4750*	*11*	*15*	*Yes*	*Yes*	*FFQ*	*Nationally representative*
GDD 2017	Health Behavior in School-Aged Children 2010	2010	2010	Czech Republic	*Both urban and rural*	*4391*	*11*	*15*	*Yes*	*Yes*	*FFQ*	*Nationally representative*
GDD 2010	Individual food consumption study	2003	2004	Czech Republic	*Information not reported*	*1000*	*16*	*64*	*Yes*	*Yes*	*Multiple 24-hour recall*	*Nationally representative*
GDD 2010	Nutritional status of Czech children			Czech Republic	*Information not reported*	*1000*	*4*	*9*	*Yes*	*Yes*	*Multiple 24-hour recall*	*Nationally representative*
GDD 2010	Czech Post-MONICA study			Czech Republic	*Information not reported*	*1000*	*19*	*64*	*Yes*	*Yes*	*Single 24-hour recall*	*Nationally representative*
GDD 2017	Health Behavior in School-Aged Children 1994	1993	1994	Denmark	*Both urban and rural*	*3792*	*11*	*17*	*Yes*	*Yes*	*FFQ*	*Nationally representative*
GDD 2017	Health Behavior in School-Aged Children 1998	1998	1998	Denmark	*Both urban and rural*	*4934*	*11*	*17*	*Yes*	*Yes*	*FFQ*	*Nationally representative*
GDD 2017	Health Behavior in School-Aged Children 2002	2001	2002	Denmark	*Both urban and rural*	*4587*	*11*	*17*	*Yes*	*Yes*	*FFQ*	*Nationally representative*
GDD 2017	Health Behavior in School-Aged Children 2006	2006	2006	Denmark	*Both urban and rural*	*5667*	*11*	*15*	*Yes*	*Yes*	*FFQ*	*Nationally representative*

GDD 2017	Health Behavior in School-Aged Children 2010	2010	2010	Denmark	*Both urban and rural*	*4019*	*11*	*15*	*Yes*	*Yes*	*FFQ*	*Nationally representative*
GDD 2010	Dietary Habits of Denmark	2000	2002	Denmark	*Information not reported*	*500*	*4*	*14*	*Yes*	*Yes*	*Multiple 24-hour recall*	*Nationally representative*
GDD 2010	Dietary Habits of Denmark	2000	2002	Denmark	*Information not reported*	*1283*	*15*	*100*	*Yes*	*Yes*	*Multiple 24-hour recall*	*Nationally representative*
GDD 2010	Report of Dietary habits in Denmark from 2000-2002	2000	2002	Denmark	*Information not reported*	*1467*	*20*	*75*	*Yes*	*Yes*	*Multiple 24-hour recall*	*Nationally representative*
GDD 2010	TRANSFAIR study	1995	1995	Denmark	*Information not reported*	*500*	*19*	*64*	*Yes*	*Yes*	*Single 24-hour recall*	*Nationally representative*
GDD 2010	INTERSALT, Glostrup	1985	1987	Denmark	*Information not reported*	*24*	*20*	*59*	*Yes*	*Yes*	*Biomarker*	*Sub-nationally representative*
GDD 2010	Andersen et al.	2006	2006	Denmark	*Information not reported*	*37*	*20*	*55*	*Yes*	*Yes*	*Biomarker*	*Sub-nationally representative*
GDD 2017	Global Student Based Student Health Survey 2009	2009	2009	Dominica	*Both urban and rural*	*1628*	*11*	*16*	*Yes*	*Yes*	*FFQ*	*Nationally representative*
GDD 2010	WHO STEPS Survey for Dominica 2007	2007	2007	Dominica	*Information not reported*	*1000*	*15*	*64*	*Yes*	*Yes*	*FFQ*	*Nationally representative*
GDD 2017	World Health Survey 2003	2003	2003	Dominican Republic	*Both urban and rural*	*3503*	*18*	*103*	*Yes*	*Yes*	*FFQ*	*Nationally representative*
GDD 2017	Demographic and Health Survey Dominican Republic 1986	1986	1986	Dominican Republic	*Both urban and rural*	*816*	*0*	*4*	*Yes*	*Yes*	*DHS questionnair e*	*Nationally representative*
GDD 2017	Demographic and Health Survey Dominican Republic 1991	1991	1991	Dominican Republic	*Both urban and rural*	*678*	*0*	*4*	*Yes*	*Yes*	*DHS questionnair e*	*Nationally representative*
GDD 2017	Demographic and Health Survey Dominican Republic 1996	1996	1996	Dominican Republic	*Both urban and rural*	*2955*	*0*	*4*	*Yes*	*Yes*	*DHS questionnair e*	*Nationally representative*
GDD 2017	Demographic and Health Survey Dominican Repoublic 1999	1999	1999	Dominican Republic	*Both urban and rural*	*564*	*0*	*4*	*Yes*	*Yes*	*DHS questionnair e*	*Nationally representative*
GDD 2017	Demographic and Health Survey Dominican Republic 2007	2007	2007	Dominican Republic	*Both urban and rural*	*12892*	*0*	*48*	*Yes*	*Yes*	*DHS questionnair e*	*Nationally representative*
GDD 2017	Demographic and Health Survey Dominican Republic 2013	2013	2013	Dominican Republic	*Both urban and rural*	*1874*	*0*	*4*	*Yes*	*Yes*	*DHS questionnair e*	*Nationally representative*
GDD 2017	DHS IV	2002	2002	Dominican Republic	*Both urban and rural*	*10074*	*0*	*4*	*Yes*	*Yes*	*DHS questionnair e*	*Nationally representative*

GDD 2017	Baseline of child nutritional status	2008	2008	Ecuador	*Only urban*	*650*	*0*	*2*	*Yes*	*Yes*	*Multiple 24-hour recall*	*Locally representative*
GDD 2017	Baseline survey of Nutritional Status in a rural highland area	2008	2008	Ecuador	*Only rural*	*394*	*0*	*2*	*Yes*	*Yes*	*Single 24-hour recall*	*Locally representative*
GDD 2017	World Health Survey 2003	2003	2003	Ecuador	*Both urban and rural*	*2607*	*18*	*95*	*Yes*	*Yes*	*FFQ*	*Nationally representative*
GDD 2017	Global Student Based Student Health Survey 2007	2007	2007	Ecuador	*Only urban*	*5386*	*11*	*16*	*Yes*	*Yes*	*FFQ*	*Nationally representative*
GDD 2017	Demographic and Health Survey Ecuador 1987	1987	1987	Ecuador	*Both urban and rural*	*755*	*0*	*3*	*Yes*	*Yes*	*DHS questionnaire*	*Nationally representative*
GDD 2017	Global Student Based Student Health Survey 2006	2006	2006	Egypt	*Both urban and rural*	*5084*	*11*	*16*	*Yes*	*Yes*	*FFQ*	*Nationally representative*
GDD 2017	Global Student Based Student Health Survey 2011	2011	2011	Egypt	*Both urban and rural*	*2478*	*11*	*16*	*Yes*	*Yes*	*FFQ*	*Nationally representative*
GDD 2017	Demographic and Health Survey Egypt 1995	1995	1995	Egypt	*Both urban and rural*	*7662*	*0*	*4*	*Yes*	*Yes*	*DHS questionnaire*	*Nationally representative*
GDD 2017	Demographic and Health Survey Egypt 2000	2000	2000	Egypt	*Both urban and rural*	*18506*	*0*	*49*	*Yes*	*Yes*	*DHS questionnaire*	*Nationally representative*
GDD 2017	Demographic and Health Survey Egypt 2003	2003	2003	Egypt	*Both urban and rural*	*5047*	*0*	*4*	*Yes*	*Yes*	*DHS questionnaire*	*Nationally representative*
GDD 2017	Demographic and Health Survey Egypt 2005	2005	2005	Egypt	*Both urban and rural*	*23678*	*0*	*49*	*Yes*	*Yes*	*DHS questionnaire*	*Nationally representative*
GDD 2017	Demographic and Health Survey Egypt 2008	2008	2008	Egypt	*Both urban and rural*	*14443*	*0*	*49*	*Yes*	*Yes*	*DHS questionnaire*	*Nationally representative*
GDD 2017	Demographic and Health Survey Egypt 2014	2014	2014	Egypt	*Both urban and rural*	*20643*	*0*	*49*	*Yes*	*Yes*	*DHS questionnaire*	*Nationally representative*
GDD 2010	Multi-centre study of the Mediterranean Group for the Study of Diabetes (MGSD)	1995		Egypt	*Information not reported*	*100*	*35*	*60*	*Yes*	*Yes*	*FFQ*	*Sub-nationally representative*
GDD 2017	Global Student Based Student Health Survey 2013	2013	2013	El Salvador	*Both urban and rural*	*1862*	*11*	*16*	*Yes*	*Yes*	*FFQ*	*Nationally representative*

GDD 2010	National noncommunicable disease (NCD) risk factor baseline survey (using WHO STEPSwise approach)	2004	2004	Eritrea	*Information not reported*	*1000*	*15*	*64*	*Yes*	*Yes*	*FFQ*	*Nationally representative*
GDD 2017	World Health Survey 2003	2003	2003	Estonia	*Both urban and rural*	*933*	*18*	*92*	*Yes*	*Yes*	*FFQ*	*Nationally representative*
GDD 2017	Health Behavior in School-Aged Children 1994	1993	1994	Estonia	*Both urban and rural*	*3516*	*10*	*16*	*Yes*	*Yes*	*FFQ*	*Nationally representative*
GDD 2017	Health Behavior in School-Aged Children 1998	1998	1998	Estonia	*Both urban and rural*	*1897*	*11*	*16*	*Yes*	*Yes*	*FFQ*	*Nationally representative*
GDD 2017	Health Behavior in School-Aged Children 2002	2001	2002	Estonia	*Both urban and rural*	*3976*	*11*	*16*	*Yes*	*Yes*	*FFQ*	*Nationally representative*
GDD 2017	Health Behavior in School-Aged Children 2006	2006	2006	Estonia	*Both urban and rural*	*4458*	*11*	*15*	*Yes*	*Yes*	*FFQ*	*Nationally representative*
GDD 2017	Health Behavior in School-Aged Children 2010	2010	2010	Estonia	*Both urban and rural*	*4202*	*11*	*15*	*Yes*	*Yes*	*FFQ*	*Nationally representative*
GDD 2010	Estonian National Dietary Survey	1997	1997	Estonia	*Information not reported*	*1000*	*16*	*64*	*Yes*	*Yes*	*Single 24-hour recall*	*Nationally representative*
GDD 2017	Nutritional status and dietary intake of urban residents in Gondar, Northwest Ethiopia	2004	2007	Ethiopia	*Only urban*	*356*	*18*	*80*	*Yes*	*Yes*	*FFQ*	*Sub-nationally representative*
GDD 2017	Demographic and Health Study 2000	2000	2000	Ethiopia	*Both urban and rural*	*9203*	*0*	*4*	*Yes*	*Yes*	*DHS questionnaire*	*Nationally representative*
GDD 2017	Demographic and Health Study 2005	2005	2005	Ethiopia	*Both urban and rural*	*17526*	*0*	*49*	*Yes*	*Yes*	*DHS questionnaire*	*Nationally representative*
GDD 2017	Demographic and Health Survey 2011	2011	2011	Ethiopia	*Both urban and rural*	*6692*	*0*	*4*	*Yes*	*Yes*	*DHS questionnaire*	*Nationally representative*
GDD 2017	Ethiopia World Health Study 2003	2003	2003	Ethiopia	*Both urban and rural*	*2803*	*16*	*106*	*Yes*	*Yes*	*FFQ*	*Nationally representative*
GDD 2017	Global Student Based Student Health Survey 2010	2010	2010	Fiji	*Both urban and rural*	*1658*	*12*	*16*	*Yes*	*Yes*	*FFQ*	*Nationally representative*

GDD 2017	Health Behavior in School-Aged Children 1986	1985	1986	Finland	*Both urban and rural*	*3179*	*11*	*17*	*Yes*	*Yes*	*FFQ*	*Nationally representative*
GDD 2017	Health Behavior in School-Aged Children 1990	1989	1990	Finland	*Both urban and rural*	*2960*	*11*	*17*	*Yes*	*Yes*	*FFQ*	*Nationally representative*
GDD 2017	Health Behavior in School-Aged Children 1994	1993	1994	Finland	*Both urban and rural*	*4074*	*11*	*17*	*Yes*	*Yes*	*FFQ*	*Nationally representative*
GDD 2017	Health Behavior in School-Aged Children 1998	1998	1998	Finland	*Both urban and rural*	*4827*	*11*	*17*	*Yes*	*Yes*	*FFQ*	*Nationally representative*
GDD 2017	Health Behavior in School-Aged Children 2002	2001	2002	Finland	*Both urban and rural*	*5348*	*11*	*17*	*Yes*	*Yes*	*FFQ*	*Nationally representative*
GDD 2017	Health Behavior in School-Aged Children 2006	2006	2006	Finland	*Both urban and rural*	*5187*	*11*	*15*	*Yes*	*Yes*	*FFQ*	*Nationally representative*
GDD 2017	Health Behavior in School-Aged Children 2010	2010	2010	Finland	*Both urban and rural*	*6606*	*11*	*15*	*Yes*	*Yes*	*FFQ*	*Nationally representative*
GDD 2010	Finn Community Health and Nutrition Survey	1985	1985	Finland	*Information not reported*	*1000*	*0*	*100*	*Yes*	*Yes*	*Household availability/ budget survey*	*Nationally representative*
GDD 2010	Finn Community Health and Nutrition Survey	1990	1990	Finland	*Information not reported*	*1000*	*0*	*100*	*Yes*	*Yes*	*Household availability/ budget survey*	*Nationally representative*
GDD 2010	FINDIET 1992	1992	1992	Finland	*Information not reported*	*1000*	*25*	*64*	*Yes*	*Yes*	*Multiple 24-hour recall*	*Nationally representative*
GDD 2010	Finn Community Health and Nutrition Survey	1998	1998	Finland	*Information not reported*	*1000*	*0*	*100*	*Yes*	*Yes*	*Household availability/ budget survey*	*Nationally representative*
GDD 2010	FINDIET 2002	2002	2002	Finland	*Information not reported*	*1000*	*25*	*64*	*Yes*	*Yes*	*Multiple 24-hour recall*	*Nationally representative*
GDD 2010	Type 1 Diabetes Prediction and Prevention (DIPP) Nutrition Study	2003	2005	Finland	*Information not reported*	*1000*	*4*	*6*	*Yes*	*Yes*	*Multiple 24-hour recall*	*Nationally representative*
GDD 2010	FINDIET 2007	2007	2007	Finland	*Information not reported*	*1000*	*25*	*74*	*Yes*	*Yes*	*Multiple 24-hour recall*	*Nationally representative*
GDD 2010	TRANSFAIR study	1992	1992	Finland	*Information not reported*	*500*	*25*	*64*	*Yes*	*Yes*	*Single 24-hour recall*	*Nationally representative*

GDD 2010	INTERSALT, Turku	1985	1987	Finland	*Information not reported*	*25*	*20*	*59*	*Yes*	*Yes*	*Biomarker*	*Sub-nationally representative*
GDD 2010	Laatikainen et al. (2 of 11)	1982	1982	Finland	*Information not reported*	*247*	*25*	*64*	*Yes*	*Yes*	*Biomarker*	*Sub-nationally representative*
GDD 2010	Laatikainen et al. (3 of 11)	1987	1987	Finland	*Information not reported*	*199*	*25*	*64*	*Yes*	*Yes*	*Biomarker*	*Sub-nationally representative*
GDD 2010	Laatikainen et al. (4 of 11)	2002	2002	Finland	*Information not reported*	*168*	*25*	*64*	*Yes*	*Yes*	*Biomarker*	*Sub-nationally representative*
GDD 2010	Laatikainen et al. (6 of 11)	1982	1982	Finland	*Information not reported*	*213*	*25*	*64*	*Yes*	*Yes*	*Biomarker*	*Sub-nationally representative*
GDD 2010	Laatikainen et al. (7 of 11)	1987	1987	Finland	*Information not reported*	*180*	*25*	*64*	*Yes*	*Yes*	*Biomarker*	*Sub-nationally representative*
GDD 2010	Laatikainen et al. (8 of 11)	1982	1982	Finland	*Information not reported*	*232*	*25*	*64*	*Yes*	*Yes*	*Biomarker*	*Sub-nationally representative*
GDD 2010	Laatikainen et al. (9 of 11)	1987	1987	Finland	*Information not reported*	*150*	*25*	*64*	*Yes*	*Yes*	*Biomarker*	*Sub-nationally representative*
GDD 2010	Laatikainen et al. (11 of 11)	2002	2002	Finland	*Information not reported*	*127*	*25*	*64*	*Yes*	*Yes*	*Biomarker*	*Sub-nationally representative*
GDD 2010	Reinivuo et al.	2002	2002	Finland	*Information not reported*	*168*	*25*	*64*	*Yes*	*Yes*	*Multiple 24-hour recall*	*Sub-nationally representative*
GDD 2010	Reinivuo et al.	2002	2002	Finland	*Information not reported*	*128*	*25*	*64*	*Yes*	*Yes*	*Multiple 24-hour recall*	*Sub-nationally representative*
GDD 2010	Reinivuo et al.	2002	2002	Finland	*Information not reported*	*127*	*25*	*64*	*Yes*	*Yes*	*Multiple 24-hour recall*	*Sub-nationally representative*
GDD 2010	INTERSALT, Joensuu	1985	1987	Finland	*Information not reported*	*25*	*20*	*59*	*Yes*	*Yes*	*Biomarker*	*Sub-nationally representative*
GDD 2010	Laatikainen et al. (10 of 11)	2002	2002	Finland	*Information not reported*	*128*	*25*	*64*	*Yes*	*Yes*	*Biomarker*	*Sub-nationally representative*
GDD 2017	Etude nationale nutrition santé (ENNS); National Nutrition and Health survey	2006	2007	France	*Both urban and rural*	*4429*	*3*	*75*	*Yes*	*Yes*	*Multiple 24-hour recall*	*Nationally representative*
GDD 2017	Health Behavior in School-Aged Children 1994	1993	1994	France	*Both urban and rural*	*4004*	*11*	*16*	*Yes*	*Yes*	*FFQ*	*Nationally representative*
GDD 2017	Health Behavior in School-Aged Children 1998	1998	1998	France	*Both urban and rural*	*4133*	*11*	*16*	*Yes*	*Yes*	*FFQ*	*Sub-nationally representative*
GDD 2017	Health Behavior in School-Aged Children 2002	2001	2002	France	*Both urban and rural*	*8185*	*10*	*16*	*Yes*	*Yes*	*FFQ*	*Nationally representative*
GDD 2017	Health Behavior in School-Aged Children 2006	2006	2006	France	*Both urban and rural*	*7094*	*11*	*15*	*Yes*	*Yes*	*FFQ*	*Nationally representative*

GDD 2017	Health Behavior in School-Aged Children 2010	2010	2010	France	*Both urban and rural*	*6073*	*11*	*15*	*Yes*	*Yes*	*FFQ*	*Nationally representative*
GDD 2010	Enquete Individuelle et Nationale sur les Consommations Alimentaires	1999	1999	France	*Information not reported*	*1000*	*16*	*64*	*Yes*	*Yes*	*Multiple 24-hour recall*	*Nationally representative*
GDD 2010	Etude nationale nutrition sant� (ENNS); National Nutrition and Health survey	2006	2007	France	*Information not reported*	*1000*	*4*	*74*	*Yes*	*Yes*	*Multiple 24-hour recall*	*Nationally representative*
GDD 2010	TRANSFAIR study	1993	1994	France	*Information not reported*	*250*	*19*	*64*	*Yes*	*Yes*	*Single 24-hour recall*	*Nationally representative*
GDD 2010	du Cailar et al.	2001	2001	France	*Information not reported*	*438*	*20*	*70*	*Yes*	*Yes*	*Biomarker*	*Locally representative*
GDD 2010	Meneton et al. (1 of 2)	1998	1999	France	*Information not reported*	*672*	*20*	*92*	*Yes*	*Yes*	*Multiple 24-hour recall*	*Nationally representative*
GDD 2017	Demographic and Health Survey Gabon 2012	2012	2012	Gabon	*Both urban and rural*	*15968*	*0*	*59*	*Yes*	*Yes*	*DHS questionnaire*	*Nationally representative*
GDD 2017	Demographic and Health Survey Gabon 2000	2000	2001	Gabon	*Both urban and rural*	*2603*	*0*	*4*	*Yes*	*Yes*	*DHS questionnaire*	*Nationally representative*
GDD 2010	STEP Gabon	2009	2009	Gabon	*Information not reported*	*1000*	*15*	*64*	*Yes*	*Yes*	*FFQ*	*Sub-nationally representative*
GDD 2017	DHS VI	2013	2013	Gambia	*Both urban and rural*	*15579*	*0*	*96*	*Yes*	*Yes*	*DHS questionnaire*	*Nationally representative*
GDD 2017	World Health Survey 2003	2003	2003	Georgia	*Both urban and rural*	*2399*	*18*	*102*	*Yes*	*Yes*	*FFQ*	*Nationally representative*
GDD 2010	Living Conditions Lifestyles and Health	2001	2002	Georgia	*Information not reported*	*1000*	*18*	*99*	*Yes*	*Yes*	*FFQ*	*Nationally representative*
GDD 2017	German Nutrition Survey 1998	1997	1999	Germany	*Both urban and rural*	*4030*	*15*	*85*	*Yes*	*Yes*	*FFQ*	*Nationally representative*
GDD 2017	EsKiMo	2006	2006	Germany	*Both urban and rural*	*1272*	*12*	*17*	*Yes*	*Yes*	*FFQ*	*Nationally representative*
GDD 2017	Health Behavior in School-Aged Children 1994	1993	1994	Germany	*Both urban and rural*	*3275*	*11*	*16*	*Yes*	*Yes*	*FFQ*	*Nationally representative*
GDD 2017	Health Behavior in School-Aged Children 1998	1998	1998	Germany	*Both urban and rural*	*4781*	*10*	*16*	*Yes*	*Yes*	*FFQ*	*Sub-nationally representative*
GDD 2017	Health Behavior in School-Aged Children 2002	2001	2002	Germany	*Both urban and rural*	*5635*	*11*	*17*	*Yes*	*Yes*	*FFQ*	*Nationally representative*

GDD 2017	Health Behavior in School-Aged Children 2006	2006	2006	Germany	*Both urban and rural*	*7169*	*11*	*15*	*Yes*	*Yes*	*FFQ*	*Nationally representative*	
GDD 2017	Health Behavior in School-Aged Children 2010	2010	2010	Germany	*Both urban and rural*	*4942*	*11*	*15*	*Yes*	*Yes*	*FFQ*	*Nationally representative*	
GDD 2010	German Community Health and Nutrition Survey	1988	1988	Germany	*Information not reported*	*1000*	*0*	*100*	*Yes*	*Yes*	*Household availability/ budget survey*	*Nationally representative*	
GDD 2010	German Community Health and Nutrition Survey	1993	1993	Germany	*Information not reported*	*1000*	*0*	*100*	*Yes*	*Yes*	*Household availability/ budget survey*	*Nationally representative*	
GDD 2010	German Nutrition Survey 1998	1997	1999	Germany	*Information not reported*	*1000*	*20*	*79*	*Yes*	*Yes*	*FFQ*	*Nationally representative*	
GDD 2010	German Nutrition Survey 1998	1997	1999	Germany	*Information not reported*	*1000*	*18*	*64*	*Yes*	*Yes*	*FFQ*	*Nationally representative*	
GDD 2010	German Community Health and Nutrition Survey	1998	1998	Germany	*Information not reported*	*1000*	*0*	*100*	*Yes*	*Yes*	*Household availability/ budget survey*	*Nationally representative*	
GDD 2010	Nationale Verzehrsstudie II	2005	2007	Germany	*Information not reported*	*1000*	*15*	*100*	*Yes*	*Yes*	*Multiple 24-hour recall*	*Nationally representative*	
GDD 2010	Nationale Verzehrs Studie II, Ergebnisbericht, Teil 2	2005	2006	Germany	*Information not reported*	*510*	*20*	*80*	*Yes*	*Yes*	*Multiple 24-hour recall*	*Nationally representative*	
GDD 2010	EsKiMo � Ern�hrungsstudie als KIGGS-Modul	2006	2006	Germany	*Information not reported*	*1000*	*6*	*14*	*Yes*	*Yes*	*Multiple 24-hour recall*	*Nationally representative*	
GDD 2010	TRANSFAIR study	1991	1991	Germany	*Information not reported*	*500*	*19*	*64*	*Yes*	*Yes*	*Single 24-hour recall*	*Nationally representative*	
GDD 2010	INTERSALT, Bernried	1985	1987	Germany	*Information not reported*	*25*	*20*	*59*	*Yes*	*Yes*	*Biomarker*	*Sub-nationally representative*	
GDD 2010	INTERSALT, Cottbus	1985	1987	Germany	*Information not reported*	*25*	*20*	*59*	*Yes*	*Yes*	*Biomarker*	*Sub-nationally representative*	
GDD 2010	INTERSALT, Heidelberg	1985	1987	Germany	*Information not reported*	*22*	*20*	*59*	*Yes*	*Yes*	*Biomarker*	*Sub-nationally representative*	
GDD 2017	Modeling the Epidemiologic Transition Study (METS) - Ghana	2010	2012	Ghana	*Only rural*	*944*	*23*	*45*	*Yes*	*Yes*	*Multiple 24-hour recall*	*Locally representative*	
GDD 2017	Demographic and Health Survey 2003	2003	2003	Ghana	*Both urban and rural*	*3403*	*0*	*4*	*Yes*	*Yes*	*DHS questionnair e*	*Nationally representative*	

GDD 2017	Demographic and Health Survey 2008	2008	2008	Ghana	*Both urban and rural*	*7025*	*0*	*49*	*Yes*	*Yes*	*DHS questionnair e*	*Nationally representative*
GDD 2017	Demographic and Health Survey 2014	2014	2014	Ghana	*Both urban and rural*	*9153*	*0*	*49*	*Yes*	*Yes*	*DHS questionnair e*	*Nationally representative*
GDD 2017	Global School-Based Student Health Survey 2007	2007	2007	Ghana	*Both urban and rural*	*5989*	*11*	*16*	*Yes*	*Yes*	*FFQ*	*Nationally representative*
GDD 2017	Global School-Based Student Health Survey Junior High 2012	2012	2012	Ghana	*Both urban and rural*	*1616*	*11*	*18*	*Yes*	*Yes*	*FFQ*	*Nationally representative*
GDD 2017	Global School-Based Student Health Survey Senior High 2012	2012	2012	Ghana	*Both urban and rural*	*1962*	*12*	*18*	*Yes*	*Yes*	*FFQ*	*Nationally representative*
GDD 2017	World Health Survey 2003	2003	2003	Ghana	*Both urban and rural*	*3220*	*18*	*107*	*Yes*	*Yes*	*FFQ*	*Nationally representative*
GDD 2017	Study on Global Ageing and Adult Health	2007	2008	Ghana	*Both urban and rural*	*5051*	*18*	*120*	*Yes*	*Yes*	*FFQ*	*Nationally representative*
GDD 2010	Kerry et al. (1 of 2)	2001	2002	Ghana	*Information not reported*	*481*	*40*	*75*	*Yes*	*Yes*	*Biomarker*	*Sub-nationally representative*
GDD 2010	Kerry et al. (2 of 2)	2001	2002	Ghana	*Information not reported*	*532*	*40*	*75*	*Yes*	*Yes*	*Biomarker*	*Locally representative*
GDD 2017	Health Behavior in School-Aged Children 1998	1998	1998	Greece	*Both urban and rural*	*4211*	*11*	*17*	*Yes*	*Yes*	*FFQ*	*Nationally representative*
GDD 2017	Health Behavior in School-Aged Children 2002	2001	2002	Greece	*Both urban and rural*	*3807*	*11*	*16*	*Yes*	*Yes*	*FFQ*	*Nationally representative*
GDD 2017	Health Behavior in School-Aged Children 2006	2006	2006	Greece	*Both urban and rural*	*3673*	*11*	*15*	*Yes*	*Yes*	*FFQ*	*Nationally representative*
GDD 2017	Health Behavior in School-Aged Children 2010	2010	2010	Greece	*Both urban and rural*	*4886*	*11*	*15*	*Yes*	*Yes*	*FFQ*	*Nationally representative*
GDD 2010	Greek Community Health Survey and Nutrition Nutrition	1981	1981	Greece	*Information not reported*	*1000*	*0*	*100*	*Yes*	*Yes*	*Household availability/ budget survey*	*Nationally representative*
GDD 2010	Greek Community Health Survey and Nutrition Nutrition	1987	1987	Greece	*Information not reported*	*1000*	*0*	*100*	*Yes*	*Yes*	*Household availability/ budget survey*	*Nationally representative*

GDD 2010	Greek component of the European Prospective Investigation into Cancer and Nutrition (EPIC)	1994	1999	Greece	*Information not reported*	*1000*	*25*	*84*	*Yes*	*Yes*	*FFQ*	*Locally representative*
GDD 2010	Greek component of the European Prospective Investigation into Cancer and Nutrition (EPIC)	1994	1999	Greece	*Information not reported*	*1000*	*19*	*84*	*Yes*	*Yes*	*FFQ*	*Locally representative*
GDD 2010	Multi-centre study of the Mediterranean Group for the Study of Diabetes (MGSD)	1995		Greece	*Information not reported*	*500*	*25*	*74*	*Yes*	*Yes*	*FFQ*	*Sub-nationally representative*
GDD 2010	Greek Community Health Survey and Nutrition Nutrition	1998	1998	Greece	*Information not reported*	*1000*	*0*	*100*	*Yes*	*Yes*	*Household availability/ budget survey*	*Nationally representative*
GDD 2010	ATTICA Study	2001	2002	Greece	*Information not reported*	*1000*	*18*	*75*	*Yes*	*Yes*	*FFQ*	*Sub-nationally representative*
GDD 2010	The GENESIS study	2003	2004	Greece	*Information not reported*	*1000*	*1*	*5*	*Yes*	*Yes*	*Multiple 24-hour recall*	*Nationally representative*
GDD 2010	Greek Community Health Survey and Nutrition Nutrition	2004	2004	Greece	*Information not reported*	*1000*	*0*	*100*	*Yes*	*Yes*	*Household availability/ budget survey*	*Nationally representative*
GDD 2010	TRANSFAIR study	1995	1995	Greece	*Information not reported*	*100*	*23*	*64*	*Yes*	*Yes*	*Single 24-hour recall*	*Nationally representative*
GDD 2017	Health Behavior in School-Aged Children 1994	1993	1994	Greenland	*Both urban and rural*	*1306*	*11*	*16*	*Yes*	*Yes*	*FFQ*	*Nationally representative*
GDD 2017	Health Behavior in School-Aged Children 1998	1998	1998	Greenland	*Both urban and rural*	*1629*	*11*	*17*	*Yes*	*Yes*	*FFQ*	*Nationally representative*
GDD 2017	Health Behavior in School-Aged Children 2002	2001	2002	Greenland	*Both urban and rural*	*873*	*11*	*17*	*Yes*	*Yes*	*FFQ*	*Nationally representative*
GDD 2017	Health Behavior in School-Aged Children 2006	2006	2006	Greenland	*Both urban and rural*	*1321*	*11*	*15*	*Yes*	*Yes*	*FFQ*	*Nationally representative*
GDD 2017	Health Behavior in School-Aged Children 2010	2010	2010	Greenland	*Both urban and rural*	*1158*	*11*	*15*	*Yes*	*Yes*	*FFQ*	*Nationally representative*
GDD 2010	Inuit Health in Transition	2005	2009	Greenland	*Information not reported*	*1000*	*20*	*89*	*Yes*	*Yes*	*FFQ*	*Nationally representative*

GDD 2017	Global Student Based Student Health Survey 2008	2008	2008	Grenada	*Both urban and rural*	*1517*	*11*	*16*	*Yes*	*Yes*	*FFQ*	*Nationally representative*
GDD 2017	Mam Mamas	2012	2013	Guatemala	*Only rural*	*299*	*14*	*42*	*No*	*Yes*	*Multiple 24-hour recall*	*Locally representative*
GDD 2017	Xela Babies	2011	2011	Guatemala	*Only urban*	*330*	*0*	*2*	*Yes*	*Yes*	*Single 24-hour recall*	*Locally representative*
GDD 2017	Non-pregnant & non-lactating women vitamin A survey	2014	2014	Guatemala	*Both urban and rural*	*250*	*18*	*45*	*No*	*Yes*	*Single 24-hour recall*	*Locally representative*
GDD 2017	Global Student Based Student Health Survey 2009	2009	2009	Guatemala	*Both urban and rural*	*5447*	*11*	*16*	*Yes*	*Yes*	*FFQ*	*Nationally representative*
GDD 2017	World Health Survey 2003	2003	2003	Guatemala	*Both urban and rural*	*4093*	*18*	*99*	*Yes*	*Yes*	*FFQ*	*Nationally representative*
GDD 2017	Demographic and Health Survey Guatemala 2014	2014	2015	Guatemala	*Both urban and rural*	*37909*	*0*	*49*	*Yes*	*Yes*	*DHS questionnaire*	*Nationally representative*
GDD 2017	Demographic and Health Survey Guatemala 1995	1995	1995	Guatemala	*Both urban and rural*	*5789*	*0*	*4*	*Yes*	*Yes*	*DHS questionnaire*	*Nationally representative*
GDD 2017	Demographic and Health Survey Guatemala 1998	1998	1999	Guatemala	*Both urban and rural*	*2908*	*0*	*4*	*Yes*	*Yes*	*DHS questionnaire*	*Nationally representative*
GDD 2010	Melse-Boonstra et al. (4 of 4)	1996	1996	Guatemala	*Information not reported*	*7*	*25*	*55*	*No*	*Yes*	*Biomarker*	*Sub-nationally representative*
GDD 2017	Demographic and Health Survey Guinea 2005	2005	2005	Guinea	*Both urban and rural*	*11987*	*0*	*59*	*Yes*	*Yes*	*DHS questionnaire*	*Nationally representative*
GDD 2017	Demographic and Health Survey Guinea 2012	2012	2012	Guinea	*Both urban and rural*	*13623*	*0*	*59*	*Yes*	*Yes*	*DHS questionnaire*	*Nationally representative*
GDD 2017	Demographic and Health Survey Guinea 1999	1999	1999	Guinea	*Both urban and rural*	*3597*	*0*	*4*	*Yes*	*Yes*	*DHS questionnaire*	*Nationally representative*
GDD 2017	Global Student Based Student Health Survey 2004	2004	2004	Guyana	*Both urban and rural*	*1189*	*11*	*16*	*Yes*	*Yes*	*FFQ*	*Nationally representative*
GDD 2017	Global Student Based Student Health Survey 2010	2010	2010	Guyana	*Both urban and rural*	*2357*	*11*	*16*	*Yes*	*Yes*	*FFQ*	*Nationally representative*
GDD 2017	Demographic and Health Survey Guyana 2009	2009	2009	Guyana	*Both urban and rural*	*2839*	*0*	*47*	*Yes*	*Yes*	*DHS questionnaire*	*Nationally representative*

GDD 2017	Demographic and Health Survey Haiti 1995	1995	1995	Haiti	*Both urban and rural*	*998*	*0*	*2*	*Yes*	*Yes*	*DHS questionnaire*	*Nationally representative*
GDD 2017	Demographic and Health Survey Haiti 2000	2000	2000	Haiti	*Both urban and rural*	*10789*	*0*	*49*	*Yes*	*Yes*	*DHS questionnaire*	*Nationally representative*
GDD 2017	Demographic and Health Survey Haiti 2005	2005	2006	Haiti	*Both urban and rural*	*11647*	*0*	*49*	*Yes*	*Yes*	*DHS questionnaire*	*Nationally representative*
GDD 2017	Demographic and Health Survey Haiti 2012	2012	2012	Haiti	*Both urban and rural*	*14796*	*0*	*49*	*Yes*	*Yes*	*DHS questionnaire*	*Nationally representative*
GDD 2017	Global Student Based Student Health Survey 2012	2012	2012	Honduras	*Both urban and rural*	*1742*	*11*	*16*	*Yes*	*Yes*	*FFQ*	*Nationally representative*
GDD 2017	Demographic and Health Survey Honduras 2005	2005	2006	Honduras	*Both urban and rural*	*28876*	*0*	*49*	*Yes*	*Yes*	*DHS questionnaire*	*Nationally representative*
GDD 2017	Demographic and Health Survey Honduras 2011	2011	2012	Honduras	*Both urban and rural*	*31660*	*0*	*49*	*Yes*	*Yes*	*DHS questionnaire*	*Nationally representative*
GDD 2017	World Health Survey 2003	2003	2003	Hungary	*Both urban and rural*	*1398*	*18*	*95*	*Yes*	*Yes*	*FFQ*	*Nationally representative*
GDD 2017	Health Behavior in School-Aged Children 1986	1985	1986	Hungary	*Both urban and rural*	*4461*	*11*	*15*	*Yes*	*Yes*	*FFQ*	*Nationally representative*
GDD 2017	Health Behavior in School-Aged Children 1990	1989	1990	Hungary	*Both urban and rural*	*6498*	*11*	*17*	*Yes*	*Yes*	*FFQ*	*Nationally representative*
GDD 2017	Health Behavior in School-Aged Children 1998	1998	1998	Hungary	*Both urban and rural*	*3601*	*11*	*17*	*Yes*	*Yes*	*FFQ*	*Nationally representative*
GDD 2017	Health Behavior in School-Aged Children 2002	2001	2002	Hungary	*Both urban and rural*	*4057*	*11*	*17*	*Yes*	*Yes*	*FFQ*	*Nationally representative*
GDD 2017	Health Behavior in School-Aged Children 2006	2006	2006	Hungary	*Both urban and rural*	*3472*	*11*	*15*	*Yes*	*Yes*	*FFQ*	*Nationally representative*
GDD 2017	Health Behavior in School-Aged Children 2010	2010	2010	Hungary	*Both urban and rural*	*4767*	*11*	*15*	*Yes*	*Yes*	*FFQ*	*Nationally representative*
GDD 2010	The First Hungarian Representative Nutrition Survey	1985	1988	Hungary	*Information not reported*	*1000*	*15*	*100*	*Yes*	*Yes*	*Multiple 24-hour recall*	*Nationally representative*

GDD 2010	Hungarian Community Health and Nutrition Survey	1991	1991	Hungary	*Information not reported*	*1000*	*0*	*100*	*Yes*	*Yes*	*Household availability/ budget survey*	*Nationally representative*
GDD 2010	The 2nd National Hungarian Survey	1992	1994	Hungary	*Information not reported*	*1000*	*18*	*100*	*Yes*	*Yes*	*Multiple 24-hour recall*	*Nationally representative*
GDD 2010	The 3rd National Hungarian Survey	2003	2003	Hungary	*Information not reported*	*1000*	*18*	*100*	*Yes*	*Yes*	*Multiple 24-hour recall*	*Nationally representative*
GDD 2010	The 3rd National Hungarian Survey	2003	2004	Hungary	*Information not reported*	*1000*	*18*	*64*	*Yes*	*Yes*	*Multiple 24-hour recall*	*Nationally representative*
GDD 2010	The 3rd National Hungarian Survey	2005	2006	Hungary	*Information not reported*	*100*	*11*	*14*	*Yes*	*Yes*	*Multiple 24-hour recall*	*Nationally representative*
GDD 2010	The 3rd National Hungarian Survey	2003	2004	Hungary	*Information not reported*	*1000*	*18*	*100*	*Yes*	*Yes*	*Multiple 24-hour recall*	*Nationally representative*
GDD 2010	The 3rd National Hungarian Survey	2003	2004	Hungary	*Information not reported*	*250*	*60*	*100*	*Yes*	*Yes*	*Multiple 24-hour recall*	*Nationally representative*
GDD 2010	INTERSALT, Porcsalam village	1985	1987	Hungary	*Information not reported*	*25*	*20*	*59*	*Yes*	*Yes*	*Biomarker*	*Locally representative*
GDD 2017	Health Behavior in School-Aged Children 2006	2006	2006	Iceland	*Both urban and rural*	*9444*	*11*	*15*	*Yes*	*Yes*	*FFQ*	*Nationally representative*
GDD 2017	Health Behavior in School-Aged Children 2010	2010	2010	Iceland	*Both urban and rural*	*11013*	*11*	*15*	*Yes*	*Yes*	*FFQ*	*Nationally representative*
GDD 2010	Dietary Survey of the Icelanders	1990	1990	Iceland	*Information not reported*	*1000*	*15*	*80*	*Yes*	*Yes*	*Single 24-hour recall*	*Nationally representative*
GDD 2010	Diet of Icelandic schoolchildren	1992	1993	Iceland	*Information not reported*	*1000*	*9*	*15*	*Yes*	*Yes*	*Single 24-hour recall*	*Nationally representative*
GDD 2010	Research on infant nutrition in Iceland	1995	1997	Iceland	*Information not reported*	*100*	*1*	*1*	*Yes*	*Yes*	*Multiple 24-hour recall*	*Nationally representative*
GDD 2010	The Diet of Icelanders, Dietary Survey of The Icelandic Nutrition Council 2002	2002	2002	Iceland	*Information not reported*	*1000*	*15*	*80*	*Yes*	*Yes*	*Single 24-hour recall*	*Nationally representative*
GDD 2010	Diet of Icelandic 9- and 15-year-old children and adolescents	2003	2004	Iceland	*Information not reported*	*250*	*9*	*15*	*Yes*	*Yes*	*Multiple 24-hour recall*	*Nationally representative*
GDD 2010	The diet of Icelandic infants	2005	2007	Iceland	*Information not reported*	*100*	*1*	*1*	*Yes*	*Yes*	*Multiple 24-hour recall*	*Nationally representative*
GDD 2010	TRANSFAIR study	1990	1990	Iceland	*Information not reported*	*250*	*19*	*64*	*Yes*	*Yes*	*Single 24-hour recall*	*Nationally representative*
GDD 2010	INTERSALT, Reykajavik and district	1985	1987	Iceland	*Information not reported*	*25*	*20*	*59*	*Yes*	*Yes*	*Biomarker*	*Sub-nationally representative*
GDD 2010	Olafsdottir	2003	2003	Iceland	*Information not reported*	*60*	*25*	*53*	*No*	*Yes*	*Biomarker*	*Sub-nationally representative*

GDD 2010	Olfasdottir et al.	2003	2003	Iceland	*Information not reported*	*60*	*25*	*55*	*No*	*Yes*	*Single 24-hour recall*	*Sub-nationally representative*
GDD 2017	Micronutrient status of rural and tribal nonpregnant women	2007	2007	India	*Both urban and rural*	*109*	*18*	*30*	*No*	*Yes*	*Single 24-hour recall*	*Sub-nationally representative*
GDD 2017	Micronutrient status of tribal pregnant women	2008	2009	India	*Only rural*	*440*	*18*	*32*	*No*	*Yes*	*Multiple 24-hour recall*	*Locally representative*
GDD 2017	Urban School girls	2005	2007	India	*Only urban*	*608*	*10*	*16*	*No*	*Yes*	*Multiple 24-hour recall*	*Locally representative*
GDD 2017	Urban children	2008	2009	India	*Only urban*	*935*	*2*	*18*	*Yes*	*Yes*	*Single 24-hour recall*	*Locally representative*
GDD 2017	Nutrition and health of adults	1998	2000	India	*Both urban and rural*	*629*	*18*	*65*	*Yes*	*Yes*	*FFQ*	*Locally representative*
GDD 2017	Prevention of type 2 diabetes in women with pre-diabetes using vitamin D supplementation and lifestyle intervention in North India (PREVENT-WIN Study)	2013	2017	India	*Only urban*	*134*	*20*	*60*	*No*	*Yes*	*Single 24-hour recall*	*Locally representative*
GDD 2017	Prevalence of Coronary Heart Disease (CHD) and Its Risk Factors in Residents of Urban and Rural Areas of National Capital Region – A Repeat Survey	2010	2012	India	*Only rural*	*2604*	*30*	*105*	*Yes*	*Yes*	*FFQ*	*Locally representative*
GDD 2017	Urban School Children Survey	2012	2012	India	*Only urban*	*1405*	*6*	*15*	*Yes*	*Yes*	*FFQ*	*Locally representative*
GDD 2017	Risk of Jowar Consumption associated with severity of Dental Fluorosis in North Karnataka India	2006	2009	India	*Only rural*	*428*	*12*	*15*	*Yes*	*Yes*	*FFQ*	*Locally representative*
GDD 2017	Formative Research on Improved Maternal Nutrition (MN), Infant and Young Child (IYCF) and Household Sanitation and Hygiene (HSH) Practices in UP, 2016	2016	2016	India	*Both urban and rural*	*360*	*1*	*38*	*Yes*	*Yes*	*FFQ*	*Sub-nationally representative*
GDD 2017	Dietary Survey	2013	2014	India	*Only rural*	*372*	*18*	*60*	*No*	*No*	*Single 24-hour recall*	*Locally representative*

GDD 2017	Attitudes and perceptions towards diet among children	2007	2008	India	*Only urban*	*201*	*9*	*16*	*Yes*	*Yes*	*Multiple 24-hour recall*	*Locally representative*
GDD 2017	Indian Multicentre Urban Survey	2011	2012	India	*Only urban*	*791*	*2*	*18*	*Yes*	*Yes*	*Single 24-hour recall*	*Nationally representative*
GDD 2017	Demographic and Health Survey 1998	1998	1999	India	*Both urban and rural*	*241716*	*0*	*49*	*Yes*	*Yes*	*DHS questionnaire*	*Nationally representative*
GDD 2017	Demographic and Health Survey 2005	2005	2006	India	*Both urban and rural*	*235520*	*0*	*54*	*Yes*	*Yes*	*DHS questionnaire*	*Nationally representative*
GDD 2017	Global School-Based Student Health Survey	2007	2007	India	*Both urban and rural*	*8010*	*11*	*16*	*Yes*	*Yes*	*FFQ*	*Nationally representative*
GDD 2017	India World Health Study 2003	2003	2003	India	*Both urban and rural*	*8872*	*15*	*102*	*Yes*	*Yes*	*FFQ*	*Nationally representative*
GDD 2017	Study on Global Ageing and Adult Health	2007	2007	India	*Both urban and rural*	*11222*	*18*	*106*	*Yes*	*Yes*	*FFQ*	*Nationally representative*
GDD 2010	National Nutrition Monitoring Bureau (NNMB) Rural Surveys	1996	1997	India	*Information not reported*	*1000*	*20*	*100*	*Yes*	*Yes*	*Single 24-hour recall*	*Locally representative*
GDD 2010	National Nutrition Monitoring Bureau (NNMB) Rural Surveys	2000	2001	India	*Information not reported*	*1027*	*20*	*100*	*Yes*	*Yes*	*Single 24-hour recall*	*Locally representative*
GDD 2010	National Nutrition Monitoring Bureau (NNMB) Rural Surveys	2004	2006	India	*Information not reported*	*1238*	*20*	*100*	*Yes*	*Yes*	*Single 24-hour recall*	*Locally representative*
GDD 2010	INTERSALT,Stok, Ladakh	1985	1987	India	*Information not reported*	*25*	*20*	*59*	*Yes*	*Yes*	*Biomarker*	*Sub-nationally representative*
GDD 2010	INTERSALT, New Delhi	1985	1987	India	*Information not reported*	*25*	*20*	*59*	*Yes*	*Yes*	*Biomarker*	*Sub-nationally representative*
GDD 2010	Radhika et al.	2003	2003	India	*Information not reported*	*1902*	*20*	*79*	*Yes*	*Yes*	*FFQ*	*Sub-nationally representative*
GDD 2017	Cardiovascular disease risk of adult in west Sumatera, Indonesia	2006	2006	Indonesia	*Both urban and rural*	*800*	*18*	*90*	*Yes*	*Yes*	*FFQ*	*Sub-nationally representative*
GDD 2017	Child growth and its relevant factors	2000	2004	Indonesia	*Only rural*	*126*	*1*	*12*	*Yes*	*Yes*	*Multiple 24-hour recall*	*Locally representative*
GDD 2017	Double Burden of Nutrition in Indonesia	2006	2007	Indonesia	*Both urban and rural*	*928*	*2*	*75*	*Yes*	*Yes*	*FFQ*	*Locally representative*
GDD 2017	SEANUTS: South East Asian Nutrition Surveys	2010	2011	Indonesia	*Both urban and rural*	*3600*	*0*	*12*	*Yes*	*Yes*	*Single 24-hour recall*	*Nationally representative*

Miller V, *et al. BMJ Global Health* 2021; 6:e003585. doi: 10.1136/bmjgh-2020-003585

GDD 2017	Global Student Based Student Health Survey 2007	2007	2007	Indonesia	*Both urban and rural*	*3093*	*11*	*16*	*Yes*	*Yes*	*FFQ*	*Nationally representative*	
GDD 2017	Demographic and Health Survey Indonesia 2002	2002	2003	Indonesia	*Both urban and rural*	*9886*	*0*	*4*	*Yes*	*Yes*	*DHS questionnair e*	*Nationally representative*	
GDD 2017	Demographic and Health Survey Indonesia 2012	2012	2012	Indonesia	*Both urban and rural*	*8665*	*0*	*4*	*Yes*	*Yes*	*DHS questionnair e*	*Nationally representative*	
GDD 2017	Demographic and Health Survey Indonesia 1987	1987	1987	Indonesia	*Both urban and rural*	*4491*	*0*	*4*	*Yes*	*Yes*	*DHS questionnair e*	*Nationally representative*	
GDD 2017	Demographic and Health Survey Indonesia 1991	1991	1991	Indonesia	*Both urban and rural*	*10081*	*0*	*4*	*Yes*	*Yes*	*DHS questionnair e*	*Nationally representative*	
GDD 2017	Demographic and Health Survey Indonesia 2007	2007	2008	Indonesia	*Both urban and rural*	*22570*	*0*	*49*	*Yes*	*Yes*	*DHS questionnair e*	*Nationally representative*	
GDD 2010	The determinant factors of hypertension among adult in West Sumatra, Indonesia	2004	2004	Indonesia	*Information not reported*	*250*	*18*	*90*	*Yes*	*Yes*	*FFQ*	*Sub-nationally representative*	
GDD 2010	Mustafa et al. (1 of 2)	2003	2003	Indonesia	*Information not reported*	*15*	*25*	*55*	*No*	*Yes*	*Biomarker*	*Locally representative*	
GDD 2017	Isfahan Healthy Heart Program	2007	2007	Iran, Islamic Republic Of	*Both urban and rural*	*1490*	*15*	*100*	*Yes*	*Yes*	*Single 24-hour recall*	*Sub-nationally representative*	
GDD 2017	Prevalence of Obesity	2004	2005	Iran, Islamic Republic Of	*Only urban*	*300*	*15*	*70*	*Yes*	*Yes*	*FFQ*	*Sub-nationally representative*	
GDD 2017	Yazd Health Study	2014	2015	Iran, Islamic Republic Of	*Only urban*	*9876*	*1*	*5*	*Yes*	*Yes*	*FFQ*	*Sub-nationally representative*	
GDD 2017	Fluoride intake of 4 year old Iranian Children	1995	1996	Iran, Islamic Republic Of	*Both urban and rural*	*129*	*4*	*4*	*Yes*	*Yes*	*Multiple 24-hour recall*	*Nationally representative*	
GDD 2017	Third Surveillance of Risk Factors of Non-Communicable Diseases (SuRFNC-2007)	2007	2007	Iran, Islamic Republic Of	*Both urban and rural*	*5149*	*14*	*64*	*Yes*	*Yes*	*DHS questionnair e*	*Nationally representative*	
GDD 2017	The CASPIAN IV study	2012	2012	Iran, Islamic Republic Of	*Both urban and rural*	*13486*	*6*	*18*	*Yes*	*Yes*	*Single 24-hour recall*	*Nationally representative*	

GDD 2017	Dietary habits and gastric cancer risk in north	2005	2007	Iran, Islamic Republic Of	*Both urban and rural*	*304*	*37*	*84*	*Yes*	*Yes*	*FFQ*	*Sub-nationally representative*
GDD 2017	Tehran Lipid and Glucose Study	2005	2008	Iran, Islamic Republic Of	*Only urban*	*3425*	*3*	*84*	*Yes*	*Yes*	*FFQ*	*Locally representative*
GDD 2017	Tehran Lipid and Glucose Study	2009	2011	Iran, Islamic Republic Of	*Only urban*	*7214*	*3*	*87*	*Yes*	*Yes*	*FFQ*	*Locally representative*
GDD 2017	Tehran Lipid and Glucose Study	2012	2015	Iran, Islamic Republic Of	*Only urban*	*6530*	*3*	*90*	*Yes*	*Yes*	*FFQ*	*Locally representative*
GDD 2017	Fourth Surveillance of Risk Factors of Non-Communicable Diseases (SuRFNCD-2011)	2011	2011	Iran, Islamic Republic Of	*Both urban and rural*	*11599*	*4*	*70*	*Yes*	*Yes*	*FFQ*	*Nationally representative*
GDD 2017	The Influence of Environmental Factors on Gastric Cancer in the Northwest of Iran	2003	2005	Iran, Islamic Republic Of	*Both urban and rural*	*366*	*22*	*106*	*Yes*	*Yes*	*FFQ*	*Sub-nationally representative*
GDD 2010	National Comprehensive Study on Household Food Consumption Pattern and Nutritional Status I.R.IRAN, 2001-2003	2001	2003	Iran, Islamic Republic Of	*Information not reported*	*7158*	*0*	*100*	*Yes*	*Yes*	*FFQ*	*Nationally representative*
GDD 2010	Non communicable disease surveillance in Islamic Republic of Iran	2005	2005	Iran, Islamic Republic Of	*Information not reported*	*3533*	*15*	*64*	*Yes*	*Yes*	*Single 24-hour recall*	*Nationally representative*
GDD 2010	Comprehensive National Household Food Consumption Survey	1990	1995	Iran, Islamic Republic Of	*Information not reported*	*1000*	*0*	*100*	*Yes*	*Yes*	*Multiple 24-hour recall*	*Nationally representative*
GDD 2010	Consumption of trans fats and estimated effects on coronary heart disease in Iran	2001	2003	Iran, Islamic Republic Of	*Information not reported*	*1000*	*0*	*100*	*Yes*	*Yes*	*Multiple 24-hour recall*	*Nationally representative*
GDD 2010	Azizi et al. (1 of 2)	1998	1998	Iran, Islamic Republic Of	*Information not reported*	*340*	*20*	*79*	*Yes*	*Yes*	*FFQ*	*Sub-nationally representative*
GDD 2010	Azizi et al. (2 of 2)	1998	1998	Iran, Islamic Republic Of	*Information not reported*	*343*	*20*	*79*	*Yes*	*Yes*	*FFQ*	*Sub-nationally representative*

Miller V, *et al. BMJ Global Health* 2021; 6:e003585. doi: 10.1136/bmjgh-2020-003585

GDD 2010	Rahmani et al.	2000	2000	Iran, Islamic Republic Of	*Information not reported*	*644*	*20*	*79*	*Yes*	*Yes*	*FFQ*	*Sub-nationally representative*
GDD 2010	Rafiei et al.	2001	2001	Iran, Islamic Republic Of	*Information not reported*	*304*	*20*	*60*	*Yes*	*Yes*	*Biomarker*	*Sub-nationally representative*
GDD 2017	Global Student Based Student Health Survey 2012	2012	2012	Iraq	*Both urban and rural*	*2007*	*11*	*16*	*Yes*	*Yes*	*FFQ*	*Nationally representative*
GDD 2017	Health Behavior in School-Aged Children 2002	2001	2002	Ireland	*Both urban and rural*	*2875*	*11*	*16*	*Yes*	*Yes*	*FFQ*	*Nationally representative*
GDD 2017	Health Behavior in School-Aged Children 2006	2006	2006	Ireland	*Both urban and rural*	*4806*	*11*	*15*	*Yes*	*Yes*	*FFQ*	*Nationally representative*
GDD 2017	Health Behavior in School-Aged Children 2010	2010	2010	Ireland	*Both urban and rural*	*4655*	*11*	*15*	*Yes*	*Yes*	*FFQ*	*Nationally representative*
GDD 2017	Health Behavior in School-Aged Children 1998	1997	1998	Ireland	*Both urban and rural*	*4394*	*11*	*16*	*Yes*	*Yes*	*FFQ*	*Nationally representative*
GDD 2010	Irish Community Health and Nutrition Survey	1987	1987	Ireland	*Information not reported*	*1000*	*0*	*100*	*Yes*	*Yes*	*Household availability/ budget survey*	*Nationally representative*
GDD 2010	Irish Community Health and Nutrition Survey	1994	1994	Ireland	*Information not reported*	*1000*	*0*	*100*	*Yes*	*Yes*	*Household availability/ budget survey*	*Nationally representative*
GDD 2010	North South Ireland Food Consumption Survey (NSIFCS)	1997	1999	Ireland	*Information not reported*	*1000*	*18*	*64*	*Yes*	*Yes*	*Multiple 24-hour recall*	*Nationally representative*
GDD 2010	SL�N, 2007 & SL�N 1997/1998	2005	2006	Ireland	*Information not reported*	*250*	*13*	*17*	*Yes*	*Yes*	*Multiple 24-hour recall*	*Nationally representative*
GDD 2010	SL�N, 2007 & SL�N 1997/1998	1999	2007	Ireland	*Information not reported*	*1000*	*18*	*64*	*Yes*	*Yes*	*Multiple 24-hour recall*	*Nationally representative*
GDD 2010	SL�N, 2007 & SL�N 1997/1998	2007	2007	Ireland	*Information not reported*	*1000*	*65*	*100*	*Yes*	*Yes*	*Multiple 24-hour recall*	*Nationally representative*
GDD 2010	Irish Community Health and Nutrition Survey	1999	1999	Ireland	*Information not reported*	*1000*	*0*	*100*	*Yes*	*Yes*	*Household availability/ budget survey*	*Nationally representative*
GDD 2010	National Children�s Food Survey, 2005	2003	2004	Ireland	*Information not reported*	*250*	*5*	*9*	*Yes*	*Yes*	*Multiple 24-hour recall*	*Nationally representative*

GDD 2010	The National Teen�s food Survey, 2008	2003	2004	Ireland	*Information not reported*	*109*	*10*	*12*	*Yes*	*Yes*	*Multiple 24-hour recall*	*Nationally representative*
GDD 2010	Flynn et al.	1987	1987	Ireland	*Information not reported*	*46*	*20*	*60*	*Yes*	*Yes*	*Biomarker*	*Sub-nationally representative*
GDD 2017	Mabat First National Health and Nutrition Survey	1999	2001	Israel	*Both urban and rural*	*3242*	*25*	*64*	*Yes*	*Yes*	*Single 24-hour recall*	*Nationally representative*
GDD 2017	Mabat Youth National Health and Nutrition Survey	2003	2004	Israel	*Both urban and rural*	*577*	*12*	*19*	*Yes*	*Yes*	*Single 24-hour recall*	*Nationally representative*
GDD 2017	Mabat Elderly National Health and Nutrition Survey	2005	2006	Israel	*Only urban*	*1786*	*63*	*96*	*Yes*	*Yes*	*Single 24-hour recall*	*Nationally representative*
GDD 2017	Health Behavior in School-Aged Children 1994	1993	1994	Israel	*Both urban and rural*	*4299*	*11*	*16*	*Yes*	*Yes*	*FFQ*	*Nationally representative*
GDD 2017	Health Behavior in School-Aged Children 1998	1998	1998	Israel	*Both urban and rural*	*4746*	*11*	*17*	*Yes*	*Yes*	*FFQ*	*Nationally representative*
GDD 2017	Health Behavior in School-Aged Children 2002	2001	2002	Israel	*Both urban and rural*	*5341*	*11*	*17*	*Yes*	*Yes*	*FFQ*	*Nationally representative*
GDD 2017	Health Behavior in School-Aged Children 2006	2006	2006	Israel	*Both urban and rural*	*5249*	*11*	*15*	*Yes*	*Yes*	*FFQ*	*Nationally representative*
GDD 2017	Health Behavior in School-Aged Children 2010	2010	2010	Israel	*Both urban and rural*	*4098*	*11*	*15*	*Yes*	*Yes*	*FFQ*	*Nationally representative*
GDD 2017	Mediterranean healthy Eating, Aging and Lifestyles (MEAL) study	2014	2016	Italy	*Only urban*	*1957*	*15*	*100*	*Yes*	*Yes*	*FFQ*	*Locally representative*
GDD 2017	Moli-Sani Study	2005	2010	Italy	*Both urban and rural*	*23369*	*35*	*100*	*Yes*	*Yes*	*FFQ*	*Sub-nationally representative*
GDD 2017	Health Behavior in School-Aged Children 2006	2005	2006	Italy	*Both urban and rural*	*3911*	*11*	*15*	*Yes*	*Yes*	*FFQ*	*Nationally representative*
GDD 2017	Health Behavior in School-Aged Children 2010	2009	2010	Italy	*Both urban and rural*	*4806*	*11*	*15*	*Yes*	*Yes*	*FFQ*	*Nationally representative*
GDD 2017	Health Behavior in School-Aged Children 2002	2001	2002	Italy	*Both urban and rural*	*4357*	*11*	*17*	*Yes*	*Yes*	*FFQ*	*Nationally representative*
GDD 2010	INN 1980-84	1980	1984	Italy	*Information not reported*	*1000*	*1*	*80*	*Yes*	*Yes*	*Multiple 24-hour recall*	*Nationally representative*

GDD 2010	Italian Community Health and Nutrition Survey	1990	1990	Italy	*Information not reported*	*1000*	*0*	*100*	*Yes*	*Yes*	*Household availability/ budget survey*	*Nationally representative*
GDD 2010	Italian Community Health and Nutrition Survey	1993	1993	Italy	*Information not reported*	*1000*	*0*	*100*	*Yes*	*Yes*	*Household availability/ budget survey*	*Nationally representative*
GDD 2010	INN-CA 1994-96	1994	1996	Italy	*Information not reported*	*1000*	*20*	*100*	*Yes*	*Yes*	*Multiple 24-hour recall*	*Nationally representative*
GDD 2010	INN-CA 1994-96	1994	1996	Italy	*Information not reported*	*1000*	*4*	*100*	*Yes*	*Yes*	*Multiple 24-hour recall*	*Nationally representative*
GDD 2010	Italian Community Health and Nutrition Survey	1996	1996	Italy	*Information not reported*	*1000*	*0*	*100*	*Yes*	*Yes*	*Household availability/ budget survey*	*Nationally representative*
GDD 2010	INRAN-SCAI 2005-06	2005	2006	Italy	*Information not reported*	*1000*	*20*	*100*	*Yes*	*Yes*	*Multiple 24-hour recall*	*Nationally representative*
GDD 2010	TRANSFAIR study	1980	1984	Italy	*Information not reported*	*1000*	*1*	*80*	*Yes*	*Yes*	*Household availability/ budget survey*	*Nationally representative*
GDD 2010	INTERSALT, Bassiano	1985	1987	Italy	*Information not reported*	*25*	*20*	*59*	*Yes*	*Yes*	*Biomarker*	*Sub-nationally representative*
GDD 2010	INTERSALT, Gubbio	1985	1987	Italy	*Information not reported*	*25*	*20*	*59*	*Yes*	*Yes*	*Biomarker*	*Sub-nationally representative*
GDD 2010	INTERSALT, Mirano	1985	1987	Italy	*Information not reported*	*25*	*20*	*59*	*Yes*	*Yes*	*Biomarker*	*Sub-nationally representative*
GDD 2010	INTERSALT, Naples	1985	1987	Italy	*Information not reported*	*25*	*20*	*59*	*Yes*	*Yes*	*Biomarker*	*Sub-nationally representative*
GDD 2010	Pavan et al. (1 of 4)	1994	1994	Italy	*Information not reported*	*370*	*22*	*89*	*Yes*	*Yes*	*FFQ*	*Sub-nationally representative*
GDD 2010	Venezia et al.	2002	2004	Italy	*Information not reported*	*940*	*25*	*75*	*Yes*	*No*	*Biomarker*	*Sub-nationally representative*
GDD 2017	Nutrition in African Population	1993	1996	Jamaica	*Only rural*	*846*	*24*	*74*	*Yes*	*Yes*	*FFQ*	*Nationally representative*
GDD 2017	Global Student Based Student Health Survey 2010	2010	2010	Jamaica	*Both urban and rural*	*1586*	*11*	*16*	*Yes*	*Yes*	*FFQ*	*Nationally representative*
GDD 2010	Social and dietary determinants of body max index of adult Jamaicans	1993	1995	Jamaica	*Information not reported*	*500*	*25*	*74*	*Yes*	*Yes*	*FFQ*	*Nationally representative*
GDD 2010	Cooper et al.	1994	1995	Jamaica	*Information not reported*	*1357*	*25*	*74*	*Yes*	*Yes*	*Biomarker*	*Sub-nationally representative*

Miller V, *et al. BMJ Global Health* 2021; 6:e003585. doi: 10.1136/bmjgh-2020-003585

GDD 2017	Japan Public Health Center-based prospective Study (JPHC) Cohort I	1990	1995	Japan	*Both urban and rural*	*42115*	*45*	*65*	*Yes*	*Yes*	*FFQ*	*Locally representative*
GDD 2017	Japan Public Health Center-based prospective Study (JPHC) Cohort I	1990	1995	Japan	*Both urban and rural*	*41536*	*45*	*75*	*Yes*	*Yes*	*FFQ*	*Locally representative*
GDD 2017	Japan Public Health Center-based prospective Study (JPHC Study) Cohort II	1993	2000	Japan	*Both urban and rural*	*55720*	*45*	*75*	*Yes*	*Yes*	*FFQ*	*Sub-nationally representative*
GDD 2017	Japan Public Health Center-based prospective Study (JPHC) Cohort II	1993	2000	Japan	*Both urban and rural*	*50766*	*45*	*85*	*Yes*	*Yes*	*FFQ*	*Sub-nationally representative*
GDD 2017	National Health and Nutrition Survey	1995	1995	Japan	*Both urban and rural*	*14240*	*1*	*100*	*Yes*	*Yes*	*Single 24-hour recall*	*Nationally representative*
GDD 2017	National Health and Nutrition Survey	1996	1996	Japan	*Both urban and rural*	*14019*	*1*	*100*	*Yes*	*Yes*	*Single 24-hour recall*	*Nationally representative*
GDD 2017	National Health and Nutrition Survey	1997	1997	Japan	*Both urban and rural*	*13289*	*1*	*100*	*Yes*	*Yes*	*Single 24-hour recall*	*Nationally representative*
GDD 2017	National Health and Nutrition Survey	1998	1998	Japan	*Both urban and rural*	*14158*	*1*	*100*	*Yes*	*Yes*	*Single 24-hour recall*	*Nationally representative*
GDD 2017	National Health and Nutrition Survey	1999	1999	Japan	*Both urban and rural*	*12590*	*1*	*100*	*Yes*	*Yes*	*Single 24-hour recall*	*Nationally representative*
GDD 2017	National Health and Nutrition Survey	2000	2000	Japan	*Both urban and rural*	*12271*	*1*	*100*	*Yes*	*Yes*	*Single 24-hour recall*	*Nationally representative*
GDD 2017	National Health and Nutrition Survey	2001	2001	Japan	*Both urban and rural*	*12481*	*1*	*100*	*Yes*	*Yes*	*Single 24-hour recall*	*Nationally representative*
GDD 2017	National Health and Nutrition Survey	2002	2002	Japan	*Both urban and rural*	*11491*	*1*	*100*	*Yes*	*Yes*	*Single 24-hour recall*	*Nationally representative*
GDD 2017	National Health and Nutrition Survey	2003	2003	Japan	*Both urban and rural*	*11104*	*1*	*100*	*Yes*	*Yes*	*Single 24-hour recall*	*Nationally representative*
GDD 2017	National Health and Nutrition Survey	2004	2004	Japan	*Both urban and rural*	*8762*	*1*	*100*	*Yes*	*Yes*	*Single 24-hour recall*	*Nationally representative*
GDD 2017	National Health and Nutrition Survey	2005	2005	Japan	*Both urban and rural*	*4891*	*1*	*100*	*Yes*	*Yes*	*Single 24-hour recall*	*Nationally representative*
GDD 2017	National Health and Nutrition Survey	2006	2006	Japan	*Both urban and rural*	*9423*	*1*	*100*	*Yes*	*Yes*	*Single 24-hour recall*	*Nationally representative*
GDD 2017	National Health and Nutrition Survey	2007	2007	Japan	*Both urban and rural*	*8885*	*1*	*100*	*Yes*	*Yes*	*Single 24-hour recall*	*Nationally representative*
GDD 2017	National Health and Nutrition Survey	2008	2008	Japan	*Both urban and rural*	*9127*	*1*	*100*	*Yes*	*Yes*	*Single 24-hour recall*	*Nationally representative*
GDD 2017	National Health and Nutrition Survey	2009	2009	Japan	*Both urban and rural*	*9004*	*1*	*100*	*Yes*	*Yes*	*Single 24-hour recall*	*Nationally representative*
GDD 2017	National Health and Nutrition Survey	2010	2010	Japan	*Both urban and rural*	*9566*	*1*	*100*	*Yes*	*Yes*	*Single 24-hour recall*	*Nationally representative*

GDD 2017	National Health and Nutrition Survey	2011	2011	Japan	*Both urban and rural*	*14466*	*1*	*100*	*Yes*	*Yes*	*Single 24-hour recall*	*Nationally representative*
GDD 2017	National Health and Nutrition Survey	2012	2012	Japan	*Both urban and rural*	*32227*	*1*	*100*	*Yes*	*Yes*	*Single 24-hour recall*	*Nationally representative*
GDD 2017	National Health and Nutrition Survey	2013	2013	Japan	*Both urban and rural*	*13858*	*1*	*100*	*Yes*	*Yes*	*Single 24-hour recall*	*Nationally representative*
GDD 2017	National Health and Nutrition Survey	2014	2014	Japan	*Both urban and rural*	*14348*	*1*	*100*	*Yes*	*Yes*	*Single 24-hour recall*	*Nationally representative*
GDD 2017	National Health and Nutrition Survey	2015	2015	Japan	*Both urban and rural*	*13424*	*1*	*100*	*Yes*	*Yes*	*Single 24-hour recall*	*Nationally representative*
GDD 2010	The Japan Public Health Center-based Prospective Study (JPHC Study)	1995	1998	Japan	*Information not reported*	*18943*	*45*	*74*	*Yes*	*Yes*	*FFQ*	*Locally representative*
GDD 2010	National Nutrition Survey (in Japan)	1995	1995	Japan	*Information not reported*	*1000*	*20*	*100*	*Yes*	*Yes*	*Single 24-hour recall*	*Nationally representative*
GDD 2010	National Nutrition Survey (in Japan)	1998	1998	Japan	*Information not reported*	*1000*	*20*	*100*	*Yes*	*Yes*	*Single 24-hour recall*	*Nationally representative*
GDD 2010	The Japan Public Health Center-based Prospective Study (JPHC Study)	2000	2003	Japan	*Information not reported*	*10704*	*50*	*79*	*Yes*	*Yes*	*FFQ*	*Locally representative*
GDD 2010	INTERSALT, Osaka	1985	1987	Japan	*Information not reported*	*25*	*20*	*59*	*Yes*	*Yes*	*Biomarker*	*Sub-nationally representative*
GDD 2010	INTERSALT, Tochigi	1985	1987	Japan	*Information not reported*	*25*	*20*	*59*	*Yes*	*Yes*	*Biomarker*	*Sub-nationally representative*
GDD 2010	INTERSALT, Toyama	1985	1987	Japan	*Information not reported*	*25*	*20*	*59*	*Yes*	*Yes*	*Biomarker*	*Sub-nationally representative*
GDD 2010	Kawamura et al.	1993	1994	Japan	*Information not reported*	*90*	*30*	*65*	*Yes*	*Yes*	*Biomarker*	*Sub-nationally representative*
GDD 2010	Liu et al.	1984	1999	Japan	*Information not reported*	*484*	*48*	*56*	*Yes*	*Yes*	*Biomarker*	*Nationally representative*
GDD 2010	INTERMAP, Toyama	1997	1998	Japan	*Information not reported*	*72*	*40*	*59*	*Yes*	*Yes*	*Biomarker*	*Sub-nationally representative*
GDD 2010	INTERMAP, Sapporo	1997	1998	Japan	*Information not reported*	*75*	*40*	*59*	*Yes*	*Yes*	*Biomarker*	*Sub-nationally representative*
GDD 2010	INTERMAP, Aito Town	1997	1998	Japan	*Information not reported*	*68*	*40*	*59*	*Yes*	*Yes*	*Biomarker*	*Sub-nationally representative*
GDD 2010	INTERMAP, Wakayama	1997	1998	Japan	*Information not reported*	*73*	*40*	*59*	*Yes*	*Yes*	*Biomarker*	*Sub-nationally representative*
GDD 2010	INTERMAP, Toyama	1997	1998	Japan	*Information not reported*	*72*	*40*	*59*	*Yes*	*Yes*	*Single 24-hour recall*	*Sub-nationally representative*
GDD 2010	INTERMAP, Sapporo	1997	1998	Japan	*Information not reported*	*75*	*40*	*59*	*Yes*	*Yes*	*Single 24-hour recall*	*Sub-nationally representative*
GDD 2010	INTERMAP, Aito Town	1997	1998	Japan	*Information not reported*	*68*	*40*	*59*	*Yes*	*Yes*	*Single 24-hour recall*	*Sub-nationally representative*

GDD 2010	INTERMAP, Wakayama	1997	1998	Japan	*Information not reported*	*73*	*40*	*59*	*Yes*	*Yes*	*Single 24-hour recall*	*Sub-nationally representative*
GDD 2010	Sasaki et al.	1996	1996	Japan	*Information not reported*	*154*	*20*	*25*	*Yes*	*Yes*	*Biomarker*	*Sub-nationally representative*
GDD 2010	Sasaki et al.	1996	1996	Japan	*Information not reported*	*154*	*20*	*25*	*Yes*	*Yes*	*Single 24-hour recall*	*Sub-nationally representative*
GDD 2010	Kimira et al.	1997	2002	Japan	*Information not reported*	*219*	*27*	*84*	*Yes*	*Yes*	*Biomarker*	*Sub-nationally representative*
GDD 2010	Kimira et al.	1997	2002	Japan	*Information not reported*	*219*	*27*	*84*	*Yes*	*Yes*	*Single 24-hour recall*	*Sub-nationally representative*
GDD 2017	Case Control Study	2010	2012	Jordan	*Both urban and rural*	*294*	*22*	*80*	*Yes*	*Yes*	*FFQ*	*Locally representative*
GDD 2017	Obesity and Macro/micronutrients	2014	2015	Jordan	*Only urban*	*153*	*18*	*53*	*Yes*	*Yes*	*FFQ*	*Locally representative*
GDD 2017	Global Student Based Student Health Survey 2004	2004	2004	Jordan	*Both urban and rural*	*2409*	*11*	*16*	*Yes*	*Yes*	*FFQ*	*Nationally representative*
GDD 2017	Global Student Based Student Health Survey 2007	2007	2007	Jordan	*Both urban and rural*	*2173*	*11*	*16*	*Yes*	*Yes*	*FFQ*	*Nationally representative*
GDD 2017	Global Student Based Student Health Survey 2010	2010	2010	Jordan	*Both urban and rural*	*1480*	*11*	*16*	*Yes*	*Yes*	*FFQ*	*Nationally representative*
GDD 2017	Demographic and Health Survey Jordan 2002	2002	2002	Jordan	*Both urban and rural*	*6741*	*0*	*49*	*Yes*	*Yes*	*DHS questionnaire*	*Nationally representative*
GDD 2017	Demographic and Health Survey Jordan 2007	2007	2007	Jordan	*Both urban and rural*	*16991*	*0*	*49*	*Yes*	*Yes*	*DHS questionnaire*	*Nationally representative*
GDD 2017	Demographic and Health Survey Jordan 2012	2012	2012	Jordan	*Both urban and rural*	*26984*	*0*	*96*	*Yes*	*Yes*	*DHS questionnaire*	*Nationally representative*
GDD 2017	Demographic and Health Survey Jordan 2009	2009	2009	Jordan	*Both urban and rural*	*15791*	*0*	*95*	*Yes*	*Yes*	*DHS questionnaire*	*Nationally representative*
GDD 2010	Jordan Behavioral risk factor Surveillance System (JBRFSS)	2007	2007	Jordan	*Information not reported*	*1000*	*20*	*100*	*Yes*	*Yes*	*FFQ*	*Nationally representative*
GDD 2017	Demographic and Health Survey Kazakhstan 1995	1995	1995	Kazakhstan	*Both urban and rural*	*4444*	*0*	*49*	*Yes*	*Yes*	*DHS questionnaire*	*Nationally representative*
GDD 2017	World Health Survey 2003	2003	2003	Kazakhstan	*Both urban and rural*	*4117*	*18*	*89*	*Yes*	*Yes*	*FFQ*	*Nationally representative*

GDD 2017	Demographic and Health Survey Kazakhstan 1999	1999	1999	Kazakhstan	*Both urban and rural*	*4003*	*0*	*59*	*Yes*	*Yes*	*DHS questionnaire*	*Nationally representative*
GDD 2010	Living Conditions Lifestyles and Health	2001	2002	Kazakhstan	*Information not reported*	*1000*	*18*	*99*	*Yes*	*Yes*	*FFQ*	*Nationally representative*
GDD 2017	Nutrition status and associated factors among children in public primary schools in Dagoretti, Nairobi, Kenya	2009	2010	Kenya	*Only urban*	*416*	*4*	*11*	*Yes*	*Yes*	*Single 24-hour recall*	*Locally representative*
GDD 2017	Global Student Based Student Health Survey 2003	2003	2003	Kenya	*Both urban and rural*	*3573*	*11*	*16*	*Yes*	*Yes*	*FFQ*	*Nationally representative*
GDD 2017	World Health Survey 2003	2003	2003	Kenya	*Both urban and rural*	*4002*	*18*	*100*	*Yes*	*Yes*	*FFQ*	*Nationally representative*
GDD 2017	Demographic and Health Survey Kenya 2003	2003	2003	Kenya	*Both urban and rural*	*4439*	*0*	*4*	*Yes*	*Yes*	*DHS questionnaire*	*Nationally representative*
GDD 2017	Demographic and Health Survey Kenya 2008	2008	2009	Kenya	*Both urban and rural*	*4676*	*0*	*4*	*Yes*	*Yes*	*DHS questionnaire*	*Nationally representative*
GDD 2017	Demographic and Health Survey Kenya 2014	2014	2014	Kenya	*Both urban and rural*	*5766*	*0*	*4*	*Yes*	*Yes*	*DHS questionnaire*	*Nationally representative*
GDD 2010	INTERSALT, Rambugu and Ndori villages	1985	1987	Kenya	*Information not reported*	*23*	*20*	*59*	*Yes*	*Yes*	*Biomarker*	*Locally representative*
GDD 2017	Global Student Based Student Health Survey 2011	2011	2011	Kiribati	*Both urban and rural*	*1559*	*11*	*16*	*Yes*	*Yes*	*FFQ*	*Nationally representative*
GDD 2010	Kiribati NCD Risk Factors STEPS Report	2004	2004	Kiribati	*Information not reported*	*1000*	*15*	*64*	*Yes*	*Yes*	*FFQ*	*Nationally representative*
GDD 2017	Korea National Health and Nutrition Examination Survey	1998	1998	Korea	*Both urban and rural*	*11321*	*1*	*98*	*Yes*	*Yes*	*FFQ*	*Nationally representative*
GDD 2017	Korea National Health and Nutrition Examination Survey	2001	2001	Korea	*Both urban and rural*	*10075*	*1*	*99*	*Yes*	*Yes*	*FFQ*	*Nationally representative*
GDD 2017	Korea National Health and Nutrition Examination Survey	2005	2005	Korea	*Both urban and rural*	*8983*	*1*	*103*	*Yes*	*Yes*	*FFQ*	*Nationally representative*
GDD 2017	Korea National Health and Nutrition Examination Survey	2007	2007	Korea	*Both urban and rural*	*4096*	*1*	*103*	*Yes*	*Yes*	*FFQ*	*Nationally representative*

GDD 2017	Korea National Health and Nutrition Examination Survey	2008	2008	Korea	*Both urban and rural*	*8632*	*1*	*93*	*Yes*	*Yes*	*FFQ*	*Nationally representative*
GDD 2017	Korea National Health and Nutrition Examination Survey	2009	2009	Korea	*Both urban and rural*	*9393*	*1*	*95*	*Yes*	*Yes*	*FFQ*	*Nationally representative*
GDD 2017	Korea National Health and Nutrition Examination Survey	2010	2010	Korea	*Both urban and rural*	*8022*	*1*	*98*	*Yes*	*Yes*	*FFQ*	*Nationally representative*
GDD 2017	Korea National Health and Nutrition Examination Survey	2011	2011	Korea	*Both urban and rural*	*7706*	*1*	*97*	*Yes*	*Yes*	*FFQ*	*Nationally representative*
GDD 2017	Korea National Health and Nutrition Examination Survey	2012	2012	Korea	*Both urban and rural*	*7209*	*1*	*98*	*Yes*	*Yes*	*FFQ*	*Nationally representative*
GDD 2017	Korea National Health and Nutrition Examination Survey	2013	2013	Korea	*Both urban and rural*	*7244*	*1*	*96*	*Yes*	*Yes*	*FFQ*	*Nationally representative*
GDD 2017	Korea National Health and Nutrition Examination Survey	2014	2014	Korea	*Both urban and rural*	*6802*	*1*	*101*	*Yes*	*Yes*	*FFQ*	*Nationally representative*
GDD 2010	World-wide consumption of trans fatty acids			Korea	*Information not reported*		*0*	*100*	*Yes*	*Yes*	*Single 24-hour recall*	*Nationally representative*
GDD 2010	INTERSALT, Pusan	1985	1987	Korea	*Information not reported*	*25*	*20*	*59*	*Yes*	*Yes*	*Biomarker*	*Sub-nationally representative*
GDD 2017	Case-Control study of thyroid cancer in Kuwait	2001	2005	Kuwait	*Only urban*	*313*	*12*	*73*	*Yes*	*Yes*	*FFQ*	*Nationally representative*
GDD 2017	Global Student Based Student Health Survey 2011	2011	2011	Kuwait	*Both urban and rural*	*2648*	*11*	*16*	*Yes*	*Yes*	*FFQ*	*Nationally representative*
GDD 2010	Kuwait STEPS 2006	2006	2006	Kuwait	*Information not reported*	*1000*	*20*	*64*	*Yes*	*Yes*	*FFQ*	*Nationally representative*
GDD 2010	National Nutrition Survey for the State of Kuwait	2008	2009	Kuwait	*Information not reported*	*1000*	*3*	*100*	*Yes*	*Yes*	*Single 24-hour recall*	*Nationally representative*
GDD 2017	Demographic and Health Survey Kyrgyzstan 1997	1997	1997	Kyrgyzstan	*Both urban and rural*	*4792*	*0*	*49*	*Yes*	*Yes*	*DHS questionnair e*	*Nationally representative*
GDD 2017	Demographic and Health Survey Kyrgyzstan 2012	2012	2012	Kyrgyzstan	*Both urban and rural*	*12222*	*0*	*49*	*Yes*	*Yes*	*DHS questionnair e*	*Nationally representative*

GDD 2010	Living Conditions Lifestyles and Health	2001	2002	Kyrgyzstan	*Information not reported*	*1000*	*18*	*99*	*Yes*	*Yes*	*FFQ*	*Nationally representative*
GDD 2017	World Health Survey 2003	2003	2003	Lao People's Democratic Republic	*Both urban and rural*	*3367*	*17*	*95*	*Yes*	*Yes*	*FFQ*	*Nationally representative*
GDD 2010	STEP LAO	2008	2008	Lao People's Democratic Republic	*Information not reported*	*1000*	*25*	*64*	*Yes*	*Yes*	*FFQ*	*Sub-nationally representative*
GDD 2017	World Health Survey 2003	2003	2003	Latvia	*Both urban and rural*	*789*	*18*	*89*	*Yes*	*Yes*	*FFQ*	*Nationally representative*
GDD 2017	Health Behavior in School-Aged Children 1994	1993	1994	Latvia	*Both urban and rural*	*3749*	*11*	*17*	*Yes*	*Yes*	*FFQ*	*Nationally representative*
GDD 2017	Health Behavior in School-Aged Children 1998	1998	1998	Latvia	*Both urban and rural*	*3675*	*11*	*17*	*Yes*	*Yes*	*FFQ*	*Nationally representative*
GDD 2017	Health Behavior in School-Aged Children 2002	2001	2002	Latvia	*Both urban and rural*	*3455*	*11*	*16*	*Yes*	*Yes*	*FFQ*	*Nationally representative*
GDD 2017	Health Behavior in School-Aged Children 2006	2006	2006	Latvia	*Both urban and rural*	*4205*	*11*	*15*	*Yes*	*Yes*	*FFQ*	*Nationally representative*
GDD 2017	Health Behavior in School-Aged Children 2010	2010	2010	Latvia	*Both urban and rural*	*4238*	*11*	*15*	*Yes*	*Yes*	*FFQ*	*Nationally representative*
GDD 2010	Nutrition and Lifestyle in the Baltic Republics	1997	1997	Latvia	*Information not reported*	*1000*	*19*	*64*	*Yes*	*Yes*	*Single 24-hour recall*	*Nationally representative*
GDD 2010	Latvian Community Health and Nutrition Survey	2002	2002	Latvia	*Information not reported*	*1000*	*0*	*100*	*Yes*	*Yes*	*Household availability/ budget survey*	*Nationally representative*
GDD 2010	Latvian Community Health and Nutrition Survey	2003	2003	Latvia	*Information not reported*	*1000*	*0*	*100*	*Yes*	*Yes*	*Household availability/ budget survey*	*Nationally representative*
GDD 2010	Latvian Community Health and Nutrition Survey	2004	2004	Latvia	*Information not reported*	*1000*	*0*	*100*	*Yes*	*Yes*	*Household availability/ budget survey*	*Nationally representative*
GDD 2017	Socioeconomic and dietary determinants of obesity in Lebanon	2008	2009	Lebanon	*Both urban and rural*	*3037*	*7*	*90*	*Yes*	*Yes*	*FFQ*	*Nationally representative*

GDD 2017	Global Student Based Student Health Survey 2005	2005	2005	Lebanon	*Both urban and rural*	*5083*	*11*	*16*	*Yes*	*Yes*	*FFQ*	*Nationally representative*
GDD 2017	Global Student Based Student Health Survey 2011	2011	2011	Lebanon	*Both urban and rural*	*2272*	*11*	*16*	*Yes*	*Yes*	*FFQ*	*Nationally representative*
GDD 2017	Demographic and Health Survey Lesotho 2004	2004	2005	Lesotho	*Both urban and rural*	*5772*	*0*	*49*	*Yes*	*Yes*	*DHS questionnaire*	*Nationally representative*
GDD 2017	Demographic and Health Survey Lesotho 2009	2009	2010	Lesotho	*Both urban and rural*	*3347*	*0*	*47*	*Yes*	*Yes*	*DHS questionnaire*	*Nationally representative*
GDD 2017	Demographic and Health Survey Lesotho 2014	2014	2014	Lesotho	*Both urban and rural*	*5509*	*0*	*49*	*Yes*	*Yes*	*DHS questionnaire*	*Nationally representative*
GDD 2017	Demographic and Health Survey Libreria 2011	2011	2011	Liberia	*Both urban and rural*	*2449*	*0*	*4*	*Yes*	*Yes*	*Biomarker*	*Nationally representative*
GDD 2017	Demographic and Health Survey Liberia 2007	2006	2007	Liberia	*Both urban and rural*	*6897*	*0*	*49*	*Yes*	*Yes*	*DHS questionnaire*	*Nationally representative*
GDD 2017	Demographic and Health Survey Liberia 2009	2008	2009	Liberia	*Both urban and rural*	*3046*	*0*	*4*	*Yes*	*Yes*	*Biomarker*	*Nationally representative*
GDD 2017	Demographic and Health Survey Liberia 1986	1986	1986	Liberia	*Both urban and rural*	*1628*	*0*	*4*	*Yes*	*Yes*	*DHS questionnaire*	*Nationally representative*
GDD 2017	Global Student Based Student Health Survey 2007	2007	2007	Libyan Arab Jamahiriya	*Both urban and rural*	*2183*	*11*	*16*	*Yes*	*Yes*	*FFQ*	*Nationally representative*
GDD 2010	Libya Global School-based Student Health Survey (GSHS)-2007	2009	2009	Libyan Arab Jamahiriya	*Information not reported*	*1000*	*25*	*64*	*Yes*	*Yes*	*FFQ*	*Nationally representative*
GDD 2017	Health Behavior in School-Aged Children 2010	2010	2010	Lithuania	*Both urban and rural*	*5300*	*11*	*15*	*Yes*	*Yes*	*FFQ*	*Nationally representative*
GDD 2017	Health Behavior in School-Aged Children 1994	1993	1994	Lithuania	*Both urban and rural*	*5428*	*10*	*16*	*Yes*	*Yes*	*FFQ*	*Nationally representative*
GDD 2017	Health Behavior in School-Aged Children 1998	1998	1998	Lithuania	*Both urban and rural*	*4513*	*11*	*16*	*Yes*	*Yes*	*FFQ*	*Nationally representative*

Miller V, *et al. BMJ Global Health* 2021; 6:e003585. doi: 10.1136/bmjgh-2020-003585

GDD 2017	Health Behavior in School-Aged Children 2002	2001	2002	Lithuania	*Both urban and rural*	*5644*	*11*	*17*	*Yes*	*Yes*	*FFQ*	*Nationally representative*
GDD 2017	Health Behavior in School-Aged Children 2006	2006	2006	Lithuania	*Both urban and rural*	*5601*	*11*	*15*	*Yes*	*Yes*	*FFQ*	*Nationally representative*
GDD 2010	Nutrition and Lifestlye in the Baltic Republics	1997	1997	Lithuania	*Information not reported*	*1000*	*20*	*65*	*Yes*	*Yes*	*Single 24-hour recall*	*Nationally representative*
GDD 2010	No survey name available, unpubl. Data, data provided by the Institute od Biochemical Research of Kaunas University of Medicine	2007	2007	Lithuania	*Information not reported*	*1000*	*19*	*64*	*Yes*	*Yes*	*Single 24-hour recall*	*Nationally representative*
GDD 2017	Health Behavior in School-Aged Children 2006	2006	2006	Luxembourg	*Both urban and rural*	*4265*	*11*	*15*	*Yes*	*Yes*	*FFQ*	*Nationally representative*
GDD 2017	Health Behavior in School-Aged Children 2010	2010	2010	Luxembourg	*Both urban and rural*	*4045*	*11*	*15*	*Yes*	*Yes*	*FFQ*	*Nationally representative*
GDD 2010	Luxembourgian Community Health and Nutrition Survey	1993	1993	Luxembourg	*Information not reported*	*1000*	*0*	*100*	*Yes*	*Yes*	*Household availability/ budget survey*	*Nationally representative*
GDD 2017	Global Student Based Student Health Survey 2007	2007	2007	Macedonia	*Both urban and rural*	*2077*	*7*	*16*	*Yes*	*Yes*	*FFQ*	*Nationally representative*
GDD 2017	Health Behavior in School-Aged Children 2002	2001	2002	Macedonia	*Both urban and rural*	*4030*	*11*	*16*	*Yes*	*Yes*	*FFQ*	*Nationally representative*
GDD 2017	Health Behavior in School-Aged Children 2006	2006	2006	Macedonia	*Both urban and rural*	*5255*	*11*	*15*	*Yes*	*Yes*	*FFQ*	*Nationally representative*
GDD 2017	Health Behavior in School-Aged Children 2010	2009	2010	Macedonia	*Both urban and rural*	*3805*	*11*	*15*	*Yes*	*Yes*	*FFQ*	*Nationally representative*
GDD 2017	Demographic and Health Survey Madagascar 1997	1997	1997	Madagascar	*Both urban and rural*	*2864*	*0*	*2*	*Yes*	*Yes*	*DHS questionnair e*	*Nationally representative*
GDD 2017	Demographic and Health Survey Madagascar 2011	2011	2011	Madagascar	*Both urban and rural*	*5257*	*0*	*4*	*Yes*	*Yes*	*Biomarker*	*Nationally representative*

GDD 2017	Demographic and Health Survey Madagascar 2013	2013	2013	Madagascar	*Both urban and rural*	*4539*	*0*	*4*	*Yes*	*Yes*	*Biomarker*	*Nationally representative*
GDD 2017	Demographic and Health Survey Madagascar 2003	2003	2004	Madagascar	*Both urban and rural*	*6922*	*0*	*49*	*Yes*	*Yes*	*DHS questionnaire*	*Nationally representative*
GDD 2017	Demographic and Health Survey Madagascar 2008	2008	2009	Madagascar	*Both urban and rural*	*28452*	*0*	*59*	*Yes*	*Yes*	*DHS questionnaire*	*Nationally representative*
GDD 2017	Demographic and Health Survey Madagascar 2016	2016	2016	Madagascar	*Both urban and rural*	*5925*	*0*	*4*	*Yes*	*Yes*	*DHS questionnaire*	*Nationally representative*
GDD 2010	Enqu�te sur les facteurs de risque des maladies non transmissibles � Madagascar	2005	2005	Madagascar	*Information not reported*	*1000*	*25*	*64*	*Yes*	*Yes*	*FFQ*	*Sub-nationally representative*
GDD 2017	Global Student Based Student Health Survey 2009	2009	2009	Malawi	*Both urban and rural*	*2243*	*11*	*16*	*Yes*	*Yes*	*FFQ*	*Nationally representative*
GDD 2017	World Health Survey 2003	2003	2003	Malawi	*Both urban and rural*	*4905*	*18*	*96*	*Yes*	*Yes*	*FFQ*	*Nationally representative*
GDD 2017	Demographic and Health Survey Malawi 2012	2012	2012	Malawi	*Both urban and rural*	*1900*	*0*	*4*	*Yes*	*Yes*	*Biomarker*	*Nationally representative*
GDD 2017	Demographic and Health Survey Malawi 2004	2004	2005	Malawi	*Both urban and rural*	*11531*	*0*	*49*	*Yes*	*Yes*	*DHS questionnaire*	*Nationally representative*
GDD 2017	Demographic and Health Survey Malawi 2010	2010	2010	Malawi	*Both urban and rural*	*22343*	*0*	*49*	*Yes*	*Yes*	*DHS questionnaire*	*Nationally representative*
GDD 2017	Demographic and Health Survey Malawi 2014	2014	2014	Malawi	*Both urban and rural*	*1752*	*0*	*4*	*Yes*	*Yes*	*Biomarker*	*Nationally representative*
GDD 2017	Demographic and Health Survey Malawi 2000	2000	2000	Malawi	*Both urban and rural*	*8904*	*0*	*4*	*Yes*	*Yes*	*DHS questionnaire*	*Nationally representative*
GDD 2017	Demographic and Health Survey Malawi 2015	2015	2016	Malawi	*Both urban and rural*	*19341*	*0*	*49*	*Yes*	*Yes*	*DHS questionnaire*	*Nationally representative*
GDD 2010	Simmons et al. (1 of 2)	1983	1983	Malawi	*Information not reported*	*78*	*20*	*79*	*Yes*	*Yes*	*Biomarker*	*Locally representative*
GDD 2010	Simmons et al. (2 of 2)	1983	1983	Malawi	*Information not reported*	*123*	*20*	*79*	*Yes*	*Yes*	*Biomarker*	*Locally representative*

GDD 2017	Malaysia Lipid Study	2012	2013	Malaysia	*Only urban*	*577*	*20*	*67*	*Yes*	*Yes*	*Multiple 24-hour recall*	*Locally representative*
GDD 2017	SEANUTS: South East Asian Nutrition Surveys	2010	2011	Malaysia	*Both urban and rural*	*3542*	*0*	*12*	*Yes*	*Yes*	*FFQ*	*Nationally representative*
GDD 2017	Seremban Cohort Study (SeCoSt)	2012	2017	Malaysia	*Only urban*	*294*	*18*	*43*	*No*	*Yes*	*Multiple 24-hour recall*	*Locally representative*
GDD 2017	The Universiti Sains Malaysia Pregnancy Cohort Study	2010	2015	Malaysia	*Only rural*	*153*	*19*	*41*	*No*	*Yes*	*FFQ*	*Locally representative*
GDD 2017	Clustering of lifestyle risk factors and understanding its association with stress on health and wellbeing among school teachers in Malaysia (CLUSTer)	2014	2015	Malaysia	*Both urban and rural*	*4801*	*19*	*70*	*Yes*	*Yes*	*FFQ*	*Locally representative*
GDD 2017	Dietary intake of second and third trimester pregnant women in East Coast of Peninsular Malaysia	2014	2015	Malaysia	*Only urban*	*601*	*17*	*46*	*No*	*Yes*	*Single 24-hour recall*	*Sub-nationally representative*
GDD 2017	PREDICTORS OF POSTPARTUM WEIGHT RETENTION AMONG URBAN MALAYSIAN MOTHERS: A PROSPECTIVE COHORT STUDY	2013	2014	Malaysia	*Only urban*	*420*	*19*	*43*	*No*	*Yes*	*FFQ*	*Locally representative*
GDD 2017	Antenatal calcium intake in Malaysia	2006	2006	Malaysia	*Only urban*	*150*	*16*	*44*	*No*	*Yes*	*Multiple 24-hour recall*	*Locally representative*
GDD 2017	Global Student Based Student Health Survey 2012	2012	2012	Malaysia	*Both urban and rural*	*25448*	*7*	*16*	*Yes*	*Yes*	*FFQ*	*Nationally representative*
GDD 2017	World Health Survey 2003	2003	2003	Malaysia	*Both urban and rural*	*5638*	*18*	*97*	*Yes*	*Yes*	*FFQ*	*Nationally representative*
GDD 2010	Malaysian Adult Nutrition Survey 2003	2002	2003	Malaysia	*Information not reported*	*1000*	*18*	*59*	*Yes*	*Yes*	*Single 24-hour recall*	*Nationally representative*
GDD 2010	Malaysian Adults Nutrition Survey	2003	2003	Malaysia	*Information not reported*	*866*	*20*	*59*	*Yes*	*Yes*	*FFQ*	*Nationally representative*
GDD 2017	Global Student Based Student Health Survey 2009	2009	2009	Maldives	*Both urban and rural*	*3134*	*11*	*16*	*Yes*	*Yes*	*FFQ*	*Nationally representative*
GDD 2017	Demographic and Health Survey Maldives 2009	2009	2009	Maldives	*Both urban and rural*	*5037*	*0*	*48*	*Yes*	*Yes*	*DHS questionnair e*	*Nationally representative*

GDD 2017	World Health Survey 2003	2003	2003	Mali	*Both urban and rural*	*1738*	*15*	*98*	*Yes*	*Yes*	*FFQ*	*Nationally representative*
GDD 2017	Demographic and Health Survey Mali 2010	2010	2010	Mali	*Both urban and rural*	*1760*	*1*	*5*	*Yes*	*Yes*	*Biomarker*	*Nationally representative*
GDD 2017	Demographic and Health Survey Mali 2012	2012	2013	Mali	*Both urban and rural*	*15613*	*0*	*49*	*Yes*	*Yes*	*DHS questionnaire*	*Nationally representative*
GDD 2017	Demographic and Health Survey Mali 2001	2001	2001	Mali	*Both urban and rural*	*13561*	*0*	*49*	*Yes*	*Yes*	*DHS questionnaire*	*Nationally representative*
GDD 2017	Demographic and Health Survey Mali 2006	2006	2006	Mali	*Both urban and rural*	*11961*	*0*	*49*	*Yes*	*Yes*	*DHS questionnaire*	*Nationally representative*
GDD 2010	Dietary Diversity as a Measure of the Micronutrient Adequacy of Women's Diets: Results from Bamako, Mali Site	2007	2007	Mali	*Information not reported*	*100*	*15*	*49*	*No*	*Yes*	*Multiple 24-hour recall*	*Sub-nationally representative*
GDD 2017	MGSD Gestational Diabetes in the Mediterranean Region	2010	2013	Malta	*Only rural*	*124*	*17*	*41*	*No*	*Yes*	*FFQ*	*Locally representative*
GDD 2017	Health Behavior in School-Aged Children 2002	2001	2002	Malta	*Both urban and rural*	*1945*	*11*	*17*	*Yes*	*Yes*	*FFQ*	*Nationally representative*
GDD 2017	Health Behavior in School-Aged Children 2006	2006	2006	Malta	*Both urban and rural*	*1385*	*11*	*15*	*Yes*	*Yes*	*FFQ*	*Nationally representative*
GDD 2010	Maltese Community Health and Nutrition Survey	1994	1994	Malta	*Information not reported*	*1000*	*0*	*100*	*Yes*	*Yes*	*Household availability/ budget survey*	*Nationally representative*
GDD 2010	Maltese Community Health and Nutrition Survey	1995	1995	Malta	*Information not reported*	*1000*	*0*	*100*	*Yes*	*Yes*	*Household availability/ budget survey*	*Nationally representative*
GDD 2010	Maltese Community Health and Nutrition Survey	2000	2000	Malta	*Information not reported*	*1000*	*0*	*100*	*Yes*	*Yes*	*Household availability/ budget survey*	*Nationally representative*
GDD 2010	2nd European Health Interview Survey	2008	2008	Malta	*Information not reported*	*1000*	*15*	*96*	*Yes*	*Yes*	*Single 24-hour recall*	*Nationally representative*
GDD 2010	INTERSALT, Dingli village	1985	1987	Malta	*Information not reported*	*25*	*20*	*59*	*Yes*	*Yes*	*Biomarker*	*Locally representative*

GDD 2010	WHO STEPwise approach to Surveillance of Non-Communicable Diseases (STEPS) Republic of the Marshall Islands Report DRAFT Version 2.5 May 2007	2002	2002	Marshall Islands	*Information not reported*	*1000*	*15*	*64*	*Yes*	*Yes*	*FFQ*	*Nationally representative*
GDD 2017	Global Student Based Student Health Survey 2010	2010	2010	Mauritania	*Both urban and rural*	*1997*	*11*	*16*	*Yes*	*Yes*	*FFQ*	*Nationally representative*
GDD 2017	World Health Survey 2003	2003	2003	Mauritania	*Both urban and rural*	*2782*	*15*	*90*	*Yes*	*Yes*	*FFQ*	*Nationally representative*
GDD 2010	STEP Mauritania	2006	2006	Mauritania	*Information not reported*	*1000*	*15*	*64*	*Yes*	*Yes*	*FFQ*	*Sub-nationally representative*
GDD 2017	FOOD HABITS AND ENERGY INTAKE AMONG THE MAURITIAN POPULATION	2014	2015	Mauritius	*Both urban and rural*	*897*	*6*	*55*	*Yes*	*Yes*	*FFQ*	*Nationally representative*
GDD 2017	Dietary Habits and energy Intake- Mauritius population	2013	2014	Mauritius	*Both urban and rural*	*1009*	*11*	*65*	*Yes*	*Yes*	*FFQ*	*Sub-nationally representative*
GDD 2017	Global Student Based Student Health Survey 2011	2011	2011	Mauritius	*Both urban and rural*	*2161*	*11*	*16*	*Yes*	*Yes*	*FFQ*	*Nationally representative*
GDD 2017	World Health Survey 2003	2003	2003	Mauritius	*Both urban and rural*	*3158*	*18*	*93*	*Yes*	*Yes*	*FFQ*	*Nationally representative*
GDD 2017	Study of Global Ageing and Adult Health	2007	2013	Mexico	*Both urban and rural*	*2592*	*22*	*101*	*Yes*	*Yes*	*FFQ*	*Nationally representative*
GDD 2017	Demographic and Health Survey Mexico 1987	1987	1987	Mexico	*Both urban and rural*	*876*	*0*	*4*	*Yes*	*Yes*	*DHS questionnair e*	*Nationally representative*
GDD 2017	ENSANUT	2006	2006	Mexico	*Both urban and rural*	*24271*	*12*	*59*	*Yes*	*Yes*	*FFQ*	*Nationally representative*
GDD 2010	National Health and Nutrition Survey 2006 (Mexico)	2005	2006	Mexico	*Information not reported*	*1000*	*20*	*59*	*Yes*	*Yes*	*FFQ*	*Nationally representative*
GDD 2010	INTERSALT, Chihuahua	1985	1987	Mexico	*Information not reported*	*23*	*20*	*59*	*Yes*	*Yes*	*Biomarker*	*Sub-nationally representative*
GDD 2010	Federal States of Micronesia (Pohnpei) NCD Risk Factors STEPS Report	2002	2002	Micronesia, Federated States Of	*Information not reported*	*1000*	*25*	*64*	*Yes*	*Yes*	*FFQ*	*Sub-nationally representative*
GDD 2010	Living Conditions Lifestyles and Health	2001	2002	Moldova	*Information not reported*	*1000*	*18*	*99*	*Yes*	*Yes*	*FFQ*	*Nationally representative*
GDD 2017	Global Student Based Student Health Survey 2013	2013	2013	Mongolia	*Both urban and rural*	*5355*	*7*	*16*	*Yes*	*Yes*	*FFQ*	*Nationally representative*

GDD 2010	Mongolian STEPS survey on the prevalence of noncommunicable disease risk factors 2006	2005	2005	Mongolia	*Information not reported*	*1000*	*15*	*64*	*Yes*	*Yes*	*FFQ*	*Nationally representative*
GDD 2010	STEP Mongolia	2009	2009	Mongolia	*Information not reported*	*1000*	*15*	*64*	*Yes*	*Yes*	*FFQ*	*Nationally representative*
GDD 2010	Yamada et al.	1998	1998	Mongolia	*Information not reported*	*193*	*20*	*44*	*Yes*	*Yes*	*Biomarker*	*Nationally representative*
GDD 2010	Montenegrin Community Health and Nutrition Survey	2003	2003	Montenegro	*Information not reported*	*1000*	*0*	*100*	*Yes*	*Yes*	*Household availability/ budget survey*	*Nationally representative*
GDD 2010	Montenegrin Community Health and Nutrition Survey	2004	2004	Montenegro	*Information not reported*	*1000*	*0*	*100*	*Yes*	*Yes*	*Household availability/ budget survey*	*Nationally representative*
GDD 2017	Combined consumption of a single high-dose vitamin A supplement and provision of vitamin A fortified oil to households maintains adequate milk retinol concentrations for 6 months in lactating Moroccan women	2009	2010	Morocco	*Only urban*	*92*	*15*	*45*	*No*	*Yes*	*FFQ*	*Locally representative*
GDD 2017	Arab Teens Lifestyle Study (ATLS)	2011	2012	Morocco	*Only urban*	*558*	*15*	*25*	*Yes*	*Yes*	*FFQ*	*Locally representative*
GDD 2017	Inadequacy of vitamins and minerals among high-school pupils in Ouarzazate, Morocco	2007	2008	Morocco	*Both urban and rural*	*346*	*15*	*27*	*Yes*	*Yes*	*Multiple 24-hour recall*	*Locally representative*
GDD 2017	Global Student Based Student Health Survey 2006	2006	2006	Morocco	*Both urban and rural*	*2527*	*11*	*16*	*Yes*	*Yes*	*FFQ*	*Nationally representative*
GDD 2017	World Health Survey 2003	2003	2003	Morocco	*Both urban and rural*	*2073*	*18*	*98*	*Yes*	*No*	*FFQ*	*Nationally representative*
GDD 2017	Global Student Based Student Health Survey 2010	2010	2010	Morocco	*Both urban and rural*	*2852*	*11*	*16*	*Yes*	*Yes*	*FFQ*	*Nationally representative*
GDD 2017	STEPS	2005	2005	Mozambique	*Both urban and rural*	*3289*	*25*	*64*	*Yes*	*Yes*	*DHS questionnair e*	*Nationally representative*
GDD 2017	Food Consumption and Culinary Practices in Maputo, Mozambique	2012	2013	Mozambique	*Only urban*	*100*	*25*	*62*	*Yes*	*Yes*	*Single 24-hour recall*	*Locally representative*
GDD 2017	Demographic and Health Suvey Mozambique 2011	2011	2011	Mozambique	*Both urban and rural*	*22864*	*0*	*49*	*Yes*	*Yes*	*DHS questionnair e*	*Nationally representative*

GDD 2010	Dietary Diversity as a Measure of the Micronutrient Adequacy of Women's Diets: Results from Rural Mozambique Site	2006	2006	Mozambique	*Information not reported*	*250*	*15*	*49*	*No*	*Yes*	*Single 24-hour recall*	*Sub-nationally representative*
GDD 2017	Global Student Based Student Health Survey 2007	2007	2007	Myanmar	*Both urban and rural*	*2784*	*11*	*16*	*Yes*	*Yes*	*FFQ*	*Nationally representative*
GDD 2017	World Health Survey 2003	2003	2003	Myanmar	*Both urban and rural*	*5886*	*18*	*99*	*Yes*	*Yes*	*FFQ*	*Nationally representative*
GDD 2017	World Health Survey 2003	2003	2003	Namibia	*Both urban and rural*	*3610*	*18*	*103*	*Yes*	*Yes*	*FFQ*	*Nationally representative*
GDD 2017	Demographic and Health Survey Namibia 2006	2006	2007	Namibia	*Both urban and rural*	*5833*	*0*	*49*	*Yes*	*Yes*	*DHS questionnair e*	*Nationally representative*
GDD 2017	Demographic and Health Survey Namibia 2013	2013	2013	Namibia	*Both urban and rural*	*17590*	*0*	*64*	*Yes*	*Yes*	*DHS questionnair e*	*Nationally representative*
GDD 2017	Global Student Based Student Health Survey 2013	2013	2013	Namibia	*Both urban and rural*	*4429*	*7*	*16*	*Yes*	*Yes*	*FFQ*	*Nationally representative*
GDD 2017	Global Student Based Student Health Survey 2011	2011	2011	Nauru	*Only urban*	*532*	*11*	*16*	*Yes*	*Yes*	*FFQ*	*Nationally representative*
GDD 2010	Nauru NCD risk factors STEPS report	2004	2004	Nauru	*Information not reported*	*1000*	*15*	*64*	*Yes*	*Yes*	*FFQ*	*Nationally representative*
GDD 2017	Bhaktapur Female Dietary Survey	2001	2001	Nepal	*Both urban and rural*	*379*	*15*	*35*	*No*	*Yes*	*FFQ*	*Locally representative*
GDD 2017	Micronutrient status in breastfeeding women and their babies in Bhaktapur Nepal -	2008	2009	Nepal	*Both urban and rural*	*466*	*17*	*44*	*No*	*Yes*	*Multiple 24-hour recall*	*Sub-nationally representative*
GDD 2017	Demographic and Health Survey Nepal 2001	2001	2001	Nepal	*Both urban and rural*	*5170*	*0*	*4*	*Yes*	*Yes*	*DHS questionnair e*	*Nationally representative*
GDD 2017	Demographic and Health Survey Nepal 2006	2006	2006	Nepal	*Both urban and rural*	*16077*	*0*	*49*	*Yes*	*Yes*	*DHS questionnair e*	*Nationally representative*
GDD 2017	Demographic and Health Survey Nepal 2011	2011	2011	Nepal	*Both urban and rural*	*9917*	*0*	*49*	*Yes*	*Yes*	*DHS questionnair e*	*Nationally representative*
GDD 2017	Nepal World Health Survey 2003	2003	2003	Nepal	*Both urban and rural*	*8678*	*15*	*102*	*Yes*	*Yes*	*FFQ*	*Nationally representative*

Miller V, *et al. BMJ Global Health* 2021; 6:e003585. doi: 10.1136/bmjgh-2020-003585

GDD 2010	Food consumption patterns and nutrient intake among Nepalese living in the southern rural Terai region	1989	1989	Nepal	*Information not reported*	*100*	*10*	*68*	*Yes*	*Yes*	*Single 24-hour recall*	*Sub-nationally representative*
GDD 2017	Diet of community-dwelling older adults 2010-2012	2010	2012	Netherlands	*Both urban and rural*	*1478*	*70*	*94*	*Yes*	*Yes*	*Multiple 24-hour recall*	*Nationally representative*
GDD 2017	Dutch National Food Consumption Survey 2007-2010 Diet of Children and adults aged 7-69 years	2007	2010	Netherlands	*Both urban and rural*	*7630*	*7*	*69*	*Yes*	*Yes*	*Multiple 24-hour recall*	*Nationally representative*
GDD 2017	Health Behavior in School-Aged Children 2002	2001	2002	Netherlands	*Both urban and rural*	*4268*	*11*	*16*	*Yes*	*Yes*	*FFQ*	*Nationally representative*
GDD 2017	Health Behavior in School-Aged Children 2006	2006	2006	Netherlands	*Both urban and rural*	*4217*	*11*	*15*	*Yes*	*Yes*	*FFQ*	*Nationally representative*
GDD 2017	Health Behavior in School-Aged Children 2010	2010	2010	Netherlands	*Both urban and rural*	*4506*	*11*	*15*	*Yes*	*Yes*	*FFQ*	*Nationally representative*
GDD 2010	Dutch National Food Consumption Survey	1987	1988	Netherlands	*Information not reported*	*1000*	*20*	*85*	*Yes*	*Yes*	*Multiple 24-hour recall*	*Nationally representative*
GDD 2010	Dutch National Food Consumption Survey	1992	1992	Netherlands	*Information not reported*	*1000*	*20*	*92*	*Yes*	*Yes*	*Multiple 24-hour recall*	*Nationally representative*
GDD 2010	Dutch National Food Consumption Survey	1997	1998	Netherlands	*Information not reported*	*1000*	*20*	*97*	*Yes*	*Yes*	*Multiple 24-hour recall*	*Nationally representative*
GDD 2010	Dutch National Food Consumption Survey	1997	1998	Netherlands	*Information not reported*	*1000*	*15*	*100*	*Yes*	*Yes*	*Multiple 24-hour recall*	*Nationally representative*
GDD 2010	Dutch National Food Consumption Survey	2003	2003	Netherlands	*Information not reported*	*500*	*20*	*31*	*Yes*	*Yes*	*Multiple 24-hour recall*	*Nationally representative*
GDD 2010	Dutch National Food Consumption Survey - Young Children	2005	2006	Netherlands	*Information not reported*	*1000*	*4*	*14*	*Yes*	*Yes*	*Multiple 24-hour recall*	*Nationally representative*
GDD 2010	Participants of Doetinchem Cohort Study	2006	2006	Netherlands	*Information not reported*	*250*	*19*	*70*	*Yes*	*Yes*	*Single 24-hour recall*	*Locally representative*
GDD 2010	TRANSFAIR study	1992	1992	Netherlands	*Information not reported*	*1000*	*19*	*64*	*Yes*	*Yes*	*Single 24-hour recall*	*Nationally representative*

GDD 2010	INTERSALT, Zutphen	1985	1987	Netherlands	*Information not reported*	*25*	*20*	*59*	*Yes*	*Yes*	*Biomarker*	*Sub-nationally representative*
GDD 2010	Ocke et al.	2006	2006	Netherlands	*Information not reported*	*6*	*20*	*74*	*Yes*	*Yes*	*Biomarker*	*Sub-nationally representative*
GDD 2010	NZ National Nutrition Survey 1997	1997	1997	New Zealand	*Information not reported*	*1000*	*15*	*100*	*Yes*	*Yes*	*Multiple 24-hour recall*	*Nationally representative*
GDD 2010	NZ Childrens Nutrition Survey	2002	2002	New Zealand	*Information not reported*	*1000*	*5*	*14*	*Yes*	*Yes*	*Multiple 24-hour recall*	*Nationally representative*
GDD 2010	Simpson et al.	1981	1981	New Zealand	*Information not reported*	*113*	*20*	*93*	*Yes*	*Yes*	*Biomarker*	*Sub-nationally representative*
GDD 2010	Thomson and Colls	1993	1998	New Zealand	*Information not reported*	*55*	*20*	*69*	*Yes*	*Yes*	*Biomarker*	*Sub-nationally representative*
GDD 2017	Demographic and Health Survey Niger 2006	2006	2006	Niger	*Both urban and rural*	*14154*	*0*	*59*	*Yes*	*Yes*	*DHS questionnaire*	*Nationally representative*
GDD 2017	Demographic and Health Survey Niger 2012	2012	2012	Niger	*Both urban and rural*	*18169*	*0*	*59*	*Yes*	*Yes*	*DHS questionnaire*	*Nationally representative*
GDD 2010	Measurement of NCD Risk Factors in Niger WHO STEP wise Approach	2007	2007	Niger	*Information not reported*	*1000*	*15*	*64*	*Yes*	*Yes*	*FFQ*	*Nationally representative*
GDD 2010	Street foods and dietary intakes of Nigerian urban market women	1995	1995	Niger	*Information not reported*	*100*	*19*	*66*	*No*	*Yes*	*Multiple 24-hour recall*	*Locally representative*
GDD 2017	Demographic and Health Survey Nigeria 2008	2008	2008	Nigeria	*Both urban and rural*	*35175*	*0*	*49*	*Yes*	*Yes*	*DHS questionnaire*	*Nationally representative*
GDD 2017	Demographic and Health Survey Nigeria 2013	2013	2013	Nigeria	*Both urban and rural*	*19153*	*0*	*4*	*Yes*	*Yes*	*DHS questionnaire*	*Nationally representative*
GDD 2017	Demographic and Health Survey Nigeria 2015	2015	2015	Nigeria	*Both urban and rural*	*5298*	*0*	*4*	*Yes*	*Yes*	*Biomarker*	*Nationally representative*
GDD 2010	Cooper et al.	1991	1994	Nigeria	*Information not reported*	*2509*	*45*	*94*	*Yes*	*Yes*	*Biomarker*	*Sub-nationally representative*
GDD 2017	Global Student Based Student Health Survey 2010	2010	2010	Niue	*Both urban and rural*	*141*	*11*	*16*	*Yes*	*Yes*	*FFQ*	*Nationally representative*
GDD 2010	Taylor et al. (1 of 3)	1980	1981	Niue	*Information not reported*	*19*	*20*	*79*	*Yes*	*Yes*	*Biomarker*	*Nationally representative*
GDD 2017	Health Behavior in School-Aged Children 1990	1989	1990	Norway	*Both urban and rural*	*4985*	*10*	*16*	*Yes*	*Yes*	*FFQ*	*Nationally representative*

GDD 2017	Health Behavior in School-Aged Children 1994	1993	1994	Norway	*Both urban and rural*	*4897*	*11*	*16*	*Yes*	*Yes*	*FFQ*	*Nationally representative*
GDD 2017	Health Behavior in School-Aged Children 1998	1998	1998	Norway	*Both urban and rural*	*4983*	*11*	*16*	*Yes*	*Yes*	*FFQ*	*Nationally representative*
GDD 2017	Health Behavior in School-Aged Children 2002	2001	2002	Norway	*Both urban and rural*	*5015*	*11*	*16*	*Yes*	*Yes*	*FFQ*	*Nationally representative*
GDD 2017	Health Behavior in School-Aged Children 2006	2006	2006	Norway	*Both urban and rural*	*4675*	*11*	*15*	*Yes*	*Yes*	*FFQ*	*Nationally representative*
GDD 2017	Health Behavior in School-Aged Children 2010	2010	2010	Norway	*Both urban and rural*	*4307*	*11*	*15*	*Yes*	*Yes*	*FFQ*	*Nationally representative*
GDD 2010	Norwegian Community Health and Nutrition Survey	1986	1988	Norway	*Information not reported*	*1000*	*0*	*100*	*Yes*	*Yes*	*Household availability/ budget survey*	*Nationally representative*
GDD 2010	Norwegian Community Health and Nutrition Survey	1992	1994	Norway	*Information not reported*	*1000*	*0*	*100*	*Yes*	*Yes*	*Household availability/ budget survey*	*Nationally representative*
GDD 2010	Norwegian national dietary survey 1997 (Norkost 1997)	1997	1997	Norway	*Information not reported*	*1000*	*20*	*79*	*Yes*	*Yes*	*FFQ*	*Nationally representative*
GDD 2010	Norwegian national dietary survey 1997 (Norkost 1997)	1997	1997	Norway	*Information not reported*	*1000*	*15*	*18*	*Yes*	*Yes*	*FFQ*	*Nationally representative*
GDD 2010	Norwegian Community Health and Nutrition Survey	1996	1998	Norway	*Information not reported*	*1000*	*0*	*100*	*Yes*	*Yes*	*Household availability/ budget survey*	*Nationally representative*
GDD 2010	Nutritional Status of Norwegian children	2000	2000	Norway	*Information not reported*	*1000*	*4*	*13*	*Yes*	*Yes*	*Multiple 24-hour recall*	*Nationally representative*
GDD 2010	TRANSFAIR study	1993	1994	Norway	*Information not reported*	*1000*	*19*	*64*	*Yes*	*Yes*	*FFQ*	*Nationally representative*
GDD 2017	Global Student Based Student Health Survey 2005	2005	2005	Oman	*Both urban and rural*	*2906*	*11*	*16*	*Yes*	*Yes*	*FFQ*	*Nationally representative*
GDD 2017	Global Student Based Student Health Survey 2010	2010	2010	Oman	*Both urban and rural*	*1532*	*11*	*16*	*Yes*	*Yes*	*FFQ*	*Nationally representative*

GDD 2017	Relation between nutritional status and immune functions in old Pakistanis	2008	2009	Pakistan	*Both urban and rural*	*750*	*18*	*95*	*Yes*	*Yes*	*FFQ*	*Locally representative*
GDD 2017	World Health Survey 2003	2003	2003	Pakistan	*Both urban and rural*	*5931*	*18*	*110*	*Yes*	*Yes*	*FFQ*	*Nationally representative*
GDD 2017	Demographic and Health Survey Pakistan 2012	2012	2013	Pakistan	*Both urban and rural*	*6821*	*0*	*4*	*Yes*	*Yes*	*DHS questionnaire*	*Nationally representative*
GDD 2017	Global Student Based Student Health Survey 2009	2009	2009	Pakistan	*Both urban and rural*	*5177*	*11*	*16*	*Yes*	*Yes*	*FFQ*	*Nationally representative*
GDD 2010	National Health Survey of Pakistan	1991	1994	Pakistan	*Information not reported*	*1000*	*0*	*110*	*Yes*	*Yes*	*FFQ*	*Nationally representative*
GDD 2017	Global Student Based Student Health Survey 2010	2010	2010	Palestinian Territory, Occupied	*Both urban and rural*	*8545*	*11*	*16*	*Yes*	*Yes*	*FFQ*	*Nationally representative*
GDD 2010	WHO STEPS Chronic Disease Risk Factor Surveillance Data Book for Papua New Guinea 2007-2008	2007	2007	Papua New Guinea	*Information not reported*	*1000*	*25*	*64*	*Yes*	*Yes*	*FFQ*	*Nationally representative*
GDD 2017	World Health Survey 2003	2003	2003	Paraguay	*Both urban and rural*	*4801*	*18*	*95*	*Yes*	*Yes*	*FFQ*	*Nationally representative*
GDD 2017	Global Student Based Student Health Survey 2010	2010	2010	Peru	*Both urban and rural*	*2855*	*11*	*16*	*Yes*	*Yes*	*FFQ*	*Nationally representative*
GDD 2017	Demographic and Health Survey Peru 2004	2004	2006	Peru	*Both urban and rural*	*11521*	*0*	*49*	*Yes*	*Yes*	*DHS questionnaire*	*Nationally representative*
GDD 2017	Demographic and Health Survey Peru 2009	2009	2009	Peru	*Both urban and rural*	*7100*	*0*	*4*	*Yes*	*Yes*	*DHS questionnaire*	*Nationally representative*
GDD 2017	Demographic and Health Survey Peru 2007	2007	2008	Peru	*Both urban and rural*	*27897*	*0*	*49*	*Yes*	*Yes*	*DHS questionnaire*	*Nationally representative*
GDD 2017	Demographic and Health Survey Peru 2010	2010	2010	Peru	*Both urban and rural*	*30937*	*0*	*49*	*Yes*	*Yes*	*DHS questionnaire*	*Nationally representative*
GDD 2017	Demographic and Health Survey Peru 2011	2011	2011	Peru	*Both urban and rural*	*30625*	*0*	*49*	*Yes*	*Yes*	*DHS questionnaire*	*Nationally representative*
GDD 2017	Demographic and Health Survey Peru 2012	2012	2012	Peru	*Both urban and rural*	*32728*	*0*	*49*	*Yes*	*Yes*	*DHS questionnaire*	*Nationally representative*

GDD 2017	Demographic and Health Survey Peru 1996	1996	1996	Peru	*Both urban and rural*	*14391*	*0*	*49*	*Yes*	*Yes*	*DHS questionnaire*	*Nationally representative*
GDD 2017	Demographic and Health Survey Peru 2000	2000	2000	Peru	*Both urban and rural*	*16472*	*0*	*49*	*Yes*	*Yes*	*DHS questionnaire*	*Nationally representative*
GDD 2017	Cebu Longitudinal Health and Nutrition Survey	1993	1994	Philippines	*Both urban and rural*	*2181*	*10*	*12*	*Yes*	*Yes*	*Multiple 24-hour recall*	*Locally representative*
GDD 2017	Cebu Longitudinal Health and Nutrition Survey	1997	1998	Philippines	*Both urban and rural*	*4174*	*14*	*16*	*Yes*	*Yes*	*Multiple 24-hour recall*	*Locally representative*
GDD 2017	Cebu Longitudinal Health and Nutrition Survey	2001	2002	Philippines	*Both urban and rural*	*4045*	*17*	*19*	*Yes*	*Yes*	*Multiple 24-hour recall*	*Locally representative*
GDD 2017	Cebu Longitudinal Health and Nutrition Survey	2004	2005	Philippines	*Both urban and rural*	*3773*	*20*	*22*	*Yes*	*Yes*	*Multiple 24-hour recall*	*Locally representative*
GDD 2017	Global Student Based Student Health Survey 2011	2011	2011	Philippines	*Both urban and rural*	*5227*	*11*	*16*	*Yes*	*Yes*	*FFQ*	*Nationally representative*
GDD 2017	Global Student Based Student Health Survey 2003	2003	2003	Philippines	*Both urban and rural*	*7074*	*11*	*16*	*Yes*	*Yes*	*FFQ*	*Nationally representative*
GDD 2017	Global Student Based Student Health Survey 2007	2007	2007	Philippines	*Both urban and rural*	*5583*	*11*	*16*	*Yes*	*Yes*	*FFQ*	*Nationally representative*
GDD 2017	Demographic and Health Survey Philippines 1998	1998	1998	Philippines	*Both urban and rural*	*7603*	*0*	*4*	*Yes*	*Yes*	*DHS questionnaire*	*Nationally representative*
GDD 2017	World Health Survey 2003	2003	2003	Philippines	*Both urban and rural*	*9009*	*18*	*94*	*Yes*	*Yes*	*FFQ*	*Nationally representative*
GDD 2017	Demographic and Health Survey Philippines 2003	2003	2003	Philippines	*Both urban and rural*	*4729*	*0*	*4*	*Yes*	*Yes*	*DHS questionnaire*	*Nationally representative*
GDD 2017	Demographic and Health Survey Philippines 2008	2008	2008	Philippines	*Both urban and rural*	*8204*	*0*	*49*	*Yes*	*Yes*	*DHS questionnaire*	*Nationally representative*
GDD 2010	6TH NATIONAL NUTRITION SURVEY, PHILIPPINES	2003	2003	Philippines	*Information not reported*	*1000*	*20*	*100*	*Yes*	*Yes*	*FFQ*	*Nationally representative*
GDD 2017	Household Food Consumption and Anthropometric Survey	2000	2000	Poland	*Both urban and rural*	*4110*	*2*	*100*	*Yes*	*Yes*	*Single 24-hour recall*	*Nationally representative*

GDD 2017	WOBASZ II	2013	2014	Poland	*Both urban and rural*	*5690*	*18*	*100*	*Yes*	*Yes*	*Single 24-hour recall*	*Nationally representative*
GDD 2017	Health Behavior in School-Aged Children 1990	1989	1990	Poland	*Both urban and rural*	*4588*	*11*	*17*	*Yes*	*Yes*	*FFQ*	*Nationally representative*
GDD 2017	Health Behavior in School-Aged Children 1994	1993	1994	Poland	*Both urban and rural*	*4316*	*11*	*16*	*Yes*	*Yes*	*FFQ*	*Nationally representative*
GDD 2017	Health Behavior in School-Aged Children 1998	1998	1998	Poland	*Both urban and rural*	*4833*	*11*	*16*	*Yes*	*Yes*	*FFQ*	*Nationally representative*
GDD 2017	Health Behavior in School-Aged Children 2002	2001	2002	Poland	*Both urban and rural*	*6310*	*11*	*17*	*Yes*	*Yes*	*FFQ*	*Nationally representative*
GDD 2017	Health Behavior in School-Aged Children 2006	2006	2006	Poland	*Both urban and rural*	*5474*	*11*	*15*	*Yes*	*Yes*	*FFQ*	*Nationally representative*
GDD 2017	Health Behavior in School-Aged Children 2010	2010	2010	Poland	*Both urban and rural*	*4223*	*11*	*15*	*Yes*	*Yes*	*FFQ*	*Nationally representative*
GDD 2010	Polish Community Health and Nutrition Survey	1988	1988	Poland	*Information not reported*	*1000*	*0*	*100*	*Yes*	*Yes*	*Household availability/ budget survey*	*Nationally representative*
GDD 2010	Pol-MONICA Warsaw Project � part of WHO MONICA Project (MONitoring of Trends and Determinants of CArdiovascular Diseases)	1993	1993	Poland	*Information not reported*	*1000*	*35*	*64*	*Yes*	*Yes*	*Single 24-hour recall*	*Sub-nationally representative*
GDD 2010	Household Food Consumption and Anthropometric Survey	2000	2000	Poland	*Information not reported*	*1000*	*20*	*100*	*Yes*	*Yes*	*Single 24-hour recall*	*Nationally representative*
GDD 2010	Household Food Consumption and Anthropometric Survey	2000	2000	Poland	*Information not reported*	*1000*	*4*	*100*	*Yes*	*Yes*	*Single 24-hour recall*	*Nationally representative*
GDD 2010	Pol-MONICA bis Warsaw Project	2001	2001	Poland	*Information not reported*	*1000*	*20*	*74*	*Yes*	*Yes*	*Single 24-hour recall*	*Sub-nationally representative*
GDD 2010	WOBASZ - National Multicenter Health Survey	2003	2005	Poland	*Information not reported*	*1000*	*20*	*74*	*Yes*	*Yes*	*Single 24-hour recall*	*Nationally representative*
GDD 2010	INTERSALT, Warsaw	1985	1987	Poland	*Information not reported*	*25*	*20*	*59*	*Yes*	*Yes*	*Biomarker*	*Sub-nationally representative*

GDD 2010	INTERSALT, Krakow	1985	1987	Poland	*Information not reported*	*25*	*20*	*59*	*Yes*	*Yes*	*Biomarker*	*Sub-nationally representative*
GDD 2017	Health Behavior in School-Aged Children 1998	1998	1998	Portugal	*Both urban and rural*	*3632*	*11*	*17*	*Yes*	*Yes*	*FFQ*	*Nationally representative*
GDD 2017	Health Behavior in School-Aged Children 2002	2001	2002	Portugal	*Both urban and rural*	*2928*	*11*	*17*	*Yes*	*Yes*	*FFQ*	*Nationally representative*
GDD 2017	Health Behavior in School-Aged Children 2006	2006	2006	Portugal	*Both urban and rural*	*3895*	*11*	*15*	*Yes*	*Yes*	*FFQ*	*Nationally representative*
GDD 2017	Health Behavior in School-Aged Children 2010	2010	2010	Portugal	*Both urban and rural*	*4020*	*11*	*15*	*Yes*	*Yes*	*FFQ*	*Nationally representative*
GDD 2010	Portuguese Community Health and Nutrition Survey	1990	1990	Portugal	*Information not reported*	*1000*	*0*	*100*	*Yes*	*Yes*	*Household availability/ budget survey*	*Nationally representative*
GDD 2010	Portuguese Community Health and Nutrition Survey	1995	1995	Portugal	*Information not reported*	*1000*	*0*	*100*	*Yes*	*Yes*	*Household availability/ budget survey*	*Nationally representative*
GDD 2010	Portuguese Community Health and Nutrition Survey	2000	2000	Portugal	*Information not reported*	*1000*	*0*	*100*	*Yes*	*Yes*	*Household availability/ budget survey*	*Nationally representative*
GDD 2010	Community-based survey of children from primary schools of Portugal	2002	2003	Portugal	*Information not reported*	*1000*	*7*	*9*	*Yes*	*Yes*	*Single 24-hour recall*	*Nationally representative*
GDD 2010	EPITeen Project	2003	2003	Portugal	*Information not reported*	*1000*	*13*	*100*	*Yes*	*Yes*	*Single 24-hour recall*	*Nationally representative*
GDD 2010	TRANSFAIR study	1988	1989	Portugal	*Information not reported*	*50*	*38*	*38*	*Yes*	*No*	*Single 24-hour recall*	*Nationally representative*
GDD 2010	INTERSALT, Cartaxo Village	1985	1987	Portugal	*Information not reported*	*25*	*20*	*59*	*Yes*	*Yes*	*Biomarker*	*Sub-nationally representative*
GDD 2010	Polonia	2000	2000	Portugal	*Information not reported*	*426*	*20*	*84*	*Yes*	*Yes*	*Biomarker*	*Sub-nationally representative*
GDD 2017	Oral Health Status of 12-year olds residing in PR	2010	2011	Puerto Rico	*Both urban and rural*	*785*	*11*	*13*	*Yes*	*Yes*	*Single 24-hour recall*	*Nationally representative*

GDD 2017	Validation and reproducibility of a semi-quantitative food frequency questionnaire for use in Puerto Riican Children	2002	2003	Puerto Rico	*Only urban*	*554*	*8*	*18*	*Yes*	*Yes*	*Multiple 24-hour recall*	*Locally representative*
GDD 2017	Global Student Based Student Health Survey 2011	2011	2011	Qatar	*Both urban and rural*	*1967*	*11*	*16*	*Yes*	*Yes*	*FFQ*	*Nationally representative*
GDD 2017	Health Behavior in School-Aged Children 2006	2006	2006	Romania	*Both urban and rural*	*4648*	*11*	*15*	*Yes*	*Yes*	*FFQ*	*Nationally representative*
GDD 2017	Health Behavior in School-Aged Children 2010	2010	2010	Romania	*Both urban and rural*	*5322*	*11*	*15*	*Yes*	*Yes*	*FFQ*	*Nationally representative*
GDD 2010	National synthesis, 2006	2006	2006	Romania	*Information not reported*	*1000*	*19*	*100*	*Yes*	*Yes*	*FFQ*	*Nationally representative*
GDD 2017	Study of Global Ageing and Adult Health	2007	2013	Russian Federation	*Both urban and rural*	*3737*	*18*	*100*	*Yes*	*Yes*	*FFQ*	*Nationally representative*
GDD 2017	World Health Survey 2003	2003	2003	Russian Federation	*Both urban and rural*	*1837*	*18*	*94*	*Yes*	*Yes*	*FFQ*	*Nationally representative*
GDD 2017	Health Behavior in School-Aged Children 1994	1993	1994	Russian Federation	*Both urban and rural*	*4001*	*10*	*16*	*Yes*	*Yes*	*FFQ*	*Nationally representative*
GDD 2017	Health Behavior in School-Aged Children 1998	1998	1998	Russian Federation	*Both urban and rural*	*3991*	*11*	*17*	*Yes*	*Yes*	*FFQ*	*Nationally representative*
GDD 2017	Health Behavior in School-Aged Children 2002	2001	2002	Russian Federation	*Both urban and rural*	*8032*	*11*	*17*	*Yes*	*Yes*	*FFQ*	*Nationally representative*
GDD 2017	Health Behavior in School-Aged Children 2006	2006	2006	Russian Federation	*Both urban and rural*	*8176*	*11*	*15*	*Yes*	*Yes*	*FFQ*	*Nationally representative*
GDD 2017	Health Behavior in School-Aged Children 2010	2010	2010	Russian Federation	*Both urban and rural*	*5133*	*11*	*15*	*Yes*	*Yes*	*FFQ*	*Nationally representative*
GDD 2010	Russia Longitudinal Monitoring Study- Round 5-1994	1994	1994	Russian Federation	*Information not reported*	*11258*	*0*	*101*	*Yes*	*Yes*	*Household availability/ budget survey*	*Nationally representative*
GDD 2010	Russia Longitudinal Monitoring Study- Round 6-1995	1995	1995	Russian Federation	*Information not reported*	*10291*	*0*	*101*	*Yes*	*Yes*	*Household availability/ budget survey*	*Nationally representative*

GDD 2010	Russia Longitudinal Monitoring Study- Round 7-1996	1996	1996	Russian Federation	*Information not reported*	*10223*	*0*	*97*	*Yes*	*Yes*	*Household availability/ budget survey*	*Nationally representative*
GDD 2010	Russia Longitudinal Monitoring Study- Round 8-(1998-1999)	1998	1999	Russian Federation	*Information not reported*	*10643*	*0*	*98*	*Yes*	*Yes*	*Household availability/ budget survey*	*Nationally representative*
GDD 2010	Russia Longitudinal Monitoring Study- Round 9-2000	2000	2000	Russian Federation	*Information not reported*	*10966*	*0*	*101*	*Yes*	*Yes*	*Household availability/ budget survey*	*Nationally representative*
GDD 2010	Living Conditions Lifestyles and Health	2001	2002	Russian Federation	*Information not reported*	*1000*	*18*	*99*	*Yes*	*Yes*	*FFQ*	*Nationally representative*
GDD 2010	Russia Longitudinal Monitoring Study- Round 10-2001	2001	2001	Russian Federation	*Information not reported*	*12099*	*0*	*102*	*Yes*	*Yes*	*Household availability/ budget survey*	*Nationally representative*
GDD 2010	Russia Longitudinal Monitoring Study- Round 11-2002	2002	2002	Russian Federation	*Information not reported*	*12484*	*0*	*103*	*Yes*	*Yes*	*Household availability/ budget survey*	*Nationally representative*
GDD 2010	Russia Longitudinal Monitoring Study- Round 12-2003	2003	2003	Russian Federation	*Information not reported*	*12628*	*0*	*101*	*Yes*	*Yes*	*Household availability/ budget survey*	*Nationally representative*
GDD 2010	Russia Longitudinal Monitoring Study- Round 13-2004	2004	2004	Russian Federation	*Information not reported*	*12632*	*0*	*102*	*Yes*	*Yes*	*Household availability/ budget survey*	*Nationally representative*
GDD 2010	Russia Longitudinal Monitoring Study- Round 14-2005	2005	2005	Russian Federation	*Information not reported*	*12217*	*0*	*103*	*Yes*	*Yes*	*Household availability/ budget survey*	*Nationally representative*
GDD 2010	Russia Longitudinal Monitoring Study- Round 15-2006	2006	2006	Russian Federation	*Information not reported*	*14690*	*0*	*100*	*Yes*	*Yes*	*Household availability/ budget survey*	*Nationally representative*
GDD 2010	Russia Longitudinal Monitoring Study- Round 16-2007	2007	2007	Russian Federation	*Information not reported*	*14518*	*0*	*97*	*Yes*	*Yes*	*Household availability/ budget survey*	*Nationally representative*
GDD 2010	Russia Longitudinal Monitoring Study- Round 17-2008	2008	2008	Russian Federation	*Information not reported*	*14028*	*0*	*96*	*Yes*	*Yes*	*Household availability/*	*Nationally representative*

											budget survey	
GDD 2010	INTERSALT, Moscow	1985	1987	Russian Federation	Information not reported	24	20	59	Yes	Yes	Biomarker	Sub-nationally representative
GDD 2017	Demographic and Health Survey Rwanda 2007	2007	2008	Rwanda	Both urban and rural	11942	0	49	Yes	Yes	Biomarker	Nationally representative
GDD 2017	Demographic and Health Survey Rwanda 2010	2010	2011	Rwanda	Both urban and rural	13514	0	49	Yes	Yes	DHS questionnair e	Nationally representative
GDD 2017	Demographic and Health Survey Rwanda 2014	2014	2015	Rwanda	Both urban and rural	12628	0	49	Yes	Yes	DHS questionnair e	Nationally representative
GDD 2017	Demographic and Health Survey Rwanda 2005	2005	2005	Rwanda	Both urban and rural	17735	0	95	Yes	Yes	DHS questionnair e	Nationally representative
GDD 2017	Global Student Based Student Health Survey 2011	2011	2011	Saint Kitts and Nevis	Both urban and rural	1717	11	16	Yes	Yes	FFQ	Nationally representative
GDD 2010	Saint Kitts Chronic Disease Risk Factor Survey 2008	2007	2007	Saint Kitts and Nevis	Information not reported	1000	25	64	Yes	Yes	FFQ	Sub-nationally representative
GDD 2017	Global Student Based Student Health Survey 2007	2007	2007	Saint Lucia	Both urban and rural	1264	11	16	Yes	Yes	FFQ	Nationally representative
GDD 2010	Cooper et al.	1991	1994	Saint Lucia	Information not reported	1089	25	74	Yes	Yes	Biomarker	Sub-nationally representative
GDD 2017	Global Student Based Student Health Survey 2007	2007	2007	Saint Vincent And Grenadines	Both urban and rural	1296	11	16	Yes	Yes	FFQ	Nationally representative
GDD 2017	Global Student Based Student Health Survey 2011	2011	2011	Samoa	Both urban and rural	2340	11	16	Yes	Yes	FFQ	Nationally representative
GDD 2010	Western Samoa 1990 24 hr recall diet estimates	1991	1991	Samoa	Information not reported	250	25	55	Yes	Yes	Single 24-hour recall	Nationally representative
GDD 2010	Samoa NCD Risk Factors STEPS Report	2002	2002	Samoa	Information not reported	1000	25	64	Yes	Yes	FFQ	Nationally representative
GDD 2017	Demographic and Health Survey Sao Tome and Principe 2008	2008	2009	Sao Tome And Principe	Both urban and rural	6589	0	59	Yes	Yes	DHS questionnair e	Nationally representative

GDD 2010	Sao Tom� et Principe Enqu�te STEPS 2009	2009	2009	Sao Tome And Principe	*Information not reported*	*1000*	*25*	*64*	*Yes*	*Yes*	*FFQ*	*Nationally representative*
GDD 2017	Premature biological aging in Saudi adults with Type 2 diabetes and the influence of endotoxinemia	2014	2015	Saudi Arabia	*Only urban*	*185*	*19*	*68*	*Yes*	*Yes*	*FFQ*	*Locally representative*
GDD 2017	Global Student Based Student Health Survey 2005	2005	2005	Senegal	*Both urban and rural*	*3101*	*11*	*16*	*Yes*	*Yes*	*FFQ*	*Nationally representative*
GDD 2017	World Health Survey 2003	2003	2003	Senegal	*Both urban and rural*	*1989*	*17*	*99*	*Yes*	*Yes*	*FFQ*	*Nationally representative*
GDD 2017	Demographic and Health Survey Senegal 2005	2005	2005	Senegal	*Both urban and rural*	*14950*	*0*	*59*	*Yes*	*Yes*	*DHS questionnaire*	*Nationally representative*
GDD 2017	Demographic and Health Survey Senegal 2008 MIS	2008	2009	Senegal	*Both urban and rural*	*9666*	*0*	*49*	*Yes*	*Yes*	*Biomarker*	*Nationally representative*
GDD 2017	Demographic and Health Survey Senegal 2010	2010	2011	Senegal	*Both urban and rural*	*19060*	*0*	*59*	*Yes*	*Yes*	*DHS questionnaire*	*Nationally representative*
GDD 2017	Demographic and Health Survey Senegal 2012	2012	2013	Senegal	*Both urban and rural*	*6241*	*0*	*4*	*Yes*	*Yes*	*DHS questionnaire*	*Nationally representative*
GDD 2017	Demographic and Health Survey Senegal 2014	2014	2014	Senegal	*Both urban and rural*	*6305*	*0*	*4*	*Yes*	*Yes*	*DHS questionnaire*	*Nationally representative*
GDD 2017	Demographic and Health Survey Senegal 2015	2015	2015	Senegal	*Both urban and rural*	*4290*	*0*	*4*	*Yes*	*Yes*	*DHS questionnaire*	*Nationally representative*
GDD 2017	MGSD Gestational Diabetes in the Mediterranean Region	2010	2013	Serbia	*Only rural*	*90*	*17*	*43*	*No*	*Yes*	*FFQ*	*Locally representative*
GDD 2010	Serbian Community Health and Nutrition Survey	2003	2003	Serbia	*Information not reported*	*1000*	*0*	*100*	*Yes*	*Yes*	*Household availability/ budget survey*	*Nationally representative*
GDD 2010	Serbian Community Health and Nutrition Survey	2004	2004	Serbia	*Information not reported*	*1000*	*0*	*100*	*Yes*	*Yes*	*Household availability/ budget survey*	*Nationally representative*
GDD 2017	Seychelles Heart Survey	2013	2015	Seychelles	*Both urban and rural*	*1240*	*25*	*64*	*Yes*	*Yes*	*FFQ*	*Nationally representative*

GDD 2017	Global Student Based Student Health Survey 2007	2007	2007	Seychelles	*Both urban and rural*	*1413*	*7*	*16*	*Yes*	*Yes*	*FFQ*	*Nationally representative*
GDD 2010	The Seychelles Heart Study 2004: main findings	2004	2004	Seychelles	*Information not reported*	*1000*	*25*	*64*	*Yes*	*Yes*	*FFQ*	*Nationally representative*
GDD 2017	Demographic and Health Survey Sierra Leone 2008	2008	2008	Sierra Leone	*Both urban and rural*	*12187*	*0*	*59*	*Yes*	*Yes*	*DHS questionnaire*	*Nationally representative*
GDD 2017	Demographic and Health Survey Sierra Leone 2013	2013	2013	Sierra Leone	*Both urban and rural*	*22800*	*0*	*59*	*Yes*	*Yes*	*DHS questionnaire*	*Nationally representative*
GDD 2010	The Prevalence of the Common Risk Factors of Non-Communicable Diseases in Sierra Leone	2009	2009	Sierra Leone	*Information not reported*	*1000*	*25*	*64*	*Yes*	*Yes*	*FFQ*	*Nationally representative*
GDD 2010	National Nutrition Survey 1998	1998	1998	Singapore	*Information not reported*	*1000*	*20*	*69*	*Yes*	*Yes*	*FFQ*	*Nationally representative*
GDD 2010	National Nutrition Survey 2004	2004	2004	Singapore	*Information not reported*	*1000*	*20*	*69*	*Yes*	*Yes*	*FFQ*	*Nationally representative*
GDD 2017	World Health Survey 2003	2003	2003	Slovakia	*Both urban and rural*	*1658*	*18*	*92*	*Yes*	*Yes*	*FFQ*	*Nationally representative*
GDD 2017	Health Behavior in School-Aged Children 1994	1993	1994	Slovakia	*Both urban and rural*	*3374*	*11*	*17*	*Yes*	*Yes*	*FFQ*	*Nationally representative*
GDD 2017	Health Behavior in School-Aged Children 1998	1998	1998	Slovakia	*Both urban and rural*	*3777*	*11*	*16*	*Yes*	*Yes*	*FFQ*	*Nationally representative*
GDD 2017	Health Behavior in School-Aged Children 2010	2010	2010	Slovakia	*Both urban and rural*	*5201*	*11*	*15*	*Yes*	*Yes*	*FFQ*	*Nationally representative*
GDD 2017	Health Behavior in School-Aged Children 2006	2006	2006	Slovakia	*Both urban and rural*	*3841*	*11*	*15*	*Yes*	*Yes*	*FFQ*	*Nationally representative*
GDD 2010	Slovakian Community Health and Nutrition Survey	1997	1997	Slovakia	*Information not reported*	*1000*	*0*	*100*	*Yes*	*Yes*	*Household availability/ budget survey*	*Nationally representative*
GDD 2010	Slovakian Community Health and Nutrition Survey	2000	2000	Slovakia	*Information not reported*	*1000*	*0*	*100*	*Yes*	*Yes*	*Household availability/ budget survey*	*Nationally representative*

GDD 2010	Slovakian Community Health and Nutrition Survey	2003	2003	Slovakia	*Information not reported*	*1000*	*0*	*100*	*Yes*	*Yes*	*Household availability/ budget survey*	*Nationally representative*
GDD 2010	Monitoring of the nutritional status of particular groups of adult population	2006	2006	Slovakia	*Information not reported*	*1000*	*19*	*54*	*Yes*	*Yes*	*Single 24-hour recall*	*Nationally representative*
GDD 2017	World Health Survey 2003	2003	2003	Slovenia	*Both urban and rural*	*565*	*18*	*91*	*Yes*	*Yes*	*FFQ*	*Nationally representative*
GDD 2017	Health Behavior in School-Aged Children 2002	2001	2002	Slovenia	*Both urban and rural*	*3915*	*11*	*17*	*Yes*	*Yes*	*FFQ*	*Nationally representative*
GDD 2017	Health Behavior in School-Aged Children 2006	2006	2006	Slovenia	*Both urban and rural*	*5100*	*11*	*15*	*Yes*	*Yes*	*FFQ*	*Nationally representative*
GDD 2017	Health Behavior in School-Aged Children 2010	2010	2010	Slovenia	*Both urban and rural*	*5413*	*11*	*15*	*Yes*	*Yes*	*FFQ*	*Nationally representative*
GDD 2010	Slovenian Community Health and Nutrition Survey	1998	1998	Slovenia	*Information not reported*	*1000*	*0*	*100*	*Yes*	*Yes*	*Household availability/ budget survey*	*Nationally representative*
GDD 2010	Slovenian Community Health and Nutrition Survey	2000	2000	Slovenia	*Information not reported*	*1000*	*0*	*100*	*Yes*	*Yes*	*Household availability/ budget survey*	*Nationally representative*
GDD 2010	Slovenian Community Health and Nutrition Survey	2002	2002	Slovenia	*Information not reported*	*1000*	*0*	*100*	*Yes*	*Yes*	*Household availability/ budget survey*	*Nationally representative*
GDD 2010	Nutritional status of Slovenian adolescens	2003	2005	Slovenia	*Information not reported*	*1010*	*14*	*17*	*Yes*	*Yes*	*FFQ*	*Nationally representative*
GDD 2010	Ribic et al.	2007	2007	Slovenia	*Information not reported*	*61*	*25*	*65*	*Yes*	*Yes*	*Biomarker*	*Nationally representative*
GDD 2017	Global Student Based Student Health Survey 2011	2011	2011	Solomon Islands	*Both urban and rural*	*1323*	*11*	*16*	*Yes*	*Yes*	*FFQ*	*Nationally representative*
GDD 2010	Changing patterns of hypertension, diabetes and diet among Melanesians and Micronesians in the Solomon Islands.-1985	2005	2005	Solomon Islands	*Information not reported*	*1000*	*15*	*64*	*Yes*	*Yes*	*FFQ*	*Sub-nationally representative*

Miller V, *et al. BMJ Global Health* 2021; 6:e003585. doi: 10.1136/bmjgh-2020-003585

GDD 2017	Transition and Health during Urbanisation of South Africans: Children	2000	2001	South Africa	*Both urban and rural*	*1257*	*9*	*16*	*Yes*	*Yes*	*Single 24-hour recall*	*Sub-nationally representative*
GDD 2017	Demographic and Health Survey South Africa 1998	1998	1998	South Africa	*Both urban and rural*	*3716*	*0*	*4*	*Yes*	*Yes*	*DHS questionnaire*	*Nationally representative*
GDD 2017	Study of Global Ageing and Adult Health	2007	2013	South Africa	*Both urban and rural*	*4007*	*18*	*113*	*Yes*	*Yes*	*FFQ*	*Nationally representative*
GDD 2017	World Health Survey 2003	2003	2003	South Africa	*Both urban and rural*	*1992*	*18*	*89*	*Yes*	*Yes*	*FFQ*	*Nationally representative*
GDD 2010	Food and nutrient availability in South African Households	1995	1995	South Africa	*Information not reported*	*1000*	*0*	*99*	*Yes*	*Yes*	*FFQ*	*Nationally representative*
GDD 2010	Charlton et al. (1 of 3)	2002	2002	South Africa	*Information not reported*	*110*	*20*	*65*	*Yes*	*Yes*	*Biomarker*	*Locally representative*
GDD 2010	Charlton et al. (2 of 3)	2002	2002	South Africa	*Information not reported*	*112*	*20*	*65*	*Yes*	*Yes*	*Biomarker*	*Locally representative*
GDD 2010	Charlton et al. (3 of 3)	2002	2002	South Africa	*Information not reported*	*103*	*20*	*65*	*Yes*	*Yes*	*Biomarker*	*Locally representative*
GDD 2010	Maseko et al.	2003	2003	South Africa	*Information not reported*	*291*	*21*	*72*	*Yes*	*Yes*	*Biomarker*	*Locally representative*
GDD 2010	Barlow et al. (1 of 2)	1982	1982	South Africa	*Information not reported*	*150*	*30*	*50*	*Yes*	*No*	*Biomarker*	*Locally representative*
GDD 2010	Barlow et al. (2 of 2)	1982	1982	South Africa	*Information not reported*	*64*	*30*	*50*	*Yes*	*No*	*Biomarker*	*Locally representative*
GDD 2010	Charlton et al. (1 of 3)	2002	2002	South Africa	*Information not reported*	*110*	*20*	*65*	*Yes*	*Yes*	*Single 24-hour recall*	*Locally representative*
GDD 2010	Charlton et al. (2 of 3)	2002	2002	South Africa	*Information not reported*	*112*	*20*	*65*	*Yes*	*Yes*	*Single 24-hour recall*	*Locally representative*
GDD 2010	Charlton et al. (3 of 3)	2002	2002	South Africa	*Information not reported*	*103*	*20*	*65*	*Yes*	*Yes*	*Single 24-hour recall*	*Locally representative*
GDD 2017	World Health Survey 2003	2003	2003	Spain	*Both urban and rural*	*5730*	*18*	*101*	*Yes*	*Yes*	*FFQ*	*Nationally representative*
GDD 2017	Health Behavior in School-Aged Children 1994	1993	1994	Spain	*Both urban and rural*	*4570*	*11*	*16*	*Yes*	*Yes*	*FFQ*	*Nationally representative*
GDD 2017	Health Behavior in School-Aged Children 2002	2001	2002	Spain	*Both urban and rural*	*5823*	*11*	*16*	*Yes*	*Yes*	*FFQ*	*Nationally representative*
GDD 2017	Health Behavior in School-Aged Children 2006	2005	2006	Spain	*Both urban and rural*	*8874*	*11*	*15*	*Yes*	*Yes*	*FFQ*	*Nationally representative*
GDD 2017	Health Behavior in School-Aged Children 2010	2009	2010	Spain	*Both urban and rural*	*5030*	*11*	*15*	*Yes*	*Yes*	*FFQ*	*Nationally representative*

GDD 2010	Spanish Community Health and Nutrition Survey	1980	1981	Spain	*Information not reported*	*1000*	*0*	*100*	*Yes*	*Yes*	*Household availability/ budget survey*	*Nationally representative*
GDD 2010	Spanish Community Health and Nutrition Survey	1990	1991	Spain	*Information not reported*	*1000*	*0*	*100*	*Yes*	*Yes*	*Household availability/ budget survey*	*Nationally representative*
GDD 2010	Spanish Community Health and Nutrition Survey	1998	1999	Spain	*Information not reported*	*1000*	*0*	*100*	*Yes*	*Yes*	*Household availability/ budget survey*	*Nationally representative*
GDD 2010	The Catalan Nutrition Survey (ENCAT 2002-2003) and Serra Majem and Ribas, 2007 and Serra-Majem L et al., 2007 and Serra Majem et al., 2006	2003	2003	Spain	*Information not reported*	*1000*	*10*	*100*	*Yes*	*Yes*	*Multiple 24-hour recall*	*Nationally representative*
GDD 2010	TRANSFAIR study	1991	1991	Spain	*Information not reported*	*1000*	*0*	*100*	*Yes*	*Yes*	*Household availability/ budget survey*	*Nationally representative*
GDD 2010	INTERSALT, Manresa	1985	1987	Spain	*Information not reported*	*25*	*20*	*59*	*Yes*	*Yes*	*Biomarker*	*Sub-nationally representative*
GDD 2010	INTERSALT, Torrejon,	1985	1987	Spain	*Information not reported*	*25*	*20*	*59*	*Yes*	*Yes*	*Biomarker*	*Sub-nationally representative*
GDD 2010	Schroder et al.	1994	1996	Spain	*Information not reported*	*1567*	*25*	*54*	*Yes*	*Yes*	*Multiple 24-hour recall*	*Sub-nationally representative*
GDD 2010	Ortega et al.	2009	2009	Spain	*Information not reported*	*196*	*20*	*60*	*Yes*	*Yes*	*Biomarker*	*Nationally representative*
GDD 2017	Lifestyle patterns and dysglycaemic risk in urban Sri Lankan women	2010	2012	Sri Lanka	*Only urban*	*617*	*30*	*45*	*No*	*Yes*	*FFQ*	*Locally representative*
GDD 2017	Nutrition Survey -DRU	2003	2006	Sri Lanka	*Both urban and rural*	*488*	*18*	*89*	*Yes*	*Yes*	*Single 24-hour recall*	*Nationally representative*
GDD 2017	Global Student Based Student Health Survey 2008	2008	2008	Sri Lanka	*Both urban and rural*	*2581*	*11*	*16*	*Yes*	*Yes*	*FFQ*	*Nationally representative*
GDD 2017	World Health Survey 2003	2003	2003	Sri Lanka	*Both urban and rural*	*6805*	*18*	*100*	*Yes*	*Yes*	*FFQ*	*Nationally representative*

GDD 2010	Abdominal obesity and its association with selected risk factors of coronary heart disease in an adult population in the district of Colombo; MD Thesis (Community Medicine), Postgraduate Institute of Medicine, University of Colombo, Sri Lanka	2004	2004	Sri Lanka	*Information not reported*	*1000*	*20*	*64*	*Yes*	*Yes*	*FFQ*	*Sub-nationally representative*
GDD 2010	Sri Lanka Global School-Based Student Health Survey (GSHS)-2007	2006	2006	Sri Lanka	*Information not reported*	*1000*	*15*	*64*	*Yes*	*Yes*	*FFQ*	*Nationally representative*
GDD 2017	Global Student Based Student Health Survey 2012	2012	2012	Sudan	*Both urban and rural*	*2115*	*11*	*16*	*Yes*	*Yes*	*FFQ*	*Nationally representative*
GDD 2017	Demographic and Health Survey Sudan 1989	1989	1990	Sudan	*Both urban and rural*	*2145*	*0*	*4*	*Yes*	*Yes*	*DHS questionnaire*	*Nationally representative*
GDD 2017	Global Student Based Student Health Survey 2009	2009	2009	Suriname	*Both urban and rural*	*1677*	*11*	*16*	*Yes*	*Yes*	*FFQ*	*Nationally representative*
GDD 2017	Global Student Based Student Health Survey 2003	2003	2003	Swaziland	*Both urban and rural*	*6771*	*11*	*16*	*Yes*	*Yes*	*FFQ*	*Nationally representative*
GDD 2017	Global Student Based Student Health Survey 2013	2013	2013	Swaziland	*Both urban and rural*	*3616*	*7*	*16*	*Yes*	*Yes*	*FFQ*	*Nationally representative*
GDD 2017	World Health Survey 2003	2003	2003	Swaziland	*Both urban and rural*	*3070*	*18*	*98*	*Yes*	*Yes*	*FFQ*	*Nationally representative*
GDD 2017	Demographic and Health Survey Swaziland 2006	2006	2007	Swaziland	*Both urban and rural*	*10641*	*0*	*49*	*Yes*	*Yes*	*DHS questionnaire*	*Nationally representative*
GDD 2017	Swedish National Dietary Survey-Riksmaten 2010-11	2010	2011	Sweden	*Both urban and rural*	*1797*	*18*	*80*	*Yes*	*Yes*	*Multiple 24-hour recall*	*Nationally representative*
GDD 2017	Health Behavior in School-Aged Children 1986	1985	1986	Sweden	*Both urban and rural*	*2933*	*11*	*15*	*Yes*	*Yes*	*FFQ*	*Nationally representative*
GDD 2017	Health Behavior in School-Aged Children 1990	1989	1990	Sweden	*Both urban and rural*	*3474*	*11*	*16*	*Yes*	*Yes*	*FFQ*	*Nationally representative*

Miller V, *et al. BMJ Global Health* 2021; 6:e003585. doi: 10.1136/bmjgh-2020-003585

GDD 2017	Health Behavior in School-Aged Children 1994	1993	1994	Sweden	*Both urban and rural*	*3552*	*11*	*17*	*Yes*	*Yes*	*FFQ*	*Nationally representative*
GDD 2017	Health Behavior in School-Aged Children 1998	1997	1998	Sweden	*Both urban and rural*	*3767*	*11*	*16*	*Yes*	*Yes*	*FFQ*	*Nationally representative*
GDD 2017	Health Behavior in School-Aged Children 2002	2001	2002	Sweden	*Both urban and rural*	*3896*	*11*	*16*	*Yes*	*Yes*	*FFQ*	*Nationally representative*
GDD 2017	Health Behavior in School-Aged Children 2006	2005	2006	Sweden	*Both urban and rural*	*4364*	*11*	*15*	*Yes*	*Yes*	*FFQ*	*Nationally representative*
GDD 2017	Health Behavior in School-Aged Children 2010	2009	2010	Sweden	*Both urban and rural*	*6582*	*11*	*15*	*Yes*	*Yes*	*FFQ*	*Nationally representative*
GDD 2010	Dietary habits and nutrient intake in Sweden 1989	1989	1989	Sweden	*Information not reported*	*1000*	*18*	*74*	*Yes*	*Yes*	*Single 24-hour recall*	*Nationally representative*
GDD 2010	Swedish Community Health and Nutrition Survey	1989	1989	Sweden	*Information not reported*	*1000*	*0*	*100*	*Yes*	*Yes*	*Household availability/ budget survey*	*Nationally representative*
GDD 2010	Swedish Community Health and Nutrition Survey	1996	1996	Sweden	*Information not reported*	*1000*	*18*	*74*	*Yes*	*Yes*	*Household availability/ budget survey*	*Nationally representative*
GDD 2010	Dietary habits and nutrient intake in Sweden 1997-98	1997	1998	Sweden	*Information not reported*	*1000*	*17*	*79*	*Yes*	*Yes*	*Single 24-hour recall*	*Nationally representative*
GDD 2010	Dietary habits and nutrient intake in Sweden 1997-98	1997	1998	Sweden	*Information not reported*	*1000*	*19*	*100*	*Yes*	*Yes*	*Single 24-hour recall*	*Nationally representative*
GDD 2010	Dietary habits and nutrient intake among Swedish children	2003	2003	Sweden	*Information not reported*	*1000*	*4*	*11*	*Yes*	*Yes*	*Single 24-hour recall*	*Nationally representative*
GDD 2010	TRANSFAIR study	1989	1989	Sweden	*Information not reported*	*500*	*19*	*64*	*Yes*	*Yes*	*Single 24-hour recall*	*Nationally representative*
GDD 2017	Bus Santé	1994	2012	Switzerland	*Both urban and rural*	*4842*	*35*	*75*	*Yes*	*Yes*	*FFQ*	*Sub-nationally representative*
GDD 2017	CoLaus	2009	2012	Switzerland	*Only urban*	*4249*	*35*	*75*	*Yes*	*Yes*	*FFQ*	*Locally representative*
GDD 2017	Health Behavior in School-Aged Children 1986	1985	1986	Switzerland	*Both urban and rural*	*4804*	*11*	*17*	*Yes*	*Yes*	*FFQ*	*Nationally representative*

Miller V, *et al. BMJ Global Health* 2021; 6:e003585. doi: 10.1136/bmjgh-2020-003585

GDD 2017	Health Behavior in School-Aged Children 1998	1997	1998	Switzerland	*Both urban and rural*	*5520*	*11*	*16*	*Yes*	*Yes*	*FFQ*	*Nationally representative*
GDD 2017	Health Behavior in School-Aged Children 2002	2001	2002	Switzerland	*Both urban and rural*	*4528*	*11*	*17*	*Yes*	*Yes*	*FFQ*	*Nationally representative*
GDD 2017	Health Behavior in School-Aged Children 2006	2005	2006	Switzerland	*Both urban and rural*	*4553*	*11*	*15*	*Yes*	*Yes*	*FFQ*	*Nationally representative*
GDD 2017	Health Behavior in School-Aged Children 2010	2009	2010	Switzerland	*Both urban and rural*	*6598*	*11*	*15*	*Yes*	*Yes*	*FFQ*	*Nationally representative*
GDD 2010	Etude Bus Sant�	2004	2009	Switzerland	*Information not reported*	*1000*	*35*	*74*	*Yes*	*Yes*	*FFQ*	*Locally representative*
GDD 2010	Chappuis et al.	2010	2011	Switzerland	*Information not reported*	*154*	*20*	*79*	*Yes*	*Yes*	*Biomarker*	*Nationally representative*
GDD 2017	Global Student Based Student Health Survey 2011	2011	2011	Syrian Arab Republic	*Both urban and rural*	*3089*	*11*	*16*	*Yes*	*Yes*	*FFQ*	*Nationally representative*
GDD 2017	Global Student Based Student Health Survey 2010	2010	2010	Syrian Arab Republic	*Both urban and rural*	*2085*	*11*	*16*	*Yes*	*Yes*	*FFQ*	*Nationally representative*
GDD 2010	2005-2008 Nutrition and Health Survey in Taiwan	2005	2008	Taiwan	*Information not reported*	*1000*	*19*	*100*	*Yes*	*Yes*	*Single 24-hour recall*	*Nationally representative*
GDD 2010	INTERSALT, San Chilo Village	1985	1987	Taiwan	*Information not reported*	*22*	*20*	*59*	*Yes*	*Yes*	*Biomarker*	*Sub-nationally representative*
GDD 2017	Demographic and Health Survey Tajikistan 2012	2012	2012	Tajikistan	*Both urban and rural*	*2960*	*0*	*4*	*Yes*	*Yes*	*DHS questionnair e*	*Nationally representative*
GDD 2017	Demographic and Health Survey Tanzania 2004-2005	2004	2005	Tanzania	*Both urban and rural*	*17743*	*0*	*49*	*Yes*	*Yes*	*DHS questionnair e*	*Nationally representative*
GDD 2017	Demographic and Health Survey Tanzania 2010	2010	2010	Tanzania	*Both urban and rural*	*17379*	*0*	*49*	*Yes*	*Yes*	*DHS questionnair e*	*Nationally representative*
GDD 2017	Global Student Based Student Health Survey 2006	2006	2006	Tanzania	*Only urban*	*2125*	*11*	*16*	*Yes*	*Yes*	*FFQ*	*Locally representative*
GDD 2017	Demographic and Health Survey Tanzania 1999	1999	1999	Tanzania	*Both urban and rural*	*1849*	*0*	*4*	*Yes*	*Yes*	*DHS questionnair e*	*Nationally representative*

GDD 2017	Demographic and Health Survey United Republic of Tanzania 2015	2015	2016	Tanzania	*Both urban and rural*	*22434*	*0*	*49*	*Yes*	*Yes*	*DHS questionnaire*	*Nationally representative*
GDD 2010	Pavan et al. (2 of 4)	1994	1994	Tanzania	*Information not reported*	*232*	*22*	*85*	*Yes*	*Yes*	*FFQ*	*Sub-nationally representative*
GDD 2017	Calcium and bone status in Thais	2010	2011	Thailand	*Only urban*	*845*	*11*	*88*	*Yes*	*Yes*	*Single 24-hour recall*	*Locally representative*
GDD 2017	SEANUTS: South East Asian Nutrition Surveys	2010	2011	Thailand	*Both urban and rural*	*3105*	*0*	*12*	*Yes*	*Yes*	*Single 24-hour recall*	*Nationally representative*
GDD 2017	Food Consumption Survey	2004	2005	Thailand	*Both urban and rural*	*7309*	*18*	*103*	*Yes*	*Yes*	*FFQ*	*Nationally representative*
GDD 2017	Global Student Based Student Health Survey 2008	2008	2008	Thailand	*Both urban and rural*	*2758*	*11*	*16*	*Yes*	*Yes*	*FFQ*	*Nationally representative*
GDD 2010	Calcium Status, Factors Affecting Calcium and Bone Status in Healthy Thais Living in Bangkok	1993	1993	Thailand	*Information not reported*	*250*	*20*	*80*	*Yes*	*Yes*	*Single 24-hour recall*	*Locally representative*
GDD 2010	The 4th National Food and Nutrition Survey, Thailand	1995	1995	Thailand	*Information not reported*	*1000*	*0*	*50*	*Yes*	*Yes*	*FFQ*	*Nationally representative*
GDD 2010	National Food and Nutrition Survey, Thailand	2003	2003	Thailand	*Information not reported*	*1000*	*15*	*74*	*Yes*	*Yes*	*Single 24-hour recall*	*Nationally representative*
GDD 2010	Fruit and vegetable consumption and its recommended intake associated with sociodemographic factors: Thailand National Health Examination Survey III	2004	2004	Thailand	*Information not reported*	*1000*	*15*	*100*	*Yes*	*Yes*	*FFQ*	*Nationally representative*
GDD 2010	Activity, dietary intake, and anthropometry of an informal social group of Thai elderly in Bangkok		2000	Thailand	*Information not reported*	*250*	*60*	*94*	*Yes*	*Yes*	*Multiple 24-hour recall*	*Locally representative*
GDD 2010	Kwanmaung et al. (1 of 2)	1998	1998	Thailand	*Information not reported*	*18*	*20*	*79*	*Yes*	*Yes*	*Biomarker*	*Locally representative*
GDD 2017	Demographic and Health Survey Timor-Leste 2009	2010	2010	Timor-Leste	*Both urban and rural*	*15615*	*0*	*49*	*Yes*	*Yes*	*DHS questionnaire*	*Nationally representative*
GDD 2017	Demographic and Health Survey Togo 2013	2013	2014	Togo	*Both urban and rural*	*14505*	*0*	*59*	*Yes*	*Yes*	*DHS questionnaire*	*Nationally representative*

Miller V, *et al. BMJ Global Health* 2021; 6:e003585. doi: 10.1136/bmjgh-2020-003585

GDD 2010	Tokelau STEPS Survey 2005	2005	2005	Tokelau	*Information not reported*	*500*	*15*	*64*	*Yes*	*Yes*	*FFQ*	*Nationally representative*
GDD 2017	Global Student Based Student Health Survey 2010	2010	2010	Tonga	*Both urban and rural*	*2186*	*11*	*16*	*Yes*	*Yes*	*FFQ*	*Nationally representative*
GDD 2010	Tonga STEPS Survey 2004 Fact Sheet	2004	2004	Tonga	*Information not reported*	*500*	*15*	*64*	*Yes*	*Yes*	*FFQ*	*Nationally representative*
GDD 2017	Food insecurity, food choices, and body mass index in adults: nutrition transition in Trinidad and Tobago 2002	2002	2002	Trinidad And Tobago	*Both urban and rural*	*545*	*24*	*89*	*Yes*	*Yes*	*FFQ*	*Sub-nationally representative*
GDD 2017	National Survey of Senior School Health	2003	2003	Trinidad And Tobago	*Both urban and rural*	*1973*	*15*	*17*	*Yes*	*Yes*	*FFQ*	*Nationally representative*
GDD 2017	National Health Survey of Primary School Children	2003	2003	Trinidad And Tobago	*Both urban and rural*	*3891*	*4*	*10*	*Yes*	*Yes*	*FFQ*	*Nationally representative*
GDD 2017	Food Consumption Patterns among Seventh-day Adventists Congregants in Trinidad and Tobago	2013	2014	Trinidad And Tobago	*Both urban and rural*	*187*	*0*	*6*	*Yes*	*Yes*	*FFQ*	*Locally representative*
GDD 2017	Global Student Based Student Health Survey 2007	2007	2007	Trinidad And Tobago	*Both urban and rural*	*2913*	*11*	*16*	*Yes*	*Yes*	*FFQ*	*Nationally representative*
GDD 2017	Global Student Based Student Health Survey 2011	2011	2011	Trinidad And Tobago	*Both urban and rural*	*2773*	*11*	*16*	*Yes*	*Yes*	*FFQ*	*Nationally representative*
GDD 2010	Food insecurity, food choices, and body mass index in adults: nutrition transition in Trinidad and Tobago	2001	2001	Trinidad And Tobago	*Information not reported*	*500*	*25*	*100*	*Yes*	*Yes*	*FFQ*	*Nationally representative*
GDD 2010	INTERSALT, Plymoth-Bethesda	1985	1987	Trinidad And Tobago	*Information not reported*	*21*	*20*	*59*	*Yes*	*Yes*	*Biomarker*	*Sub-nationally representative*
GDD 2017	MGSD Gestational Diabetes in the Mediterranean Region	2010	2013	Tunisia	*Only rural*	*92*	*21*	*42*	*No*	*Yes*	*FFQ*	*Locally representative*
GDD 2017	Tunisian Scholar Children Food Consumption Survey	2006	2006	Tunisia	*Both urban and rural*	*715*	*5*	*8*	*Yes*	*Yes*	*FFQ*	*Sub-nationally representative*
GDD 2017	Global Student Based Student Health Survey 2008	2008	2008	Tunisia	*Both urban and rural*	*2808*	*11*	*16*	*Yes*	*Yes*	*FFQ*	*Nationally representative*
GDD 2017	World Health Survey 2003	2003	2003	Tunisia	*Both urban and rural*	*4697*	*18*	*100*	*Yes*	*Yes*	*FFQ*	*Nationally representative*

GDD 2017	Salt Consumption Study in Turkey	2012	2012	Turkey	*Both urban and rural*	*657*	*18*	*90*	*Yes*	*Yes*	*Single 24-hour recall*	*Nationally representative*
GDD 2017	Determination of dietary habits as a risk factor of cardiovascular heart disease in Turkish adolescents	2003	2004	Turkey	*Only urban*	*300*	*12*	*19*	*Yes*	*Yes*	*Multiple 24-hour recall*	*Locally representative*
GDD 2017	World Health Survey 2003	2003	2003	Turkey	*Both urban and rural*	*10562*	*18*	*89*	*Yes*	*Yes*	*FFQ*	*Nationally representative*
GDD 2017	Health Behavior in School-Aged Children 2006	2005	2006	Turkey	*Both urban and rural*	*5477*	*11*	*15*	*Yes*	*Yes*	*FFQ*	*Nationally representative*
GDD 2017	Health Behavior in School-Aged Children 2010	2009	2010	Turkey	*Both urban and rural*	*5559*	*11*	*15*	*Yes*	*Yes*	*FFQ*	*Nationally representative*
GDD 2010	Dietary intake of adult population living in Ankara	2005	2005	Turkey	*Information not reported*	*1000*	*20*	*84*	*Yes*	*Yes*	*Single 24-hour recall*	*Locally representative*
GDD 2010	Erdem et al.	2007	2007	Turkey	*Information not reported*	*373*	*20*	*94*	*Yes*	*Yes*	*Biomarker*	*Nationally representative*
GDD 2017	Global Student Based Student Health Survey 2013	2013	2013	Tuvalu	*Both urban and rural*	*906*	*11*	*16*	*Yes*	*Yes*	*FFQ*	*Nationally representative*
GDD 2017	Demographic and Health Survey Uganda 2001	2000	2001	Uganda	*Both urban and rural*	*14053*	*0*	*54*	*Yes*	*Yes*	*DHS questionnaire*	*Nationally representative*
GDD 2017	Demographic and Health Survey Uganda 2006	2006	2006	Uganda	*Both urban and rural*	*12205*	*0*	*49*	*Yes*	*Yes*	*DHS questionnaire*	*Nationally representative*
GDD 2017	Demographic and Health Survey Uganda 2011	2011	2011	Uganda	*Both urban and rural*	*8190*	*0*	*49*	*Yes*	*Yes*	*DHS questionnaire*	*Nationally representative*
GDD 2017	Global Student Based Student Health Survey 2003	2003	2003	Uganda	*Both urban and rural*	*3048*	*11*	*16*	*Yes*	*Yes*	*FFQ*	*Nationally representative*
GDD 2017	DHS VII MIS	2014	2015	Uganda	*Both urban and rural*	*9122*	*0*	*95*	*Yes*	*Yes*	*DHS questionnaire*	*Nationally representative*
GDD 2017	DHS VII	2016	2016	Uganda	*Both urban and rural*	*23238*	*0*	*98*	*Yes*	*Yes*	*DHS questionnaire*	*Nationally representative*
GDD 2010	Pavan et al.(3 of 4)	1994	1994	Uganda	*Information not reported*	*138*	*22*	*89*	*Yes*	*Yes*	*FFQ*	*Sub-nationally representative*
GDD 2017	World Health Survey 2003	2003	2003	Ukraine	*Both urban and rural*	*2811*	*18*	*94*	*Yes*	*Yes*	*FFQ*	*Nationally representative*

GDD 2017	Health Behavior in School-Aged Children 2002	2001	2002	Ukraine	*Both urban and rural*	*4090*	*11*	*17*	*Yes*	*Yes*	*FFQ*	*Nationally representative*
GDD 2017	Health Behavior in School-Aged Children 2006	2005	2006	Ukraine	*Both urban and rural*	*5040*	*11*	*15*	*Yes*	*Yes*	*FFQ*	*Nationally representative*
GDD 2017	Health Behavior in School-Aged Children 2010	2009	2010	Ukraine	*Both urban and rural*	*5837*	*11*	*15*	*Yes*	*Yes*	*FFQ*	*Nationally representative*
GDD 2017	Demographic and Health Survey Ukraine 2007	2007	2007	Ukraine	*Both urban and rural*	*762*	*0*	*4*	*Yes*	*Yes*	*DHS questionnair e*	*Nationally representative*
GDD 2010	Living Conditions Lifestyles and Health	2001	2002	Ukraine	*Information not reported*	*1000*	*18*	*99*	*Yes*	*Yes*	*FFQ*	*Nationally representative*
GDD 2017	United Arab Emirate- Indoor Air, Health and Nutrition (UAE-IAHN) Survey	2009	2010	United Arab Emirates	*Both urban and rural*	*1011*	*6*	*50*	*Yes*	*Yes*	*Single 24-hour recall*	*Nationally representative*
GDD 2017	Global Student Based Student Health Survey 2005	2005	2005	United Arab Emirates	*Both urban and rural*	*15453*	*11*	*16*	*Yes*	*Yes*	*FFQ*	*Nationally representative*
GDD 2017	World Health Survey 2003	2003	2003	United Arab Emirates	*Both urban and rural*	*1183*	*18*	*90*	*Yes*	*Yes*	*FFQ*	*Nationally representative*
GDD 2017	Global Student Based Student Health Survey 2010	2010	2010	United Arab Emirates	*Both urban and rural*	*2547*	*11*	*16*	*Yes*	*Yes*	*FFQ*	*Nationally representative*
GDD 2017	Nutrition in African Population	1993	1996	United Kingdom	*Only urban*	*246*	*25*	*72*	*Yes*	*Yes*	*FFQ*	*Locally representative*
GDD 2017	Health Behavior in School-Aged Children 2002	2001	2002	United Kingdom	*Both urban and rural*	*14314*	*11*	*17*	*Yes*	*Yes*	*FFQ*	*Sub-nationally representative*
GDD 2017	Health Behavior in School-Aged Children 2006	2005	2006	United Kingdom	*Both urban and rural*	*15257*	*11*	*15*	*Yes*	*Yes*	*FFQ*	*Nationally representative*
GDD 2017	Health Behavior in School-Aged Children 2010	2009	2010	United Kingdom	*Both urban and rural*	*15578*	*11*	*15*	*Yes*	*Yes*	*FFQ*	*Nationally representative*
GDD 2017	Health Behavior in School-Aged Children 1986	1985	1986	United Kingdom	*Both urban and rural*	*4760*	*11*	*15*	*Yes*	*Yes*	*FFQ*	*Nationally representative*
GDD 2017	Health Behavior in School-Aged Children 1990	1989	1990	United Kingdom	*Both urban and rural*	*6712*	*11*	*17*	*Yes*	*Yes*	*FFQ*	*Sub-nationally representative*

GDD 2017	Health Behavior in School-Aged Children 1994	1993	1994	United Kingdom	*Both urban and rural*	*3950*	*11*	*17*	*Yes*	*Yes*	*FFQ*	*Sub-nationally representative*
GDD 2017	Health Behavior in School-Aged Children 1994	1993	1994	United Kingdom	*Both urban and rural*	*4924*	*11*	*17*	*Yes*	*Yes*	*FFQ*	*Sub-nationally representative*
GDD 2017	Health Behavior in School-Aged Children 1994	1993	1994	United Kingdom	*Both urban and rural*	*3855*	*11*	*17*	*Yes*	*Yes*	*FFQ*	*Sub-nationally representative*
GDD 2017	Health Behavior in School-Aged Children 1998	1997	1998	United Kingdom	*Both urban and rural*	*6344*	*11*	*17*	*Yes*	*Yes*	*FFQ*	*Sub-nationally representative*
GDD 2017	Health Behavior in School-Aged Children 1998	1997	1998	United Kingdom	*Both urban and rural*	*3346*	*11*	*16*	*Yes*	*Yes*	*FFQ*	*Sub-nationally representative*
GDD 2017	Health Behavior in School-Aged Children 1998	1997	1998	United Kingdom	*Both urban and rural*	*5588*	*11*	*17*	*Yes*	*Yes*	*FFQ*	*Sub-nationally representative*
GDD 2017	Health Behavior in School-Aged Children 1998	1997	1998	United Kingdom	*Both urban and rural*	*4516*	*11*	*17*	*Yes*	*Yes*	*FFQ*	*Sub-nationally representative*
GDD 2010	Dietary and Nutritional Survey of British Adults	1986	1987	United Kingdom	*Information not reported*	*1000*	*16*	*64*	*Yes*	*Yes*	*Single 24-hour recall*	*Nationally representative*
GDD 2010	National British Diet & Nutrition Survey	1986	1987	United Kingdom	*Information not reported*	*102*	*20*	*64*	*Yes*	*Yes*	*Biomarker*	*Nationally representative*
GDD 2010	National Diet and Nutrition Survey: children aged 1�-4� years	1992	1993	United Kingdom	*Information not reported*	*1000*	*2*	*5*	*Yes*	*Yes*	*Single 24-hour recall*	*Nationally representative*
GDD 2010	National Diet and Nutrition Survey: people aged 65 years and over	1994	1995	United Kingdom	*Information not reported*	*1000*	*65*	*100*	*Yes*	*Yes*	*Single 24-hour recall*	*Nationally representative*
GDD 2010	National Diet and Nutrition Survey: young people 4-18 years	1997	1997	United Kingdom	*Information not reported*	*1000*	*4*	*18*	*Yes*	*Yes*	*Single 24-hour recall*	*Nationally representative*
GDD 2010	National Diet and Nutrition Survey: adults aged 19-64 years	2000	2001	United Kingdom	*Information not reported*	*1000*	*19*	*64*	*Yes*	*Yes*	*Single 24-hour recall*	*Nationally representative*
GDD 2010	Low Income Diet and Health Survey, 2003-2005	2003	2005	United Kingdom	*Information not reported*	*1000*	*2*	*100*	*Yes*	*Yes*	*Multiple 24-hour recall*	*Nationally representative*
GDD 2010	TRANSFAIR study	1996	1996	United Kingdom	*Information not reported*	*1000*	*0*	*100*	*Yes*	*Yes*	*Household availability/*	*Nationally representative*

											budget survey	
GDD 2010	INTERSALT, Belfast	1985	1987	United Kingdom	*Information not reported*	*24*	*20*	*59*	*Yes*	*Yes*	*Biomarker*	*Sub-nationally representative*
GDD 2010	INTERSALT, Birmingham	1985	1987	United Kingdom	*Information not reported*	*25*	*20*	*59*	*Yes*	*Yes*	*Biomarker*	*Sub-nationally representative*
GDD 2010	INTERSALT, Bridgend, South-Wales	1985	1987	United Kingdom	*Information not reported*	*25*	*20*	*59*	*Yes*	*Yes*	*Biomarker*	*Sub-nationally representative*
GDD 2010	Fogarty et al.	1991	1991	United Kingdom	*Information not reported*	*25*	*20*	*74*	*Yes*	*Yes*	*Biomarker*	*Sub-nationally representative*
GDD 2010	Khaw et al.	1993	1997	United Kingdom	*Information not reported*	*160*	*45*	*79*	*Yes*	*Yes*	*Biomarker*	*Sub-nationally representative*
GDD 2010	National Centre for Social Research	2008	2008	United Kingdom	*Information not reported*	*85*	*20*	*64*	*Yes*	*Yes*	*Biomarker*	*Nationally representative*
GDD 2010	National Centre for Social Research	2007	2007	United Kingdom	*Information not reported*	*60*	*25*	*64*	*Yes*	*Yes*	*Biomarker*	*Nationally representative*
GDD 2010	Bingham et al.	1985	1985	United Kingdom	*Information not reported*	*71*	*25*	*44*	*Yes*	*Yes*	*Biomarker*	*Locally representative*
GDD 2010	National British Diet & Nutrition Survey	2000	2001	United Kingdom	*Information not reported*	*54*	*20*	*64*	*Yes*	*Yes*	*Biomarker*	*Nationally representative*
GDD 2010	Scotish center for Social Research	2009	2009	United Kingdom	*Information not reported*	*19*	*20*	*64*	*Yes*	*Yes*	*Biomarker*	*Sub-nationally representative*
GDD 2010	INTERMAP,Belfast	1997	1998	United Kingdom	*Information not reported*	*59*	*40*	*59*	*Yes*	*Yes*	*Biomarker*	*Sub-nationally representative*
GDD 2010	INTERMAP, West Bromwich	1997	1998	United Kingdom	*Information not reported*	*74*	*40*	*59*	*Yes*	*Yes*	*Biomarker*	*Sub-nationally representative*
GDD 2010	INTERMAP,Belfast	1997	1998	United Kingdom	*Information not reported*	*59*	*40*	*59*	*Yes*	*Yes*	*Single 24-hour recall*	*Sub-nationally representative*
GDD 2010	INTERMAP, West Bromwich	1997	1998	United Kingdom	*Information not reported*	*74*	*40*	*59*	*Yes*	*Yes*	*Single 24-hour recall*	*Sub-nationally representative*
GDD 2010	Khaw et al.	1993	1997	United Kingdom	*Information not reported*	*3423*	*45*	*79*	*Yes*	*Yes*	*Multiple 24-hour recall*	*Sub-nationally representative*
GDD 2017	Feeding Infants and Toddlers Study 2008	2008	2009	United States	*Both urban and rural*	*3273*	*0*	*4*	*No*	*No*	*Multiple 24-hour recall*	*Nationally representative*
GDD 2017	NHANES 2005-2006	2005	2006	United States	*Both urban and rural*	*9309*	*0*	*100*	*Yes*	*Yes*	*Multiple 24-hour recall*	*Nationally representative*
GDD 2017	NHANES 2007-2008	2007	2008	United States	*Both urban and rural*	*9221*	*0*	*100*	*Yes*	*Yes*	*Multiple 24-hour recall*	*Nationally representative*
GDD 2017	NHANES 2009-2010	2009	2010	United States	*Both urban and rural*	*9715*	*0*	*100*	*Yes*	*Yes*	*Multiple 24-hour recall*	*Nationally representative*
GDD 2017	NHANES 2011-2012	2011	2012	United States	*Both urban and rural*	*8476*	*0*	*100*	*Yes*	*Yes*	*Multiple 24-hour recall*	*Nationally representative*
GDD 2017	NHANES 2013-2014	2013	2014	United States	*Both urban and rural*	*8453*	*0*	*100*	*Yes*	*Yes*	*Multiple 24-hour recall*	*Nationally representative*

GDD 2017	NHANES 2015-2016	2015	2016	United States	*Both urban and rural*	*8453*	*0*	*100*	*Yes*	*Yes*	*Multiple 24-hour recall*	*Nationally representative*
GDD 2017	Health Behavior in School-Aged Children 1998	1997	1998	United States	*Both urban and rural*	*5169*	*11*	*16*	*Yes*	*Yes*	*FFQ*	*Nationally representative*
GDD 2017	Health Behavior in School-Aged Children 2002	2001	2002	United States	*Both urban and rural*	*5025*	*11*	*16*	*Yes*	*Yes*	*FFQ*	*Nationally representative*
GDD 2017	Health Behavior in School-Aged Children 2006	2005	2006	United States	*Both urban and rural*	*3860*	*11*	*15*	*Yes*	*Yes*	*FFQ*	*Nationally representative*
GDD 2017	Health Behavior in School-Aged Children 2010	2009	2010	United States	*Both urban and rural*	*6107*	*11*	*15*	*Yes*	*Yes*	*FFQ*	*Nationally representative*
GDD 2010	Continuing Survey of Food Intakes by Individuals (CSFII)	1989	1991	United States	*Information not reported*	*1000*	*3*	*100*	*Yes*	*Yes*	*Single 24-hour recall*	*Nationally representative*
GDD 2010	World-wide consumption of trans fatty acids	1981	1981	United States	*Information not reported*		*0*	*100*	*Yes*	*Yes*	*Household availability/ budget survey*	*Nationally representative*
GDD 2010	World-wide consumption of trans fatty acids	1984	1984	United States	*Information not reported*		*0*	*100*	*Yes*	*Yes*	*Household availability/ budget survey*	*Nationally representative*
GDD 2010	World-wide consumption of trans fatty acids	1985	1985	United States	*Information not reported*		*0*	*100*	*Yes*	*Yes*	*Single 24-hour recall*	*Nationally representative*
GDD 2010	World-wide consumption of trans fatty acids	1986	1986	United States	*Information not reported*		*0*	*100*	*Yes*	*Yes*	*Household availability/ budget survey*	*Nationally representative*
GDD 2010	World-wide consumption of trans fatty acids	1990	1990	United States	*Information not reported*		*0*	*100*	*Yes*	*Yes*	*Household availability/ budget survey*	*Nationally representative*
GDD 2010	World-wide consumption of trans fatty acids	1991	1991	United States	*Information not reported*		*0*	*100*	*Yes*	*Yes*	*Household availability/ budget survey*	*Nationally representative*
GDD 2010	World-wide consumption of trans fatty acids	1992	1992	United States	*Information not reported*		*0*	*100*	*Yes*	*Yes*	*Household availability/ budget survey*	*Nationally representative*

GDD 2010	World-wide consumption of trans fatty acids	1992	1992	United States	*Information not reported*		*0*	*100*	*Yes*	*Yes*	*Single 24-hour recall*	*Nationally representative*
GDD 2010	World-wide consumption of trans fatty acids	1992	1992	United States	*Information not reported*		*0*	*100*	*Yes*	*Yes*	*Single 24-hour recall*	*Nationally representative*
GDD 2010	World-wide consumption of trans fatty acids	1993	1993	United States	*Information not reported*		*0*	*100*	*Yes*	*Yes*	*Single 24-hour recall*	*Nationally representative*
GDD 2010	World-wide consumption of trans fatty acids	1994	1994	United States	*Information not reported*		*0*	*100*	*Yes*	*Yes*	*Single 24-hour recall*	*Nationally representative*
GDD 2010	World-wide consumption of trans fatty acids	1995	1995	United States	*Information not reported*		*0*	*100*	*Yes*	*Yes*	*Single 24-hour recall*	*Nationally representative*
GDD 2010	World-wide consumption of trans fatty acids	1999	1999	United States	*Information not reported*		*0*	*100*	*Yes*	*Yes*	*Single 24-hour recall*	*Nationally representative*
GDD 2010	Continuing Survey of Food Intakes by Individuals	1989	1989	United States	*Information not reported*	*1000*	*0*	*100*	*Yes*	*Yes*	*Multiple 24-hour recall*	*Nationally representative*
GDD 2010	Continuing Survey of Food Intakes by Individuals	1990	1990	United States	*Information not reported*	*1000*	*0*	*100*	*Yes*	*Yes*	*Multiple 24-hour recall*	*Nationally representative*
GDD 2010	Continuing Survey of Food Intakes by Individuals	1991	1991	United States	*Information not reported*	*1000*	*0*	*100*	*Yes*	*Yes*	*Multiple 24-hour recall*	*Nationally representative*
GDD 2010	Continuing Survey of Food Intakes by Individuals	1994	1994	United States	*Information not reported*	*1000*	*0*	*100*	*Yes*	*Yes*	*Multiple 24-hour recall*	*Nationally representative*
GDD 2010	Continuing Survey of Food Intakes by Individuals	1995	1995	United States	*Information not reported*	*1000*	*0*	*100*	*Yes*	*Yes*	*Multiple 24-hour recall*	*Nationally representative*
GDD 2010	Continuing Survey of Food Intakes by Individuals	1996	1996	United States	*Information not reported*	*1000*	*0*	*100*	*Yes*	*Yes*	*Multiple 24-hour recall*	*Nationally representative*
GDD 2010	NHANES 1999-2000	1999	2000	United States	*Information not reported*	*1000*	*0*	*100*	*Yes*	*Yes*	*Single 24-hour recall*	*Nationally representative*
GDD 2010	NHANES 2001-2002	2001	2002	United States	*Information not reported*	*1000*	*0*	*100*	*Yes*	*Yes*	*Multiple 24-hour recall*	*Nationally representative*
GDD 2010	NHANES 2003-2004	2003	2004	United States	*Information not reported*	*1000*	*0*	*100*	*Yes*	*Yes*	*Multiple 24-hour recall*	*Nationally representative*
GDD 2010	Cooper et al.	1991	1994	United States	*Information not reported*	*708*	*20*	*75*	*Yes*	*Yes*	*Biomarker*	*Sub-nationally representative*

GDD 2010	INTERSALT, Chicago	1985	1987	United States	*Information not reported*	*22*	*20*	*59*	*Yes*	*Yes*	*Biomarker*	*Sub-nationally representative*
GDD 2010	INTERSALT, Goodman, Blacks	1985	1987	United States	*Information not reported*	*22*	*20*	*59*	*Yes*	*Yes*	*Biomarker*	*Locally representative*
GDD 2010	INTERSALT, Goodman, Whites	1985	1987	United States	*Information not reported*	*24*	*20*	*59*	*Yes*	*Yes*	*Biomarker*	*Locally representative*
GDD 2010	INTERSALT, Molokai, Hawaii	1985	1987	United States	*Information not reported*	*25*	*20*	*59*	*Yes*	*Yes*	*Biomarker*	*Sub-nationally representative*
GDD 2010	INTERSALT, Jackson, Blacks	1985	1987	United States	*Information not reported*	*25*	*20*	*59*	*Yes*	*Yes*	*Biomarker*	*Locally representative*
GDD 2010	INTERSALT, Jackson, Whites	1985	1987	United States	*Information not reported*	*25*	*20*	*59*	*Yes*	*Yes*	*Biomarker*	*Locally representative*
GDD 2010	INTERMAP, City of Woodlawn, Baltimore, Maryland	1997	1998	United States	*Information not reported*	*72*	*40*	*59*	*Yes*	*Yes*	*Biomarker*	*Sub-nationally representative*
GDD 2010	INTERMAP, Chicago	1997	1998	United States	*Information not reported*	*75*	*40*	*59*	*Yes*	*Yes*	*Biomarker*	*Sub-nationally representative*
GDD 2010	INTERMAP, Honolulu	1997	1998	United States	*Information not reported*	*67*	*40*	*59*	*Yes*	*Yes*	*Biomarker*	*Locally representative*
GDD 2010	INTERMAP, Jackson	1997	1998	United States	*Information not reported*	*65*	*40*	*59*	*Yes*	*Yes*	*Biomarker*	*Locally representative*
GDD 2010	INTERMAP, Minneapolis	1997	1998	United States	*Information not reported*	*65*	*40*	*59*	*Yes*	*Yes*	*Biomarker*	*Sub-nationally representative*
GDD 2010	INTERMAP, Pittsburgh	1997	1998	United States	*Information not reported*	*67*	*40*	*59*	*Yes*	*Yes*	*Biomarker*	*Sub-nationally representative*
GDD 2010	INTERMAP, Corpus Christi, Hispanic	1997	1998	United States	*Information not reported*	*69*	*40*	*59*	*Yes*	*Yes*	*Biomarker*	*Locally representative*
GDD 2010	INTERMAP, Corpus Christi, Non-Hispanic	1997	1998	United States	*Information not reported*	*69*	*40*	*59*	*Yes*	*Yes*	*Biomarker*	*Locally representative*
GDD 2010	Taylor et al. (1 of 2)	1994	1999	United States	*Information not reported*	*330*	*40*	*75*	*No*	*Yes*	*Biomarker*	*Locally representative*
GDD 2010	Taylor et al. (2 of 2)	2005	2005	United States	*Information not reported*	*146*	*40*	*75*	*No*	*Yes*	*Biomarker*	*Locally representative*
GDD 2010	Holbrook et al.	1981	1981	United States	*Information not reported*	*12*	*20*	*53*	*Yes*	*Yes*	*Biomarker*	*Locally representative*
GDD 2010	INTERMAP, City of Woodlawn, Baltimore, Maryland	1997	1998	United States	*Information not reported*	*72*	*40*	*59*	*Yes*	*Yes*	*Single 24-hour recall*	*Sub-nationally representative*
GDD 2010	INTERMAP, Chicago	1997	1998	United States	*Information not reported*	*75*	*40*	*59*	*Yes*	*Yes*	*Single 24-hour recall*	*Sub-nationally representative*
GDD 2010	INTERMAP, Honolulu	1997	1998	United States	*Information not reported*	*67*	*40*	*59*	*Yes*	*Yes*	*Single 24-hour recall*	*Locally representative*
GDD 2010	INTERMAP, Jackson	1997	1998	United States	*Information not reported*	*65*	*40*	*59*	*Yes*	*Yes*	*Single 24-hour recall*	*Locally representative*

GDD 2010	INTERMAP, Minneapolis	1997	1998	United States	*Information not reported*	*65*	*40*	*59*	*Yes*	*Yes*	*Single 24-hour recall*	*Sub-nationally representative*
GDD 2010	INTERMAP, Pittsburgh	1997	1998	United States	*Information not reported*	*67*	*40*	*59*	*Yes*	*Yes*	*Single 24-hour recall*	*Sub-nationally representative*
GDD 2010	INTERMAP, Corpus Christi, Hispanic	1997	1998	United States	*Information not reported*	*69*	*40*	*59*	*Yes*	*Yes*	*Single 24-hour recall*	*Locally representative*
GDD 2010	INTERMAP, Corpus Christi, Non-Hispanic	1997	1998	United States	*Information not reported*	*69*	*40*	*59*	*Yes*	*Yes*	*Single 24-hour recall*	*Locally representative*
GDD 2010	Holbrook et al.	1981	1981	United States	*Information not reported*	*12*	*20*	*53*	*Yes*	*Yes*	*Multiple 24-hour recall*	*Locally representative*
GDD 2010	Espeland et al.	1992	1995	United States	*Information not reported*	*325*	*60*	*79*	*Yes*	*Yes*	*Biomarker*	*Sub-nationally representative*
GDD 2010	Espeland et al.	1992	1995	United States	*Information not reported*	*325*	*60*	*79*	*Yes*	*Yes*	*Single 24-hour recall*	*Sub-nationally representative*
GDD 2017	Global Student Based Student Health Survey 2006	2006	2006	Uruguay	*Both urban and rural*	*3377*	*11*	*16*	*Yes*	*Yes*	*FFQ*	*Nationally representative*
GDD 2017	Global Student Based Student Health Survey 2012	2012	2012	Uruguay	*Both urban and rural*	*3466*	*11*	*16*	*Yes*	*Yes*	*FFQ*	*Nationally representative*
GDD 2017	World Health Survey 2003	2003	2003	Uruguay	*Both urban and rural*	*2986*	*18*	*93*	*Yes*	*Yes*	*FFQ*	*Nationally representative*
GDD 2010	Uruguay Global School-Based Student Health Survey (GSHS)-2006	2006	2006	Uruguay	*Information not reported*	*1000*	*25*	*64*	*Yes*	*Yes*	*FFQ*	*Nationally representative*
GDD 2017	Demographic and Health Survey Uzbekistan 1996	1996	1996	Uzbekistan	*Both urban and rural*	*5506*	*0*	*49*	*Yes*	*Yes*	*DHS questionnaire*	*Nationally representative*
GDD 2017	Global Student Based Student Health Survey 2011	2011	2011	Vanuatu	*Both urban and rural*	*1088*	*11*	*16*	*Yes*	*Yes*	*FFQ*	*Nationally representative*
GDD 2010	STEP Vanuatu	2005	2005	Vanuatu	*Information not reported*	*1000*	*15*	*60*	*Yes*	*Yes*	*FFQ*	*Sub-nationally representative*
GDD 2017	Nutritional status in pregnant women under privileged communities of Caracas	1998	2000	Venezuela	*Only urban*	*197*	*13*	*44*	*No*	*Yes*	*Single 24-hour recall*	*Locally representative*
GDD 2017	Global Student Based Student Health Survey 2003	2003	2003	Venezuela	*Both urban and rural*	*4294*	*11*	*16*	*Yes*	*Yes*	*FFQ*	*Nationally representative*

Miller V, *et al. BMJ Global Health* 2021; 6:e003585. doi: 10.1136/bmjgh-2020-003585

GDD 2017	Qualitative and quantitative assessment of nutritional status and lifestyles of Vietnamese adolescents	2006	2006	Viet Nam	*Both urban and rural*	*1172*	*15*	*19*	*Yes*	*Yes*	*Single 24-hour recall*	*Sub-nationally representative*
GDD 2017	SEANUTS: South East Asian Nutrition Surveys	2010	2011	Viet Nam	*Both urban and rural*	*2861*	*0*	*11*	*Yes*	*Yes*	*Single 24-hour recall*	*Nationally representative*
GDD 2017	Global Student Based Student Health Survey 2013	2013	2013	Viet Nam	*Both urban and rural*	*3316*	*7*	*16*	*Yes*	*Yes*	*FFQ*	*Nationally representative*
GDD 2017	Demographic and Health Survey Vietnam 1997	1997	1997	Viet Nam	*Both urban and rural*	*1597*	*0*	*2*	*Yes*	*Yes*	*DHS questionnaire*	*Nationally representative*
GDD 2017	World Health Survey 2003	2003	2003	Viet Nam	*Both urban and rural*	*4174*	*18*	*95*	*Yes*	*Yes*	*FFQ*	*Nationally representative*
GDD 2017	Global Student Based Student Health Survey 2009	2009	2009	Virgin Islands, British	*Both urban and rural*	*1646*	*11*	*16*	*Yes*	*Yes*	*FFQ*	*Nationally representative*
GDD 2010	The Virgin Islands (UK) STEPS Survey 2009	2009	2009	Virgin Islands, British	*Information not reported*	*1000*	*25*	*64*	*Yes*	*Yes*	*FFQ*	*Nationally representative*
GDD 2017	Global Student Based Student Health Survey 2008	2008	2008	Yemen	*Both urban and rural*	*1051*	*11*	*16*	*Yes*	*Yes*	*FFQ*	*Nationally representative*
GDD 2017	Demographic and Health Survey Yemen 1991	1991	1992	Yemen	*Both urban and rural*	*2114*	*0*	*4*	*Yes*	*Yes*	*DHS questionnaire*	*Nationally representative*
GDD 2017	Demographic and Health Survey Yemen 2013	2013	2013	Yemen	*Both urban and rural*	*22752*	*0*	*49*	*Yes*	*Yes*	*DHS questionnaire*	*Nationally representative*
GDD 2017	Global Student Based Student Health Survey 2004	2004	2004	Zambia	*Both urban and rural*	*2098*	*11*	*16*	*Yes*	*Yes*	*FFQ*	*Nationally representative*
GDD 2017	Demographic and Health Survey Zambia 1996	1996	1997	Zambia	*Both urban and rural*	*4015*	*0*	*4*	*Yes*	*Yes*	*DHS questionnaire*	*Nationally representative*
GDD 2017	World Health Survey 2003	2003	2003	Zambia	*Both urban and rural*	*3502*	*18*	*99*	*Yes*	*Yes*	*FFQ*	*Nationally representative*
GDD 2017	Demographic and Health Survey Zambia 2001	2001	2002	Zambia	*Both urban and rural*	*5165*	*0*	*4*	*Yes*	*Yes*	*DHS questionnaire*	*Nationally representative*
GDD 2017	Demographic and Health Survey Zambia 2007	2007	2007	Zambia	*Both urban and rural*	*8220*	*0*	*49*	*Yes*	*Yes*	*DHS questionnaire*	*Nationally representative*

GDD 2017	Demographic and Health Survey Zambia 2013	2013	2014	Zambia	*Both urban and rural*	*7821*	*0*	*4*	*Yes*	*Yes*	*DHS questionnaire*	*Nationally representative*
GDD 2017	Demographic and Health Survey Zambia 1992	1992	1992	Zambia	*Both urban and rural*	*2101*	*0*	*4*	*Yes*	*Yes*	*DHS questionnaire*	*Nationally representative*
GDD 2010	STEP Zambia	2008	2008	Zambia	*Information not reported*	*1000*	*25*	*100*	*Yes*	*Yes*	*FFQ*	*Sub-nationally representative*
GDD 2017	Demographic and Health Survey Zimbabwe 1994	1994	1994	Zimbabwe	*Both urban and rural*	*2019*	*0*	*2*	*Yes*	*Yes*	*DHS questionnaire*	*Nationally representative*
GDD 2017	World Health Survey 2003	2003	2003	Zimbabwe	*Both urban and rural*	*4264*	*18*	*108*	*Yes*	*Yes*	*FFQ*	*Nationally representative*
GDD 2017	Demographic and Health Survey Zimbabwe 1999	1999	1999	Zimbabwe	*Both urban and rural*	*3143*	*0*	*4*	*Yes*	*Yes*	*DHS questionnaire*	*Nationally representative*
GDD 2017	Demographic and Health Survey Zimbabwe 2006	2005	2006	Zimbabwe	*Both urban and rural*	*6286*	*0*	*49*	*Yes*	*Yes*	*DHS questionnaire*	*Nationally representative*
GDD 2017	Demographic and Health Survey Zimbabwe 2011	2010	2011	Zimbabwe	*Both urban and rural*	*19084*	*0*	*54*	*Yes*	*Yes*	*DHS questionnaire*	*Nationally representative*
GDD 2017	Demographic and Health Survey South Africa 1998	1988	1989	Zimbabwe	*Both urban and rural*	*2971*	*0*	*4*	*Yes*	*Yes*	*DHS questionnaire*	*Nationally representative*
GDD 2017	Demographic and Health Survey Zimbabwe 2015	2015	2015	Zimbabwe	*Both urban and rural*	*14447*	*0*	*49*	*Yes*	*Yes*	*DHS questionnaire*	*Nationally representative*
GDD 2010	INTERSALT, Harare	1985	1987	Zimbabwe	*Information not reported*	*25*	*20*	*59*	*Yes*	*Yes*	*Biomarker*	*Sub-nationally representative*

References

1. Dodd H, Williams S, Brown R, Venn B. Calculating meal glycemic index by using measured and published food values compared with directly measured meal glycemic index. Am J Clin Nutr. 2011;94(4):992-996.
2. Khatibzadeh S, Saheb Kashaf M, Micha R, Fahimi S, Shi P, Elmadfa I, Kalantarian S, Wirojratana P, Ezzati M, Powles J, Mozaffarian D. A global database of food and nutrient consumption. *Bulletin of the World Health Organization*. 2016;94(12):931-934
3. US Department of Agriculture, Agricultural Research Service, Nutrient Data Laboratory. USDA National Nutrient Database for Standard Reference, Release 28. Version Current: September 2015, slightly revised May 2016.
4. Smith LC, Subandoro A. 2007. Measuring Food Security Using Household Expenditure Surveys. Food Security in Practice technical guide series. Washington, D.C.: International Food Policy Research Institute.
5. Willett W. *Nutritional Epidemiology. Chapter 11: "Implications for Total Energy Intake for Epidemiologic Analyses"*. New York: Oxford University Press; 1998.
6. U.S. Department of Health and Human Services and U.S. Department of Agirculture. 2015-2020 Dietary Guidelines for Americans. 2015. https://health.gov/sites/default/files/2019-09/2015-2020_Dietary_Guidelines.pdf
7. CDC. National Health and Nutrition Examination Survey. https://wwwn.cdc.gov/nchs/nhanes/default.aspx
8. British Nutrition Foundation. Nutrition Requirements. 2019. https://www.nutrition.org.uk/attachments/article/261/Nutrition%20Requirements_Revised%20August%202019.pdf
9. National Institute of Nutrition. *Dietary Guidelines for Indians: A Manual.* Indian Council of Medical Research. 2011. https://www.nin.res.in/downloads/DietaryGuidelinesforNINwebsite.pdf
10. Del Gobbo LC, Khatibzadeh S, Imamura F, Micha R, Shi P, Smith M, Myers SS, Mozaffarian D. Assessing global dietary habits: a comparison of national estimates from the FAO and the Global Dietary Database. *The American Journal of Clinical Nutrition*. 2015;1010(5):1038-1046.
11. USDA. ChooseMyPlate. 2020. http://www.choosemyplate.gov
12. Lim SS, Vos T, Flaxman AD, Danaei G, Shibuya K, Adair-Rohani H, et al. A comparative risk assessment of burden of disease and injury attributable to 67 risk factors and risk factor clusters in 21 regions, 1990-2010: a systematic analysis for the Global Burden of Disease Study. 2010. Lancet 2013;380:2224-60.
13. Dietary Guidelines Advisory Committee. 2010 dietary guidelines for Americans. 2010. www.cnpp.usda.gov/DGAs2010-PolicyDocument.htm
14. World Health Organization. Guideline: sugars intake for adults and children. 2015. http://apps.who.int/iris/bitstream/10665/149782/1/9789241549028_eng.pdf?ua=1
Joint FAO/WHO/UNU Expert Consultation. Protein and Amino Acid Requirements in Human Nutrition. 2002. http://apps.who.int/iris/bitstream/10665/43411/1/WHO_TRS_935_eng.pdf
15. EFSA Panel on Dietetic Products, Nutrition, and Allergies (NDA); Scientific Opinion on Dietary Reference Values for carbohydrates and dietary fibre. *EFSA Journal* 2010; 8(3):1462 [77 pp.]. doi:10.2903/j.efsa.2010.1462.
16. EFSA Panel on Dietetic Products, Nutrition, and Allergies (NDA); Scientific Opinion on Dietary Reference Values for fats, including saturated fatty acids, polyunsaturated fatty acids, monounsaturated fatty acids, trans fatty acids, and cholesterol. *EFSA Journal* 2010; 8(3):1461. [107 pp.]. doi:10.2903/j.efsa.2010.1461.
17. FAO. Fats and fatty acids in human nutrition. Report of an expert consultation. 2010. https://www.who.int/nutrition/publications/nutrientrequirements/fatsandfattyacids_humannutrition/en/
18. EFSA Scientific Committee on Food and Scientific Panel on Dietetic Products, Nutrition, and Allergies (NDA). Tolerable upper intake levels for vitamins and minerals. 2006. http://www.efsa.europa.eu/sites/default/files/assets/ndatolerableuil.pdf
19. Mayo Clinic. Potassium supplement (oral route, parenteral route). 2020. https://www.mayoclinic.org/drugs-supplements/potassium-supplement-oral-route-parenteral-route/description/drg-20070753
20. NIH. Calcium fact sheet for health professionals. 2020. https://ods.od.nih.gov/factsheets/Calcium-HealthProfessional/#h2
21. NIH. Iron fact sheet for health professionals. 2020. https://ods.od.nih.gov/factsheets/Iron-HealthProfessional/
22. Joint FAO/WHO Expert Consultation on Human Vitamin and Mineral Requirements. 1998. http://apps.who.int/iris/bitstream/10665/42716/1/9241546123.pdf
23. NIH. Zinc fact sheet for health professionals. 2020. https://ods.od.nih.gov/factsheets/Zinc-HealthProfessional/#h2)
24. NIH. Magnesium fact sheet for health professionals. 2020. https://ods.od.nih.gov/factsheets/Magnesium-HealthProfessional/
25. NIH. Iodine https://ods.od.nih.gov/factsheets/Iodine-HealthProfessional/
26. NIH. Vitamin A fact sheet for health professionals. 2020. https://ods.od.nih.gov/factsheets/VitaminA-HealthProfessional/
27. NIH. Vitamin D fact sheet for health professionals. 2020. https://ods.od.nih.gov/factsheets/VitaminD-HealthProfessional/
28. NIH. Vitamin E fact sheet for health professionals. 2020. https://ods.od.nih.gov/factsheets/VitaminE-HealthProfessional/#h2
29. NIH. Vitamin C fact sheet for health professionals. 2020. https://ods.od.nih.gov/factsheets/VitaminC-HealthProfessional/
30. NIH. Thiamine fact sheet for health professionals. 2020. https://ods.od.nih.gov/factsheets/Thiamin-HealthProfessional/
31. NIH. Riboflavin fact sheet for health professionals. 2020. https://ods.od.nih.gov/factsheets/Riboflavin-HealthProfessional/
32. U.S. National Library of Medicine. Niacin. 2019. https://www.nlm.nih.gov/medlineplus/ency/article/002409.htm
33. NIH. Vitamin C fact sheet for health professionals. 2020. https://ods.od.nih.gov/factsheets/VitaminB6-HealthProfessional/
34. NIH. Folate fact sheet for health professionals. 2020. https://ods.od.nih.gov/factsheets/Folate-HealthProfessional/
